# The early inflorescence of *Arabidopsis thaliana* demonstrates positional effects in floral organ growth and meristem patterning

**DOI:** 10.1007/s00497-017-0320-3

**Published:** 2017-12-20

**Authors:** Andrew R. G. Plackett, Stephen J. Powers, Andy L. Phillips, Zoe A. Wilson, Peter Hedden, Stephen G. Thomas

**Affiliations:** 1 Department of Plant Sciences, Rothamsted Research, Harpenden, Hertfordshire AL5 2JQ UK; 2 Department of Computational and Analytical Sciences, Rothamsted Research, Harpenden, Hertfordshire AL5 2JQ UK; 3 School of Biosciences, University of Nottingham, Loughborough, Leicestershire LE12 5RD UK; 4 Laboratory of Growth Regulators, Palacký University and Institute of Experimental Botany AS CR, Šlechtitelů 27, Olomouc, 783 71 Czech Republic; 5 Present Address: Department of Plant Sciences, University of Cambridge, Downing Street, Cambridge, CB2 3EA UK

**Keywords:** *Arabidopsis*, Flower, Inflorescence, Modelling, Gibberellin (GA)

## Abstract

**Key message:**

Linear modelling approaches detected significant gradients in organ growth and patterning across early flowers of the *Arabidopsis* inflorescence and uncovered evidence of new roles for gibberellin in floral development.

**Abstract:**

Most flowering plants, including the genetic model *Arabidopsis thaliana*, produce multiple flowers in sequence from a reproductive shoot apex to form a flower spike (inflorescence). The development of individual flowers on an *Arabidopsis* inflorescence has typically been considered as highly stereotypical and uniform, but this assumption is contradicted by the existence of mutants with phenotypes visible in early flowers only. This phenomenon is demonstrated by mutants partially impaired in the biosynthesis of the phytohormone gibberellin (GA), in which floral organ growth is retarded in the first flowers to be produced but has recovered spontaneously by the 10th flower. We presently lack systematic data from multiple flowers across the *Arabidopsis* inflorescence to explain such changes. Using mutants of the *GA 20*-*OXIDASE* (*GA20ox*) GA biosynthesis gene family to manipulate endogenous GA levels, we investigated the dynamics of changing floral organ growth across the early *Arabidopsis* inflorescence (flowers 1–10). Modelling of floral organ lengths identified a significant, GA-independent gradient of increasing stamen length relative to the pistil in the wild-type inflorescence that was separable from other, GA-dependent effects. It was also found that the first flowers exhibited unstable organ patterning in contrast to later flowers and that this instability was prolonged by exogenous GA treatment. These findings indicate that the development of individual flowers is influenced by hitherto unknown factors acting across the inflorescence and also suggest novel functions for GA in floral patterning.

**Electronic supplementary material:**

The online version of this article (10.1007/s00497-017-0320-3) contains supplementary material, which is available to authorized users.

## Introduction

The development of multiple flowers from a single growth axis to form a flower spike (inflorescence) is a common characteristic of many flowering plant taxa. The model plant *Arabidopsis thaliana* develops an indeterminate, raceme-type inflorescence (Prusinkiewicz et al. 2007), comprising individual lateral flowers arising immediately and sequentially from an apical inflorescence meristem (IM). Floral development in *Arabidopsis* follows a well-defined programme of events (Smyth et al. 1990) that gives rise to a stereotypical floral structure comprising a fixed sequence of concentric whorls with fixed numbers of floral organs (four sepals, four petals, six stamens and a central pistil). Organ identity within whorls is determined through overlapping expression of (and interaction between) different MADS-box genes following the ABCE model, with different whorls and organs within whorls separated by the expression of boundary genes (reviewed by Airoldi 2010; Irish 2010).

Despite this apparent uniformity, previous observations in *Arabidopsis* suggest that a flower’s development is influenced by its position on the inflorescence. Mutants impaired in biosynthesis of the plant hormone gibberellin (GA) via losses of members of the *GA 3*-*OXIDASE* (*GA3ox*) or *GA 20*-*OXIDASE* (*GA20ox*) gene families demonstrate fertility and growth defects in early flowers, but not later flowers on the same plant (Hu et al. 2008; Rieu et al. 2008). Whilst GA 3-oxidase catalyses the last step in bioactive GA biosynthesis (Chiang et al. 1995; Itoh et al. 1999), in *Arabidopsis* GA 20-oxidase enzymes catalyse the penultimate, rate-limiting step in the GA biosynthesis pathway (Huang et al. 1998; Coles et al. 1999), and the GA20ox substrate GA_12_ has recently been shown to act as a long-distance growth signal in *Arabidopsis* (Regnault et al. 2015). Loss of three *GA20ox* paralogues, *GA20ox1*, *GA20ox* *2* and *GA20ox* *3*, results in an infertile, GA-deficient floral phenotype in which floral organ growth is stunted (Plackett et al. 2012). In contrast, the *ga20ox1 ga20ox2* mutant demonstrates a failure in silique-set specifically in early flowers associated with reduced stamen growth relative to the pistil, but this phenotype recovers spontaneously through an unknown mechanism (Rieu et al. 2008). It has been inferred that the observed infertility in early *ga20ox1 ga20ox2* flowers is caused by this mismatched growth‚ preventing pollination and thus silique-set. In a second mutant with a similar phenotype, *ga3ox1 ga3ox3*, the recovery of successful silique-set in later flowers was also associated with an increase in stamen growth as flowering progressed (Hu et al. 2008). Changes in floral growth with changing position of flowers along the inflorescence have thus been demonstrated, but the mechanisms underlying those changes remain unclear.

GA itself is a candidate regulator for this process, with known roles in promoting floral organ growth and development (reviewed in Plackett et al. 2011), but the existing evidence remains inconclusive. A comparison of bioactive GA content between early (infertile) and later (fertile) *ga3ox1 ga3ox3* whole floral clusters did not detect a significant difference in GA levels, suggesting that the observed phenotypic changes do not relate directly to GA (Hu et al. 2008). That said, individual, tissue-specific expression patterns have been found for each *GA3ox* paralogue (Mitchum et al. 2006; Hu et al. 2008) as has a complex feedback mechanism dynamically regulating the expression of different paralogues within and between the *GA20ox* and *GA3ox* gene families (Rieu et al. 2008). In floral tissues, there is differential expression between *GA20ox1* and *GA20ox2* in the stamen filament and pistil, respectively, and up-regulation of *GA20ox3* in the *ga20ox1 ga20ox2* background (Plackett et al. 2012). Local changes in GA biosynthesis that affect specific organ development may thus have been missed by analyses at the whole flower level. Against this argument, spontaneous recovery of floral organ growth and silique-set has been reported during very late flowering of the GA-deficient mutant *ga1*-*3* (Cheng et al. 2004; Plackett et al. 2012), in which GA biosynthesis is entirely blocked at an early stage (Silverstone et al. 1997). However, experimental contamination by bioactive GA or the volatile GA precursor *ent*-kaurene (leading to endogenous GA biosynthesis in *ga1*-*3*) could not be conclusively ruled out under these circumstances (King et al. 2001; Otsuka et al. 2004; Silverstone et al. 2001).

To determine the contribution of GA to changes in floral organ growth across the early inflorescence, we conducted a detailed analysis of floral phenotypes in wild type (Col-0) and all combinations of *ga20ox1*, *ga20ox2* and *ga20ox3* loss-of-function mutant alleles across the early inflorescence, taking advantage of the phenotypic variation these mutants provide: the *ga20ox1* single mutant shows significant reductions in silique-set across this range, but less than *ga20ox1 ga20ox2* (Rieu et al. 2008), whereas the *ga20ox1 ga20ox2 ga20ox3* floral phenotype is similar to that of *ga1*-*3* (Plackett et al. 2012). Whilst different genotypes displayed changes to their silique-set frequency to a greater or lesser extent across the early inflorescence, our analysis identified a common trend between them of an increasing probability of silique-set with advancing flower position, irrespective of genotype or GA status. This is correlated with a significant gradient of increasing stamen length (both relative to the pistil and in absolute terms) with advancing flower position under control growth conditions in the wild-type inflorescence and most *ga20ox* genotypes, including *ga20ox1 ga20ox2 ga20ox3*. The observed reduced growth of stamens (and petals) in early, non-silique-setting flowers of *ga20ox1 ga20ox2* was independent from this underlying gradient and instead correlated with a failure of anther dehiscence in these flowers. Thus, GA-dependent and GA-independent components were identified in association with changing floral organ growth and development across the early inflorescence.

Unexpectedly, we found that development in the earliest flowers to form on the Col-0 wild-type inflorescence was atypical, with significantly more aberrations in organ number and structure occurring than in later flowers. Patterning became highly stereotypical in later flowers under control growth conditions, but remained unstable under exogenous GA treatment. A genetic component was identified, with *ga20ox1* and *ga20ox1 ga20ox3* displaying altered frequencies of abnormalities. These results suggest a novel, previously unidentified role for GA in floral patterning and early floral organ development, consistent with published expression patterns for GA biosynthetic and signalling genes.

## Materials and methods

### Plant material and growth conditions

The homozygous *ga20ox* mutant lines used in these experiments, comprising combinations of the *ga20ox1*-*3*, *ga20ox2*-*1* and *ga20ox3*-*1* alleles in the Col-0 background, have been previously described (Rieu et al. 2008; Plackett et al. 2012) and were compared against wild type (Col-0). Experiments characterising the frequency of silique-set during early flowering included *ga1*-*3*(Col-0) as a GA-deficient control (Tyler et al. 2004). All plants were grown on Levington F2 soil under long-day (LD) conditions (16-h light, 300 μmol m^−2^ s^−1^, 23 °C; 8-h darkness, 18 °C). Foliar treatments were applied to growing plants of each genotype three times a week from germination onwards as previously described (Rieu et al. 2008), comprising either mock treatment with water (control growth conditions) or 100 μM GA_3_ (GA treatment).

### Characterisation of silique-set across the early inflorescence

Plants of each genotype were allowed to develop to near-maturity, and then the presence or absence of a fertilised silique was scored across early flower positions on the primary inflorescence (unfertilised flower positions scored as ‘0’ and successful silique-set scored as ‘1’). The presence or absence of developing siliques was typically a binary character, with mature siliques clearly distinct from unfertilised pistils, and wild-type levels of seed-set per silique have previously been recorded for all single and double *ga20ox* mutants used in this analysis despite changes to silique length (Plackett et al. 2012). Where the presence of a single or a few seeds was suspected by distortion/bulging in apparently unpollinated pistils, and in cases of parthenocarpic pistil growth in response to GA treatment (Vivian-Smith and Koltunow 1999), these were checked manually by dissection. Silique-set was scored at each flower position between 1 and 10 (by which point, under our growth conditions, siliques were set reliably by all genotypes except *ga20ox1 ga20ox2 ga20ox3* and *ga1*-*3*) for each genotype under control growth conditions and under exogenous GA treatment (*n* = 12).

### Characterisation of floral organ lengths and floral organ abnormalities across the early inflorescence

Based on empirical evidence obtained from silique-set experiments, a range between flower positions 1–10 was selected for more detailed analysis of floral organ growth. Position 15 was also included as a representative of later flowering for comparison. These flower positions were sampled from wild-type and *ga20ox* mutant inflorescences under either control growth conditions or exogenous GA treatment (*n* = 4). Flowers were harvested from the primary inflorescence on the day of opening (floral stage 13; Smyth et al. 1990), taking a single flower per plant over the course of the experiment. In mutants where flower opening is not evident in the earliest flowers to develop (*ga20ox1 ga20ox2* and *ga20ox1 ga20ox2 ga20ox3*), the most advanced stage of development reached at each flower position was selected as a comparison. This was based on bud size, i.e. when the bud at the flower position specified had ceased to grow, and the following buds had attained a similar size, its development was adjudged to be complete. Upon harvesting, flowers were dissected and the number of floral organs in each whorl surveyed, noting the presence of any developmental abnormalities. Frequencies of deviations in floral organ number or organ developmental abnormalities were scored by type, with a value of 1 assigned for each individual instance observed. Dissected flowers were photographed, and the lengths of pistils, medial (long) stamens, petals and sepals were calculated from scaled images. Single measurements of each organ type were taken from each flower, measuring stamens, petals and sepals from where the organ joined the flower receptacle at its base to the organ tip. Pistils were measured from their base adjoining the receptacle to the lower edge of the stigma: a stamen length of 100% or greater relative to the pistil indicates that successful pollination is possible.

### Experimental design and statistical analysis

Characterisation experiments were performed in blocked split-plot experimental designs (Gomez and Gomez 1984). GA treatment was applied to main plots (whole soil trays) and genotype to split plots (cells within soil trays). Silique-set data were analysed using a generalised linear model fitting a logit link function (McCullagh and Nelder 1989) and assuming a binomial distribution. Pairwise comparisons of means in significant model terms (*p* < 0.05, Chi-squared test) were made using individual least significant difference (LSD) values at a significance threshold of 1% (Online Resource 1). Floral organ growth was analysed both through ANOVA, utilising data from all flower positions, and linear regression modelling, for data from positions 1–10. Where the distribution of data for individual floral organ types did not meet the assumptions of homogeneity of variance, analysis was performed on a transformed scale as required (ANOVA: square root for pistil and stamen data, Online Resource 2; linear regression: natural log for pistil and stamen data). For ANOVA, the standard error of the difference (SED) values on the relevant degrees of freedom was used to compare between genotypes at the same flower position, or between the different flower positions of one genotype within and between GA treatments, by way of the corresponding LSDs using a significance threshold of 5%. For linear regression modelling, estimated parameters (slopes and intercepts of fitted lines) are presented with calculated 95% confidence intervals (CIs). Comparisons of pairs of appropriate parameters between genotypes were made using *F* tests.

Observed frequencies of floral abnormalities were found to approximate to a Poisson distribution and were analysed as such, fitting a generalised linear model (GLM) with a square-root link function (McCullagh and Nelder 1989). In the absence of over-dispersion, the main effects and interactions between the factors genotype, GA treatment and flower position were assessed using changes in model deviance (*χ* ^2^ tests). The predicted frequencies of abnormalities from this model were compared between genotypes within GA treatments, between GA treatments within genotypes and between flower positions within GA treatments using individual LSDs with a significance threshold of 5% (Online Resource 3). The GenStat software package (2010, 13th edition; VSN International Ltd, Hemel Hempstead, UK) was used for all analyses.

## Results

### A trend of increasing silique-set with advancing flower position was identified, irrespective of genotype or exogenous GA treatment

As a proxy measure for floral organ development across early flower positions, the frequency of successful silique-set (indicating successful pollination, which necessarily requires stamen growth to have matched the pistil) during early flowering of the primary inflorescence was compared between genotypes under both control growth conditions and exogenous GA treatment. It was found that, under our growth conditions, siliques were reliably being set by flower position 10 (the first flower to develop on the inflorescence representing position 1) in all genotypes except the severely GA-deficient mutants *ga20ox1 ga20ox2 ga20ox3* and *ga1*-*3* (Online Resource 4). It should be noted that a previous analysis of *ga20ox1 ga20ox2* fertility, Rieu et al. (2008) found silique-set defects up to approximately flower position 15. We observed that there was no clear transition between early unpollinated flower positions and later successful silique-set, i.e. a flower position with a developed silique could be followed by an unpollinated position (Online resource 4). In response to the stochastic nature of this phenotype, we analysed the mean frequencies of silique-set per flower position from 12 individual primary inflorescences for each genotype between flower positions 1–10.

**Fig. 1:**
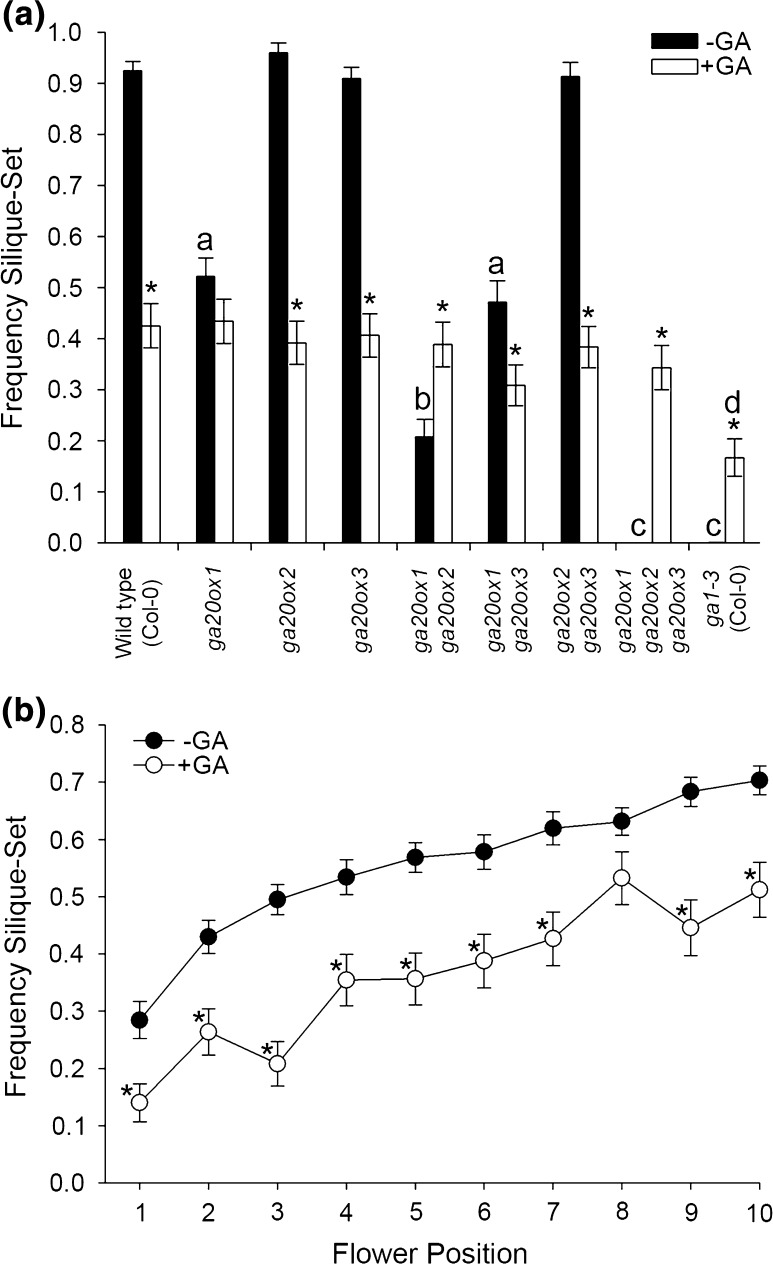
Probability of *Arabidopsis* silique-set is position-dependent across flower positions 1–10. Statistically significant interactions were detected between genotype and GA treatment (*p* < 0.001) and between flower position and GA treatment (*p* < 0.001). **a** Mean silique-set frequencies in wild-type and *ga20ox* mutant inflorescences averaged across flower positions 1–10, under control growth conditions (black) and exogenous GA treatment (white). **b** Mean silique-set frequencies for individual flower positions 1 through 10 averaged across all genotypes, under control growth conditions (black) and exogenous GA treatment (white). Values shown are the mean of 12 independent inflorescences ± S.E. Pairwise comparisons between genotypes, GA treatments and flower positions were made using LSD values at a 1% significance threshold (See Online Resource 1). Letters denote significant difference (*p* < 0.01) from wild type under control growth conditions (black) or GA treatment (grey). Genotypes marked with different letters are significantly different from each other. Asterisks denote a significant effect of GA treatment within a genotype (**a**) or flower position (**b**)

*GA20ox1*, *GA20ox2* and *GA20ox3* were found to act together in a partially redundant manner to maintain silique-set. Under control growth conditions, both the *ga20ox1 ga20ox2 ga20ox3* triple mutant and GA-deficient control (*ga1*-*3*) did not set siliques across positions 1–10 (Fig. 1a), as previously found (Plackett et al. 2012). Whilst there were further significant quantitative differences between wild type (Col-0) and some *ga20ox* mutants in the frequency of silique-set averaged across flower positions 1–10 (*ga20ox1* and *ga20ox1 ga20ox2*, with silique-set reduced in both genotypes; Fig. 1a, *p* < 0.01), there was no statistically significant interaction between genotype and flower position (*p* = 0.222), indicating that the pattern of silique-set across the early inflorescence was similar for all genotypes. In agreement with previous analyses of *ga20ox* mutant phenotypes (Rieu et al. 2008), under our experimental conditions mean silique-set was reduced more severely in *ga20ox1 ga20ox2* than in *ga20ox1* (*p* < 0.01). The *ga20ox1 ga20ox3* and *ga20ox2 ga20ox3* double mutants were found to phenocopy *ga20ox1* and *ga20ox2*, respectively (*p* > 0.01; Fig. 1a; with *ga20ox1 ga20ox3* also showing a significant reduction in fertility compared to wild type, *p* < 0.01), suggesting that *GA20ox3* acts redundantly with *GA20ox1* and *GA20ox2* to promote successful silique-set. This same hierarchical relationship is also found across other plant developmental processes (Plackett et al. 2012).

GA treatment enhanced silique-set in the most severely GA-deficient genotypes (*ga20ox1 ga20ox2*, *ga20ox1 ga20ox2 ga20ox3* and *ga1*-*3*) (*p* < 0.01; Fig. 1a), but reduced it in all other genotypes (including wild type) except *ga20ox1* (*p* < 0.01). Under GA treatment, mean silique-set became similar between all genotypes except *ga1*-*3* (*p* > 0.01; Fig. 1a). The observed negative effects of GA treatment on wild-type silique-set are consistent with previous studies (Rieu et al. 2008; Plackett et al. 2014), and the similarity in silique-set between most GA-treated genotypes indicates that GA responses were saturated under our exogenous treatment.

Irrespective of genotype, advancing flower position was found to have a significant positive effect on the probability of successful silique-set both under control growth conditions and under exogenous GA treatment (Fig. 1b). In both cases, the mean frequency of silique-set at flower 10 was significantly different compared to position 1 (*p* < 0.01). GA treatment reduced silique-set at all flower positions studied to a significant degree (*p* < 0.01) except position eight. This trend suggests that an additional mechanism is acting independently of GA signalling to increase the probability of silique-set as flowering progresses. Given the dependence of pollination on coordinated floral organ growth, it was considered likely that this mechanism acts through differential changes to floral organ growth across early flowering.

### Changes in floral organ growth are sufficient to explain the recovery of silique-set in GA-deficient inflorescences

**Fig. 2:**
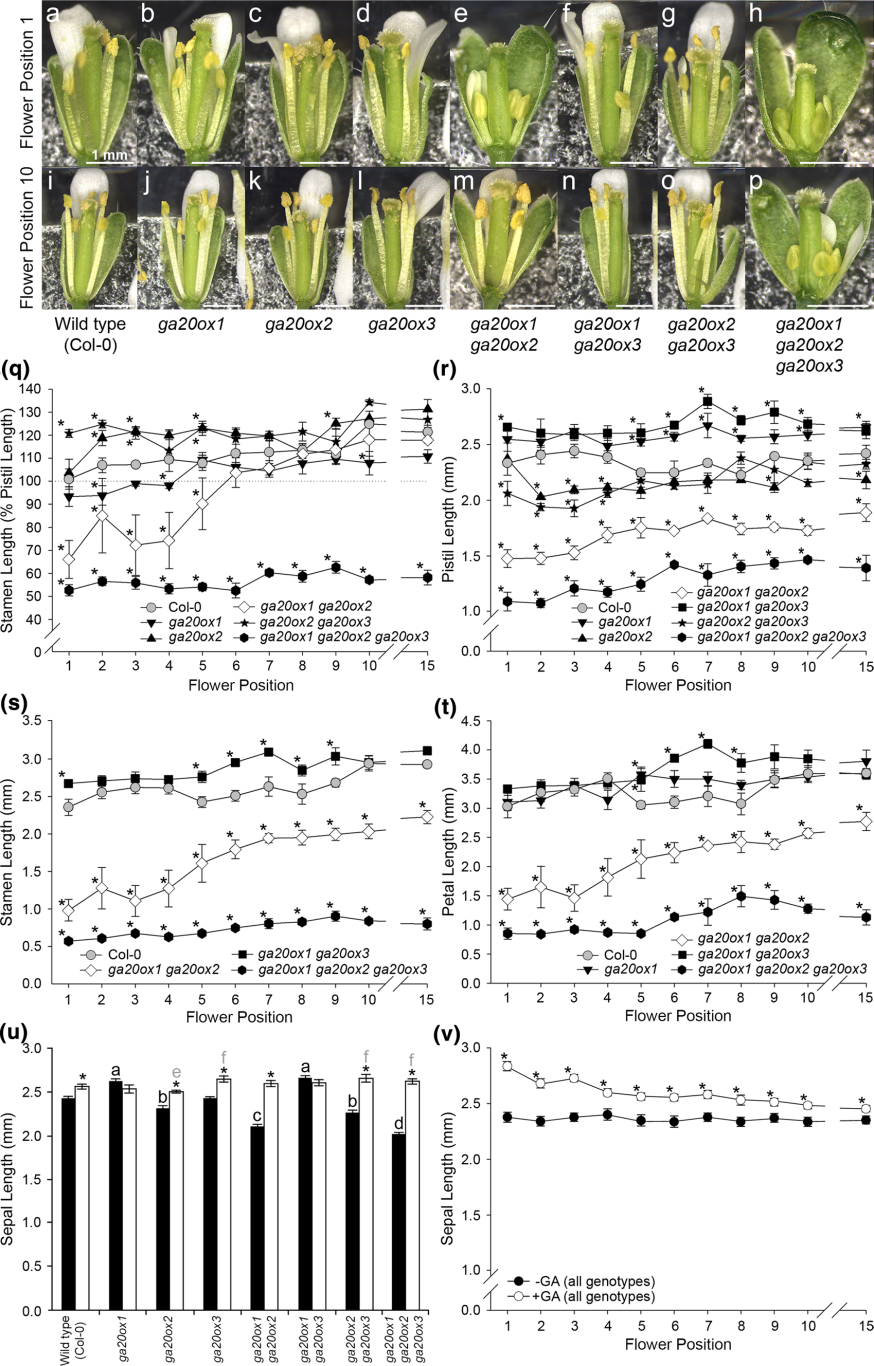
*Arabidopsis* floral organ lengths change in a position-dependent manner across flower positions 1–10. Floral phenotypes of wild type (Col-0) and *ga20ox* mutants, as specified, at flower positions one (**a**–**h**) and ten (**i**–**p**) under control growth conditions. All flowers shown are newly opened (floral stage 13; Smyth et al. 1990). Statistically significant three-way interactions were found between genotype, flower position and GA treatment for relative stamen length (*p* < 0.001) and absolute lengths of pistils (*p* < 0.001), stamens (*p* < 0.001) and petals (*p* = 0.002) across the early inflorescence. For sepals, a three-way interaction was not statistically significant (*p* = 0.275), but three separate significant two-way interactions were identified (genotype by GA, *p* < 0.001; genotype by flower position, *p* = 0.018; GA by flower position, *p* < 0.001). Mean floral organ lengths of newly opened (stage 13) flowers across flower positions 1–10 and position 15, showing stamen length as a percentage of pistil length (**q**) and absolute lengths (in mm) of pistils (**r**), stamens (**s**), petals (**t**). Flower position 15 is included as an indicator of floral organ growth in later flowering. Values shown are the mean of four independent flowers ± S.E. Genotypes not represented on individual graphs were not significantly different from wild type at any flower position (*p* > 0.05). Significant differences (*p* < 0.05) from wild type within each flower position are denoted by asterisks. Mean sepal lengths of stage 13 flowers, for each genotype under control growth conditions (black) and exogenous GA treatment (white) averaged across all flower positions measured (**u**) and for each flower position under control growth conditions (black) and exogenous GA treatment (white), averaged across all genotypes (**v**). Values shown are the mean of 44 (**u**) and 32 independent flowers (**v**) ± S.E. Letters denote significant difference (*p* < 0.01) from wild type under control growth conditions (black) or GA treatment (grey). Genotypes marked with different letters are significantly different from each other. Asterisks denote a significant effect of GA treatment within a genotype (**u**) or flower position (**v**). Pairwise comparisons in (**q**–**v**) were made using LSDs at a significance threshold of 5%, with pistil and stamen lengths being analysed on the square-root scale (see Online Resource 2)

To determine the contribution of floral organ growth to the changes in silique-set described above, we analysed *ga20ox* floral phenotypes (synchronised at flower opening, see “Materials and methods”) between flower positions 1–10 under both control growth conditions and GA treatment. Comparing the phenotypes of the first (Fig. 2a–h) and tenth flowers (Fig. 2i–p) of each genotype showed that the greatest phenotypic changes across the early inflorescence occurred in *ga20ox1 ga20ox2*: in flower 1 *ga20ox1 ga20ox2* stamens and petals were dramatically reduced compared to wild type (Fig. 2a, e) but were similar to wild type in flower 10 (Fig. 2i, j). The anthers in *ga20ox1 ga20ox2* flower 1 were indehiscent (Fig. 2e), whereas wild-type anthers had already released pollen by the time flower 1 opened. A similar phenotype of underdeveloped floral organs in early *ga20ox1 ga20ox2* flowers was reported by Rieu et al. (2008). By the opening of flower 10 *ga20ox1 ga20ox2* anthers had successfully dehisced, with pollen visible on the stigma (Fig. 2m). In contrast, *ga20ox1 ga20ox2 ga20ox3* floral organs remained reduced and anthers indehiscent up to flower 10 (Fig. 2h, p). The floral phenotypes of all other genotypes were found to superficially resemble wild type. Interestingly, in most genotypes including wild type we observed that at flower opening stamens in flower 10 appeared longer relative to the pistil compared to flower 1 (Fig. 2a, i), suggesting that relative floral organ growth changes across early flowering. Spontaneous recovery of the *ga20ox1 ga20ox2* floral phenotype was thus confirmed over our selected experimental range. We also confirmed that under our experimental conditions exogenous GA treatment rescued the floral phenotypes of both *ga20ox1 ga20ox2* and *ga20ox1 ga20ox2 ga20ox3* to resemble that of wild type (Online Resource 4).

The hypothesis that changes in floral organ growth can explain the observed changes in silique-set was tested quantitatively through direct measurement of floral organs across the early inflorescence (Fig. 2q–v), comparing between genotypes at each flower position. For all genotypes, some stochastic variation in mean floral organ length was evident between adjacent flower positions (i.e. mean length could be lower or higher at a subsequent flower position), consistent with the observed variations in silique-set across the same range of flower positions.

In wild-type flowers, under control conditions mean stamen length was ≥ 100% of pistil length at flower opening at every flower position examined (Fig. 2q). Consistent with their silique-set phenotypes, a number of early flowers in both *ga20ox1* and *ga20ox1 ga20ox2* contained stamens whose relative length was significantly different from wild type and less than 100% of their respective pistils (Fig. 2q, *p* < 0.05). Congruent with their relative phenotypic severity, fewer flowers were significantly different between wild type and *ga20ox1* (flowers 2 and 4) than *ga20ox1 ga20ox2* (flowers 1–5), and at most of these earliest flower positions (flowers 1, 3, 4, 5) *ga20ox1 ga20ox2* relative stamen lengths were significantly reduced compared to *ga20ox1* (*p* < 0.05; Online Resource 2). At later flower positions (beyond flower 5), stamen length in these two genotypes was not significantly different from wild type (*p* > 0.05) and consistently ≥ 100% of the pistil. In contrast to this, two other genotypes (*ga20ox2* and *ga20ox2 ga20ox3*) surprisingly showed significant differences where relative stamen length was in fact greater than in wild type (*p* < 0.05; Fig. 2q). In *ga20ox1 ga20ox2 ga20ox3*, relative stamen lengths were significantly different from both wild type and *ga20ox1 ga20ox2* at all flower positions examined (*p* < 0.05; Online Resource 2) and remained below 100% of pistil length throughout. Under GA treatment, relative stamen growth was rescued to ≥ 100% of the pistil in almost all genotypes and flower positions (Online Resource 4), indicating that the phenotypes observed are a result of impaired GA biosynthesis. These results suggest that the differences in mean silique-set observed between wild type and some GA-deficient mutants during early flowering (Fig. 1a) can mostly be explained through GA-dependent changes in stamen growth relative to the pistil in the earliest flowers to open, with all three *GA20ox* paralogues contributing.

Growth of all floral organs (as measured by absolute length at flower opening) was altered by GA deficiency, with loss of different paralogues having complex phenotypic effects. Consistent with the long-established role for GA in promoting *Arabidopsis* floral organ growth (Koornneef and Van der Veen 1980), *ga20ox1 ga20ox2 ga20ox3* pistils, stamens and petals all exhibited significantly reduced growth compared to wild type at all flower positions (*p* < 0.05; Fig. 2r–t), and mean sepal length across the early inflorescence was similarly reduced (*p* < 0.05; Fig. 2u). Whilst the absolute length of all floral organs was also reduced in *ga20ox1 ga20ox2* across the early inflorescence (*p* < 0.05; Fig. 2r–u), floral organ growth was less severely reduced than the triple mutant phenotype (*p* < 0.05; Online Resource 2). In contrast to these two mutants, *ga20ox1* and *ga20ox1 ga20ox3* frequently demonstrated increased floral organ growth compared to wild type, with significantly different lengths in pistils (*p* < 0.05; Fig. 2r), stamens (*ga20ox1 ga20ox3* only) (*p* < 0.05; Fig. 2s), petals (*p* < 0.05; Fig. 2t) and sepals (*p* < 0.05; Fig. 2u). Loss of *GA20ox2* alone, or in combination with *GA20ox3*, significantly affected pistil and sepal lengths (*p* < 0.05; Fig. 2r, u), but not those of stamens or petals (*p* > 0.05). The relationship between *GA20ox* paralogues and growth of different floral organs across the early inflorescence was thus found to be complex, pointing to functional differences relating to their sites of expression. In particular, sepal responses appeared distinct from those of inner floral organs. These differences were also observed in flower 15 (Fig. 2r–t, v), suggesting that they are not limited to early flowering.

Importantly, *ga20ox1 ga20ox2* floral organ lengths across the early inflorescence under control growth conditions show a clear trend of increasing stamen and petal length with advancing flower position (Fig. 2s, t). A similar trend might similarly be indicated in *ga20ox1 ga20ox2* pistils (Fig. 2r) but with a lesser magnitude than seen in stamens. This observation suggests the observed recovery of relative stamen growth in *ga20ox1 ga20ox2* might be explained by differentially increased growth of the stamen, whilst pistil growth remains relatively static. Our observations at the whole flower level (Fig. 2a–p) and significant increases in mean floral organ lengths between flowers 1 and 10 in other genotypes, including in wild type (*p* < 0.05 for stamens and petals; Online Resource 2), suggest that changes in floral organ growth are a normal component of wild-type flowering.

**Fig. 3:**
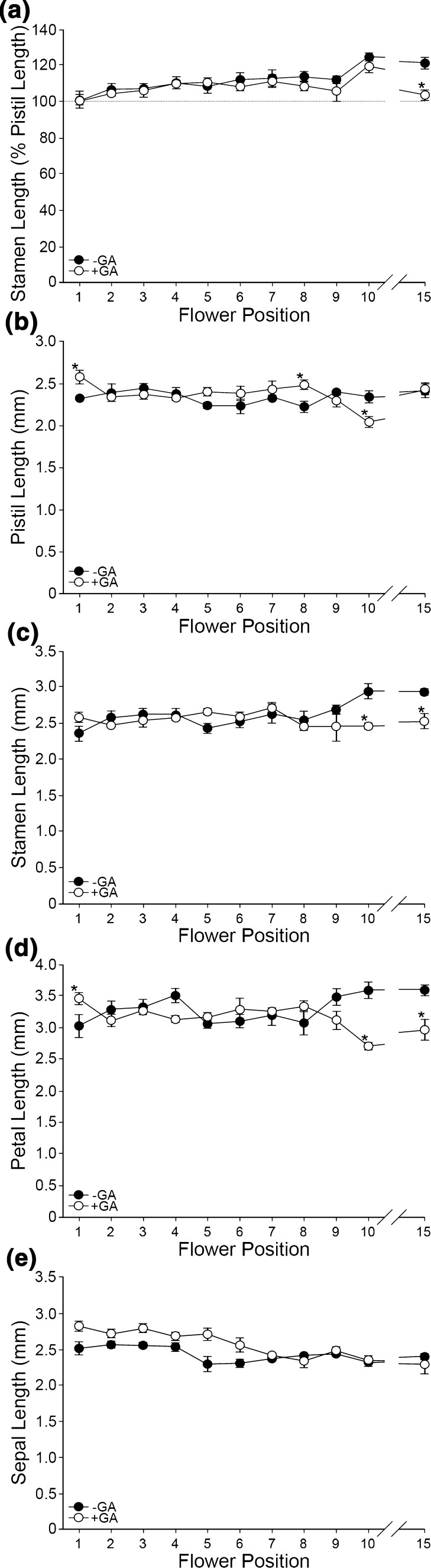
Effect of exogenous GA treatment on floral organ lengths of wild-type (Col-0) flowers during early flowering. Mean floral organ lengths of newly opened (stage 13) wild-type (Col-0) flowers across flower positions 1–10 and position 15 under control growth conditions (−GA, black) and exogenous GA treatment (+GA, white), showing stamen length as a percentage of pistil length (**a**) and absolute lengths (in mm) of pistils (**b**), stamens (**c**), petals (**d**) and sepals (**e**). Flower position 15 is included as an indicator of floral organ growth in later flowering. Values shown are the mean of four independent flowers ± S.E. Significant differences (*p* < 0.05) between control growth conditions and GA treatment within each flower position are denoted by asterisks. Statistical analyses of stamens and petals were performed on a transformed scale (see “Materials and methods” and Online Resource 4). Statistical comparison of sepal lengths was not valid in the absence of a significant interaction between genotype, GA treatment and flower position (see Fig. 2)

GA treatment of *ga20ox* mutants rescued mean pistil, stamen and petal lengths to values similar to wild type at almost all organ, genotype and flower position combinations (Online Resource 4). Growth of wild-type pistils, stamens and petals was mostly unaffected by GA treatment across the early inflorescence (Fig. 3a–d). This is in contrast to our findings regarding silique-set, where under GA treatment successful wild-type silique-set was reduced to approximately 50% of that under control growth conditions (Fig. 1a). However, mean sepal length was significantly increased by GA treatment in all genotypes (including wild type) across the early inflorescence with the exception of *ga20ox1* and *ga20ox1 ga20ox3* (*p* > 0.05; Fig. 2u), in which sepal length was already significantly increased relative to wild type under control growth conditions (*p* < 0.05; Fig. 2u). Sepal length was also significantly increased under GA treatment at every flower position when averaged across all genotypes (*p* < 0.05; Fig. 2v). We thus identified a differential response to GA between sepals and other floral organs. The observation that silique-set is reduced under exogenous GA treatment although stamen length being ≥ 100% of the pistil is consistent with previous observations of reduced fertility under GA treatment (Jacobsen and Olszewski 1993) and in constitutive GA signalling DELLA loss-of-function mutants (Dill and Sun 2001; Plackett et al. 2014), where GA-dependent defects in pollen development were identified as the cause.

### Floral organs display gradients of growth across the early inflorescence

Linear regression modelling across flower positions 1–10 (see “Materials and methods”) was used to test for the existence of significant changes in floral organ growth between different flowers of the early inflorescence. Growth of each floral organ type across the early inflorescence was thus described using two parameters: the intercept (a theoretical starting organ length at flower position 0) and the gradient (the change in floral organ length between two successive flower positions) (Table 1). Under control growth conditions, we identified a significant positive gradient in stamen growth across the early wild-type inflorescence (*p* < 0.05; Table 1a), whereas the corresponding gradients for wild-type pistils and petals were not significantly different from 0 (*p* > 0.05). In contrast, a significant negative gradient growth was detected for sepals (*p* < 0.05). These results demonstrate the existence of previously unrecognised gradients in the growth of floral organs across the early inflorescence, which vary between different organ types. Differences in the gradient in growth between stamens and pistils can thus explain the positive trend in silique-set observed across the early inflorescence (Fig. 1b). No significant difference in the length of wild-type stamens was detected between flower positions 10 and 15 (*p* > 0.05, Online Resource 2), suggesting that this gradient could be limited to (or is at least strongest during) early flowering.

**Table 1:** Linear regression modelling of floral organ lengths across the early *Arabidopsis* inflorescence

(a)								
Genotype	Ln(Pistil) intercept (−GA)	Ln(Pistil) gradient (−GA)	Ln(Stamen) intercept (−GA)	Ln(Stamen) gradient (−GA)	Petal intercept (−GA)	Petal gradient (−GA)	Sepal intercept (−GA)	Sepal gradient (−GA)
Wild type (Col-0)	[0.8639]	[− 0.0030]	[0.8730]	[0.0133]	3.1144	0.0275	2.5604	− 0.02445
*ga20ox1*	[0.9282]^a^	[0.0024]	[0.8546]	[0.0204]	3.1296	0.0452	2.7309^a^	− 0.01884
*ga20ox2*	[0.7523]^b^	[0.0034]	[0.8590]	[0.0172]	3.0995	0.0300	2.2452^b^	0.01101^a^
*ga20ox3*	[0.8589]	[− 0.0064]	[0.9044]	[0.0097]	3.1680	0.0213	2.4997	− 0.01264
*ga20ox1 ga20ox2*	[0.3903]^c^	[0.0216]^a^	[− 0.0945]^a^	[0.0914]^a^	1.3181^a^	0.1325^a^	2.0323^c^	0.01216^a^
*ga20ox1 ga20ox3*	[0.9450]	[0.0071]^b^	[0.9670]^b^	[0.0141]	3.2349	0.0753^b^	2.6641^a^	− 0.00051^a^
*ga20ox2 ga20ox3*	[0.6606]^b^	[0.0162]^a^	[0.8296]	[0.0202]	2.9798	0.0527	2.1825^b^	0.01287^a^
*ga20ox1 ga20ox2 ga20ox3*	[0.0436]^d^	[0.0362]^c^	[− 0.6024]^c^	[0.0487]^b^	0.6830^b^	0.0737^b^	2.0175^d^	− 0.00105
± for 95% C.I.	[0.0481]	[0.0078]	[0.0672]	[0.0108]	0.1942	0.0312	0.0870	0.01298

(b)								
Genotype	Ln(Pistil) intercept (+GA)	Ln(Pistil) gradient (+GA)	Ln(Stamen) intercept (+GA)	Ln(Stamen) gradient (+GA)	Petal intercept (+GA)	Petal gradient (+GA)	Sepal intercept (+GA)	Sepal gradient (+GA)
Wild type (Col-0)	[0.9233]	[− 0.0111]	[0.9440]	[− 0.0005]	3.3775	− 0.0362*	2.8789*	− 0.05398*
*ga20ox1*	[0.9070]	[− 0.0099]*	[0.8853]	[0.0044]*	3.1862	− 0.0358*	2.7967*	− 0.04837*
*ga20ox2*	[0.8356]^a^*	[− 0.0034]	[0.9107]*	[0.0029]	3.1416	− 0.0049	2.6190^a^*	− 0.01852^a^*
*ga20ox3*	[0.8380]	[0.0045]^a^	[0.9210]	[0.0052]	3.2390	− 0.0017	2.8923*	− 0.04217*
*ga20ox1 ga20ox2*	[0.8460]*	[− 0.0015]*	[0.8396]^a^*	[0.0091]*	3.1806*	0.0064^a^*	2.6933^a^*	− 0.01737^a^*
*ga20ox1 ga20ox3*	[0.9196]	[− 0.0038]	[0.9019]	[0.0100]	3.2915	− 0.0040*	2.7725*	− 0.03004^b^*
*ga20ox2 ga20ox3*	[0.8875]*	[− 0.0008]*	[0.8852]	[0.0095]	3.2821	− 0.0051*	2.7590*	− 0.01666^ab^*
*ga20ox1 ga20ox2 ga20ox3*	[0.9033]*	[− 0.0093]*	[0.8711]^a^*	[0.0021]*	3.1326^a^*	− 0.0118*	2.7891*	− 0.03058^b^*
± for 95% C.I.	[0.0481]	[0.0078]	[0.0672]	[0.0108]	0.1942	0.0312	0.0870	0.01300

Linear regression modelling of floral organ lengths in stage 13 *Arabidopsis* flowers across flower positions 1–10 under control growth conditions (a) and exogenous GA treatment (b) (see “Materials and methods”). The linear models fitted accounted for a significant proportion of variance (*p* < 0.001): *R* ^2^ = 86% (pistils), 92% (stamens), 81.7% (petals) and 63.6% (sepals) of variance, respectively. Values shown are estimated intercepts (organ length in mm at theoretical flower position 0) and gradients (change in organ length in mm with flower position) given four independent flowers per genotype per flower position, and the ± adjustment for 95% confidence intervals (CI). Where analysis was performed on transformed data to fit statistical assumptions (see “Materials and methods”), these values are given in square brackets. Superscript letters denote significant difference from the wild type (*p* < 0.05). Genotypes marked with different superscript letters are significantly different from one another. Asterisks indicate a significant effect of GA treatment (*p* < 0.05) compared to control growth conditions

Under control growth conditions, a number of *ga20ox* mutants demonstrated altered floral organ growth parameters across the early inflorescence compared to wild type. All *ga20ox* mutants except *ga20ox3* retained a significant positive gradient for stamen growth (significantly different from 0; *p* < 0.05, Table 1a). In the *ga20ox1* mutant, where relative stamen growth is reduced in the earliest flowers (Fig. 2q), stamen growth parameters were similar to wild type (*p* > 0.05), but initial pistil length (as described by the intercept) was significantly greater than in wild type (*p* < 0.05). In contrast, the *ga20ox* pistil gradient was not significantly different from wild type (*p* > 0.05). In consequence, reduced relative stamen growth in this genotype does not relate to changes in stamen growth but is instead the outcome of increased pistil growth throughout the early inflorescence, delaying the flower position in which stamen growth becomes sufficient to ensure pollination. Conversely in *ga20ox2*, which demonstrated increased relative stamen growth in some early flowers (Fig. 2q), the pistil intercept was reduced compared to wild type (*p* < 0.05), whilst the pistil gradient and stamen parameters were not significantly different from wild type (*p* > 0.05). Loss of *GA20ox3* in either the *ga20ox1* or *ga20ox2* backgrounds caused further significant changes in growth relationships (*p* < 0.05; Table 1a), suggesting that these paralogues are not fully redundant. Modelling growth relationships at the level of the inflorescence rather than individual flowers has thus provided a better description of the processes underlying changes in relative organ growth, and through this a clearer understanding of *GA20ox* paralogue function.

In the *ga20ox1 ga20ox2* background, pistil, stamen and petal growth parameters were all significantly different from those of wild-type, *ga20ox1* and *ga20ox2* (*p* < 0.05; Table 1a). Growth parameters for these organs also differed between *ga20ox1 ga20ox2* (*p* < 0.05) and *ga20ox1 ga20ox2 ga20ox3*. In these two more severely GA-deficient genotypes, in addition to the stamen gradient, pistil and petal gradients also became significantly positive (*p* < 0.05). Sepal growth was also positive in *ga20ox1 ga20ox2* (*p* < 0.05), but not in the triple mutant (*p* > 0.05). In conjunction with these positive gradients, floral organ intercept values were all reduced in these two mutants (*p* < 0.05), reflecting shorter organs in the first flowers to open followed by an increased rate of organ growth with advancing flower position compared to wild type. These results suggest that the positive gradient in stamen growth seen under control growth conditions could be independent of GA biosynthesis and that GA-dependent floral organ growth is overlaid onto other, independent gradients.

Under GA treatment, the growth relationships of floral organs in *ga20ox* mutants were mostly rescued to that of wild type (Table 1b). In this analysis, the wild-type inflorescence did show some response to GA treatment, with significant changes in the growth gradients of petals and sepals (*p* < 0.05), and a significant increase in the sepal intercept (*p* < 0.05). Whilst there was no formal significant difference in wild-type stamens and pistils between control growth conditions and under GA treatment, the stamen gradient under GA treatment was found to be no longer significantly different from 0. In all GA-treated genotypes, the gradients of each floral organ type (including stamens) were either not significantly different from 0 (*p* > 0.05) or negative (*p* < 0.05) (Table 1b). This suggests that floral organ growth responses to GA are not fully saturated in early flowers. Changes in GA biosynthesis or signalling may therefore play a role in the early wild-type inflorescence to generate organ-specific growth gradients.

### The recovery of *ga20ox1 ga20ox2* stamen development can be explained through a discontinuous phenotype independent of gradients in organ growth

**Fig. 4:**
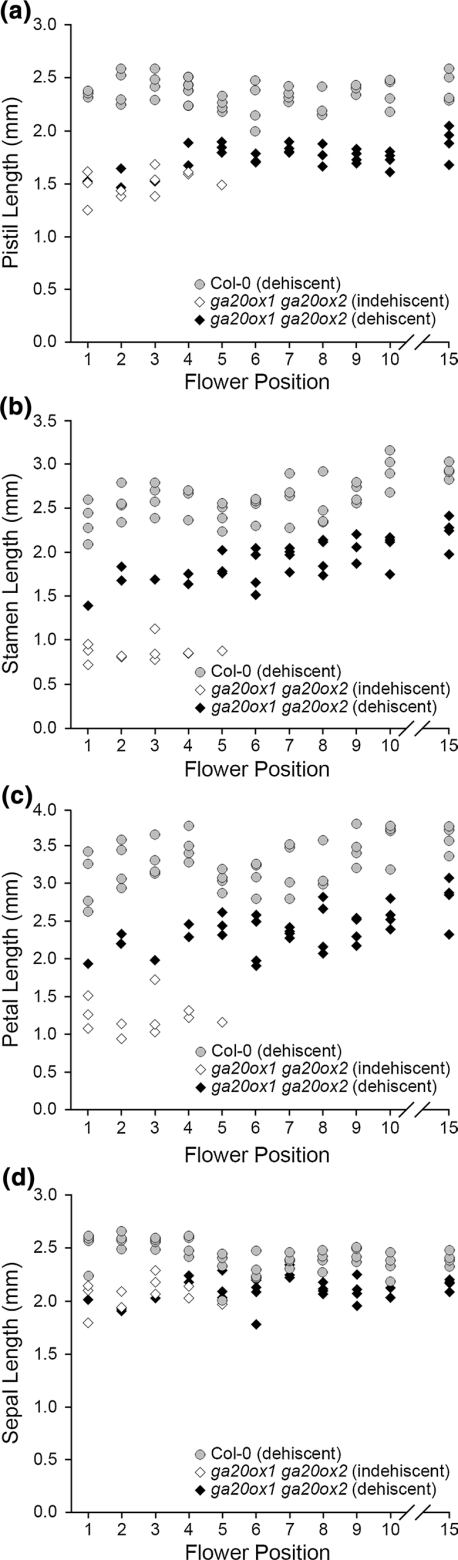
*ga20ox1 ga20ox2* stamen and petal lengths are reduced in flowers with indehiscent anthers. Distribution of organ lengths for pistils (**a**), stamens (**b**), petals (**c**) and sepals (**d**) from *ga20ox1 ga20ox2* flowers across the early inflorescence, distinguishing flowers where anthers were indehiscent (white) or dehiscent (black) at flower opening. Wild-type floral organ lengths (in which all flowers were dehiscent) are shown in grey for comparison

Stamen length relative to the pistil in the *ga20ox1 ga20ox2* mutant was found to be far more variable than in other genotypes in the earliest flowers (flower positions 1–5; Fig. 2q). This was caused by variation in the absolute lengths of stamens, but not of pistils (Fig. 2r, s). It was noted that stamens of *ga20ox1 ga20ox2* flowers were also frequently indehiscent in this region of the early inflorescence, but not beyond. To test for a relationship between these two factors, the lengths of floral organs from *ga20ox1 ga20ox2* flowers with indehiscent or dehiscent anthers were plotted separately (Fig. 4). Whilst *ga20ox1 ga20ox2* pistils were of similar lengths between flowers with dehiscent and indehiscent anthers (Fig. 4a), stamens with indehiscent anthers were clearly further reduced in length (Fig. 4b). *ga20ox1 ga20ox2* flowers with indehiscent anthers also showed a reduction in petal length (Fig. 4c), but not sepal length (Fig. 4d). Failure in anther dehiscence in early *ga20ox1 ga20ox2* flowers thus appears to be specifically associated with reduced growth of particular floral organs (stamen and petals) and not others (pistils and sepals).

To determine whether reduced stamen and petal growth in flowers with indehiscent anthers were sufficient to explain the significant differences in growth parameters between *ga20ox1 ga20ox2* and wild-type inflorescences (Table 1a), linear regression analysis was repeated for stamens and petals excluding measurements from indehiscent flowers (Table 2). With indehiscent organ lengths excluded, the difference in the gradient between *ga20ox1 ga20ox2* and wild-type stamens became much less significant (*p* < 0.001 to *p* = 0.036), and the gradient of petal growth became similar (*p* = 0.262). Both gradient values remained significantly different from 0 (*p* < 0.05, Table 2). The *ga20ox1 ga20ox2* stamen and petal intercept values remained significantly different from wild type (*p* < 0.001 for each), reflecting reduced organ growth at all flower positions. Much of the dramatic ‘recovery’ phenotype seen across the early *ga20ox1 ga20ox2* inflorescence can therefore be explained by a separable phenotype in which stamen and petal growth are restricted in flowers exhibiting indehiscent anthers. Underlying this, an independent gradient of increasing stamen and petal length with advancing flower position remains.

**Table 2:** Excluding indehiscent floral organs from *ga20ox1 ga20ox2* results in growth relationships similar to wild type

Genotype	Ln(Stamen) intercept (−GA)	Ln(Stamen) gradient (−GA)	Ln(Stamen) intercept (+GA)	Ln(Stamen) gradient (+GA)	Ln(Petal) intercept (−GA)	Ln(Petal) gradient (−GA)	Ln(Petal) intercept (+GA)	Ln(Petal) gradient (+GA)
Wild type (Col-0)	[0.8730]	[0.0133]	[0.9440]	[− 0.0005]	[1.1331]	[0.0083]	[1.2191]	[− 0.0120]
*ga20ox1*	[0.8546]	[0.0204]	[0.8853]	[0.0044]	[1.1379]	[0.0138]	[1.1565]	[− 0.0120]
*ga20ox2*	[0.8590]	[0.0172]	[0.9107]	[0.0029]	[1.1309]	[0.0089]	[1.1423]	[− 0.0015]
*ga20ox3*	[0.9044]	[0.0097]	[0.9210]	[0.0052]	[1.1469]	[0.0067]	[1.1727]	[− 0.0007]
*ga20ox1 ga20ox2*	**[0.4345]** ^a^	**[0.0285]** ^a^	**[0.8396]** ^a^	**[0.0091]**	**[0.7316]** ^a^	**[0.0184]**	**[1.1509]**	**[0.0025]** ^a^
*ga20ox1 ga20ox3*	[0.9670]	[0.0141]	[0.9019]	[0.0100]	[1.1770]	[0.0206]	[1.1849]	[− 0.0009]
*ga20ox2 ga20ox3*	[0.8296]	[0.0202]	[0.8852]	[0.0095]	[1.0914]	[0.0159]	[1.1864]	[− 0.0018]
*ga20ox1 ga20ox2 ga20ox3*	[− 0.6024]^b^	[0.0487]^b^	[0.8711]^a^	[0.0021]	[− 0.3146]^b^	[0.0658]^a^	[1.1364]^a^	[− 0.0036]
± for 95% CI	[0.0528]	[0.0085]	[0.0528]	[0.0085]	[0.0660]	[0.0106]	[0.0660]	[0.0106]

Re-analysis of linear regression modelling of stamen and petal lengths in newly opened *Arabidopsis* flowers across flower positions 1–10 under control growth conditions (see “Materials and methods”), excluding values from *ga20ox1 ga20ox2* flowers where stamens were indehiscent at flower opening (Fig. 4b, c). Removal of data from indehiscent flowers marginally increased the explanatory power of both models: *R* ^2^ = 93% (stamens) and 85.8% (petals) of variance, respectively. Values shown are estimated intercepts (organ length in mm at theoretical flower position 0) and gradients (change in organ length in mm with flower position) and the ± adjustment for 95% confidence intervals (CI). Parameter estimates for *ga20ox1 ga20ox2* are highlighted in bold. The analysis was performed on transformed data to fit statistical assumptions (see “Materials and methods”) so the values are given in square brackets. Superscript letters denote significant difference from the wild type (*p* < 0.05). Genotypes marked with different superscript letters are significantly different from one another

### Floral patterning is not stereotypical in the first flowers to develop

**Fig. 5:**
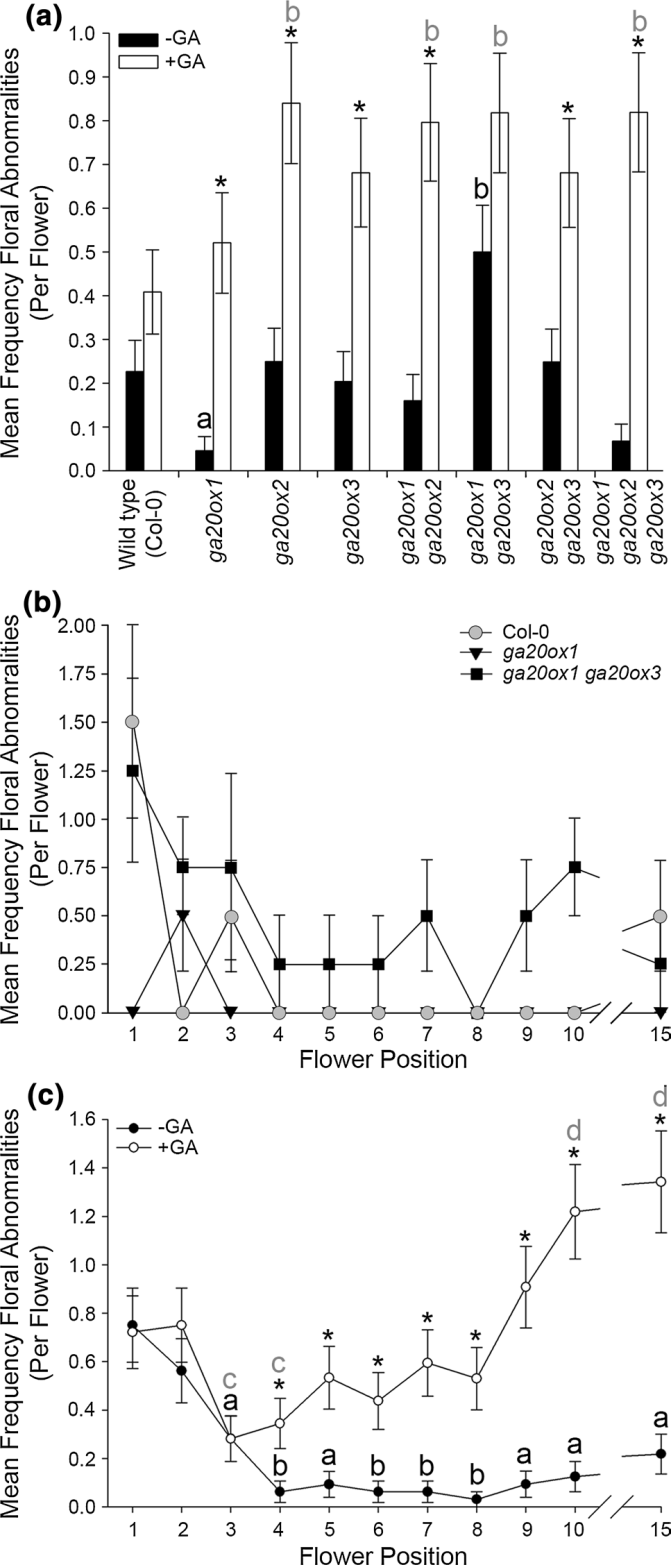
Early floral developmental events are significantly affected by both GA signalling and flower position. The incidence of floral abnormalities was analysed by GLM (see “Materials and methods”). Statistically significant two-way interactions were detected between genotype and GA treatment (*p* = 0.024), genotype and flower position (*p* = 0.033) and between GA treatment and flower position (*p* < 0.001). There was no significant three-way interaction (*p* = 0.807). **a** Mean frequencies of floral abnormalities (collective deviations in expected floral organ number, organ fusion and organ homeosis) in *ga20ox* mutants averaged across the early inflorescence (flowers 1–10), under control (black) and GA-treated conditions (white). Values shown are the mean of 44 independent flowers ± S.E. Asterisks indicate a significant difference between control and GA-treated conditions (*p* < 0.05) within a genotype. Letters indicate significant difference (*p* < 0.05) of genotypes compared to wild type under either control growth conditions (black letters) or GA treatment (grey letters). Genotypes or flower positions denoted by different letters are significantly different from one another (*p* < 0.05). **b** Plotted mean frequencies of floral abnormalities at flower positions 1–10 and 15 under control growth conditions, comparing wild type to the *ga20ox1* and *ga20ox1 ga20ox3* mutants which showed significant differences in (**a**). Values shown are the mean of four independent flowers ± S.E. **c** Mean frequencies of floral abnormalities at flower positions 1–10 and 15 averaged across all genotypes, under control (black) and GA-treated conditions (white). Values shown are the mean of 32 independent flowers ± S.E. Asterisks indicate a significant difference between control and GA-treated conditions (*p* < 0.05) within a single flower position. Letters indicate significant difference (*p* < 0.05) of flower positions compared to position one under either control growth conditions (black letters) or GA treatment (grey letters). Flower positions denoted by different letters are significantly different from one another (*p* < 0.05) within the same growth condition. No comparison was made between different flower positions from separate growth conditions. Pairwise comparisons in (**a**, **c**) were made using LSDs with a significance threshold of 5% (see Online Resource 3)

Although *Arabidopsis* floral development is reported as highly stereotypical (Smyth et al. 1990), we frequently observed deviations from this pattern, with abnormal numbers of floral organs present and/or developmental defects in individual organs. These abnormalities were found in all genotypes including wild type (Fig. 5a). Two mutants, *ga20ox1* and *ga20ox1 ga20ox3*, demonstrated mean frequencies of abnormalities significantly different from wild type (*p* < 0.05; Fig. 5a). Surprisingly, these mutants demonstrated apparently opposing phenotypes with a reduced and increased frequency of abnormalities, respectively. In contrast, the two most GA-deficient mutants, *ga20ox1 ga20ox2* and *ga20ox1 ga20ox2 ga20ox3*, did not demonstrate significant differences from wild type (*p* > 0.05). When plotted by flower position, the mean frequency of abnormalities in wild type under control growth conditions was greatest across flowers 1–3 before dropping away (Fig. 5b). Abnormalities in *ga20ox1* were reduced in this region, but whereas *ga20ox1 ga20ox3* displayed a similar frequency of abnormalities to wild type in the earliest flowers, they continued to appear in later flowers to develop. Under control growth conditions, floral abnormalities were most frequent in the first flowers to develop in all genotypes, with the mean frequency across all genotypes highest in flower 1 and then reducing significantly by flowers 3–4 (*p* < 0.05; Fig. 5c). The frequency of abnormalities in these first flowers was not affected by GA treatment, but were significantly more frequent than under control growth conditions by flower position 4 (*p* < 0.05) and they became increasingly frequent as flowering progressed.

It thus appears that, contrary to previous assumptions, *Arabidopsis* floral patterning is not uniformly stereotypical across the inflorescence, with the first flowers to develop showing significant deviations and patterning not becoming fully constrained until flower four. Differences in phenotype between *ga20ox1* and *ga20ox1 ga20ox3* suggest a possible role for GA, strongly supported by the GA treatment results. The maintenance of developmental abnormalities into later flowering under GA treatment suggests that a restriction of GA biosynthesis or signalling is involved in the imposition of stereotypical floral development beyond the first flowers.

### GA-dependent effects on floral patterning were strongest in stamens

Defects in flower morphology are likely to have arisen early in floral development, pointing to previously unrecognised functions for GA during this stage. To specify these possible functions more closely, the observed floral abnormalities were subdivided into phenotypic classes and by floral organ type: sepals (whorl 1, outermost), petals (whorl 2), stamens (whorl 3) and the pistil (whorl 4, innermost). Given that lateral (or ‘short’) stamens within whorl three arise separately, after the emergence of medial (or ‘long’) stamens (Smyth et al. 1990), long and short stamens were treated as separate organ types. The phenotypic classes identified comprised deviations in the numbers of each floral organ type (gain or loss of organs relative to the expected number; 83.61% of abnormalities over all genotypes); organ fusion or splitting, here defined as the emergence of two partially or wholly fused organs from the same position within the floral plan (10.16%) and partial homeotic conversion of organ identity (6.23%).

To better understand the factors underlying these different types of abnormality, the relative contributions of genotype, flower position and GA treatment were re-assessed using GLM (see “Materials and methods”). When analysing the frequency of deviations from the expected number of floral organs across all floral organ types (whole flowers), a significant interaction was found only between GA treatment and flower position (*p* < 0.001; Table 3), whilst genotype was a significant independent factor (*p* < 0.001). Within individual organ types, only individual factors remained independently significant (Table 3). Similarly, no significant two- or three-way interactions were detected in relation to frequencies of floral organ fusion or homeosis for either whole flowers or individual organs (*p* > 0.05), but single factors remained significant (*p* < 0.05; Table 3). The interactions detected between experimental treatments when considering all floral abnormalities together and at the scale of the whole flower are thus likely to represent a combination of multiple independent phenotypic effects occurring at a smaller scale. GA treatment significantly affected the numbers of all floral organ types (*p* < 0.05) and the frequency of organ fusion events. In contrast, organ homeosis was not affected by GA treatment (*p* = 0.378). The effects of genotype were specific to short stamens, both in their number (*p* = 0.006) and the occurrence of homeotic events (*p* = 0.007). Flower position had a significant effect only on the numbers of both long and short stamens (*p* = 0.007 and *p* < 0.001, respectively). Thus, the effect of these factors on floral development can be interpreted more closely.

**Table 3:** Effect of genotype, exogenous GA treatment and flower position on the occurrence of floral abnormalities across the early *Arabidopsis* inflorescence

Phenotypic class	Level of analysis	Effects						
		Genotype	GA treatment	Flower position	Genotype: GA treatment	Genotype: flower position	GA treatment: flower position	Genotype: GA treatment: flower position
All floral abnormalities	Whole flower	0.004**	**<** 0.001***	**<** 0.001***	0.024*	0.033*	**<** 0.001***	0.807
Deviations in organ number	Whole flower	**<** 0.001***	**<** 0.001***	**<** 0.001***	0.096	0.136	**<** 0.001***	0.855
	Sepals	0.360	0.009**	0.533	0.882	1.000	0.996	1.000
	Petals	0.503	0.008**	0.822	0.836	1.000	0.995	1.000
	Long stamens	0.128	0.036*	0.007**	0.279	0.903	0.118	0.999
	Short stamens	0.006**	**<** 0.001***	**<** 0.001***	0.256	0.504	0.200	0.953
Organ fusion	Whole flower	0.573	0.004**	0.242	0.751	0.940	0.843	1.000
	Long stamens	0.377	0.002**	0.210	0.819	0.994	0.876	1.000
Organ homeosis	Short stamens	0.007**	0.378	0.608	0.817	1.000	0.963	1.000

Summary of *p* values for main effects (genotype, GA treatment and flower position) and interactions between them (denoted by colons) obtained via GLM (see “Materials and methods”). The floral abnormalities dataset was analysed at a series of hierarchical levels, parsing the data by phenotypic class of abnormality (deviations in organ number, organ fusion, organ homeosis) and the position (whorl) within the flower where the abnormality occurred. Where no abnormalities of a specific type occurred within a particular whorl, that analysis is not shown. Organ homeosis was only observed in short stamens. Statistically significant *p* values (*p* < 0.05) are highlighted in bold. The numbers of asterisks denotes increasing stringency of statistical significance

*p < 0.05, **p < 0.01, ***p < 0.001

**Fig. 6:**
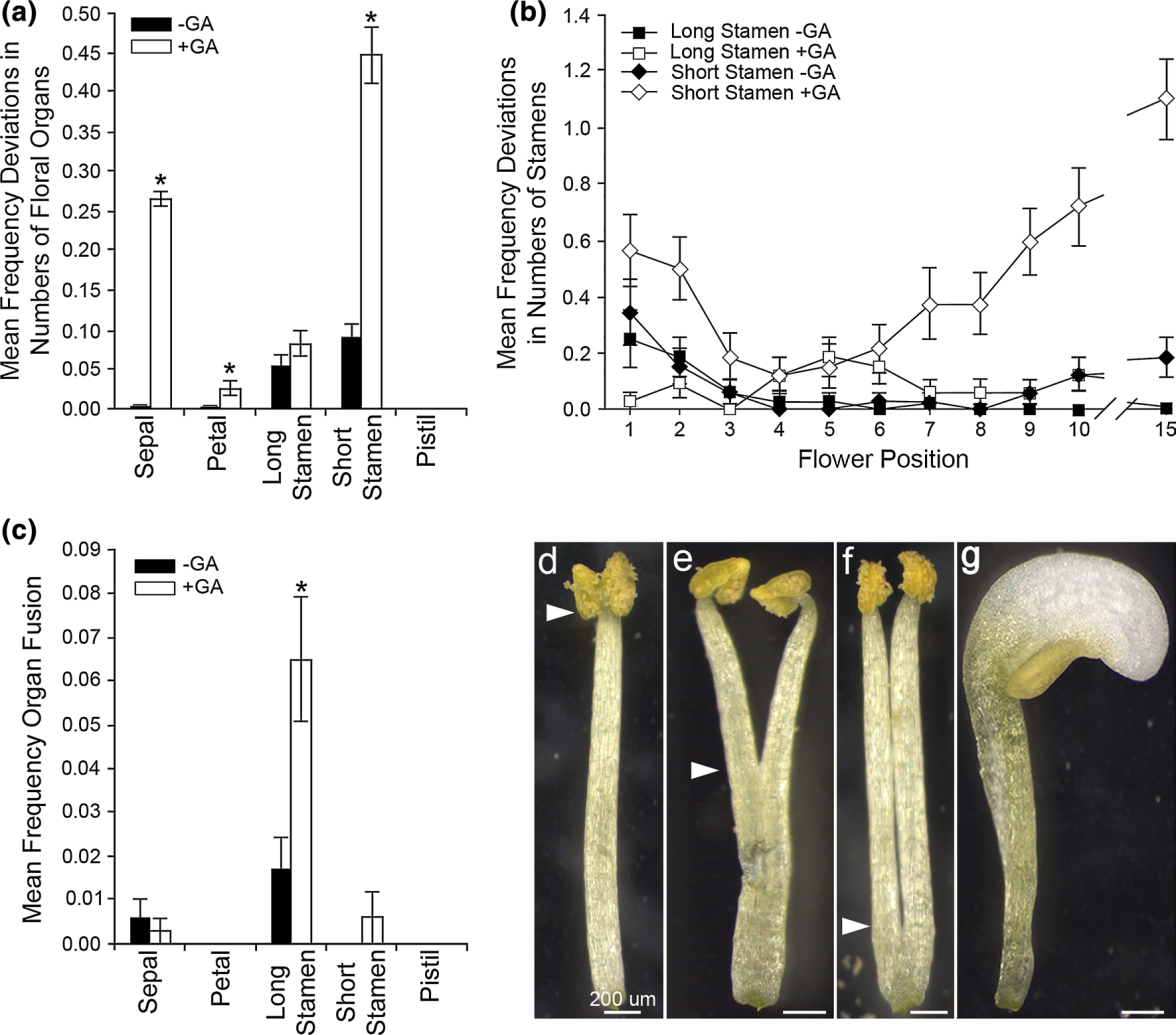
Exogenous GA treatment most strongly affects floral organ patterning in whorl three (stamens). **a** Mean frequencies of deviations in floral organ number for each floral organ type under control growth conditions (black) and exogenous GA treatment (white) averaged across all genotypes. Values shown are the mean of 352 independent flowers ± S.E. Asterisks indicate a significant difference between control and GA-treated conditions (*p* < 0.05). Comparisons were made using LSD values at a significance threshold of 5% (see Online Resource 5). **b** Plotted means of the frequencies of deviations in stamen numbers for long and short stamens under control growth conditions (black) and exogenous GA treatment (white) for flower positions 1–10 and 15, averaged across all genotypes. Values shown are the mean of 32 independent flowers ± S.E. At the level of long and short stamens, both GA treatment and flower position have significant (*p* < 0.05) effects on the frequency of deviations, but there was no significant interaction between the two (long: *p* = 0.118, short: *p* = 0.200; Table 3). **c** Mean frequencies of organ fusion events for each floral organ type under control growth conditions (black) and exogenous GA treatment (white) averaged across all genotypes. Values shown are the mean of 352 independent flowers ± S.E. Asterisks indicate a significant difference between control and GA-treated conditions (*p* < 0.05). Comparisons were made using LSD values at a significance threshold of 5% (see Online Resource 5). **d–f** Examples of organ fusion in stamens; white arrows mark the point of fusion/splitting between two organs. **g** Example of stamen homeosis, with conversion of half the organ to petal identity. This phenotype was observed only in short stamens

Changes observed in the number of floral organs were not uniform: while only additional organs were detected for sepals, both organ loss and gain were observed for petals and both stamen types (see Online Resource 5). Under control growth conditions, deviations in the number of floral organ occurred most frequently in long and short stamens (Fig. 6a). GA treatment significantly increased the frequency of deviations in the numbers of sepals, petals and short stamens (*p* < 0.05; Fig. 6a). Only increases in net sepal number were recorded under GA treatment, whereas short stamens were almost always lost. Losses and gains of long stamens were detected under both growth conditions (see Online Resource 5). Deviations in the numbers of both long and short stamens occurred most frequently in the first flowers to open (Fig. 6b). Under GA treatment, more deviations in the numbers of short stamens occurred across the early inflorescence (Fig. 6b), although these appeared increasingly frequent in later flowers. The effect on the numbers of long stamens was much less pronounced. These changing distributions of floral organ numbers amongst the different floral organ types in response to GA treatment could reflect changes in whorl boundaries early in FM development, or potentially changes in FM starting size.

Under control growth conditions, organ fusion events were detected in sepals and long stamens only and were most common in long stamens (Fig. 6c). GA treatment significantly increased the occurrence of organ fusion in long stamens only (*p* < 0.05; Fig. 6c). Varying degrees of fusion between long stamens were observed under both control growth conditions and under GA treatment (Fig. 6d–f). In contrast, homeosis was found only in short stamens, which always exhibited partial conversion to petal fate (Fig. 6g). These results indicate that stamens are the floral organ most susceptible to perturbation during early flowering, with all three types of developmental abnormality presenting within whorl 3. The frequency of two of these abnormalities is increased within the early inflorescence under exogenous GA treatment. Whilst there are known regulatory roles for GA during later stamen development (reviewed in Plackett et al. 2011), this is the first phenotypic evidence of a role during earlier floral development when organ number and identity are set. Flower position was found to represent a significant factor in determining the numbers of long and short stamens, which likely account for the position-specific effects identified when analysing all organ types together (cf. Fig. 5c). The differential phenotypic responses between long and short stamens could indicate that long and short stamens might differ in some of the signalling pathways underlying their development.

**Fig. 7:**
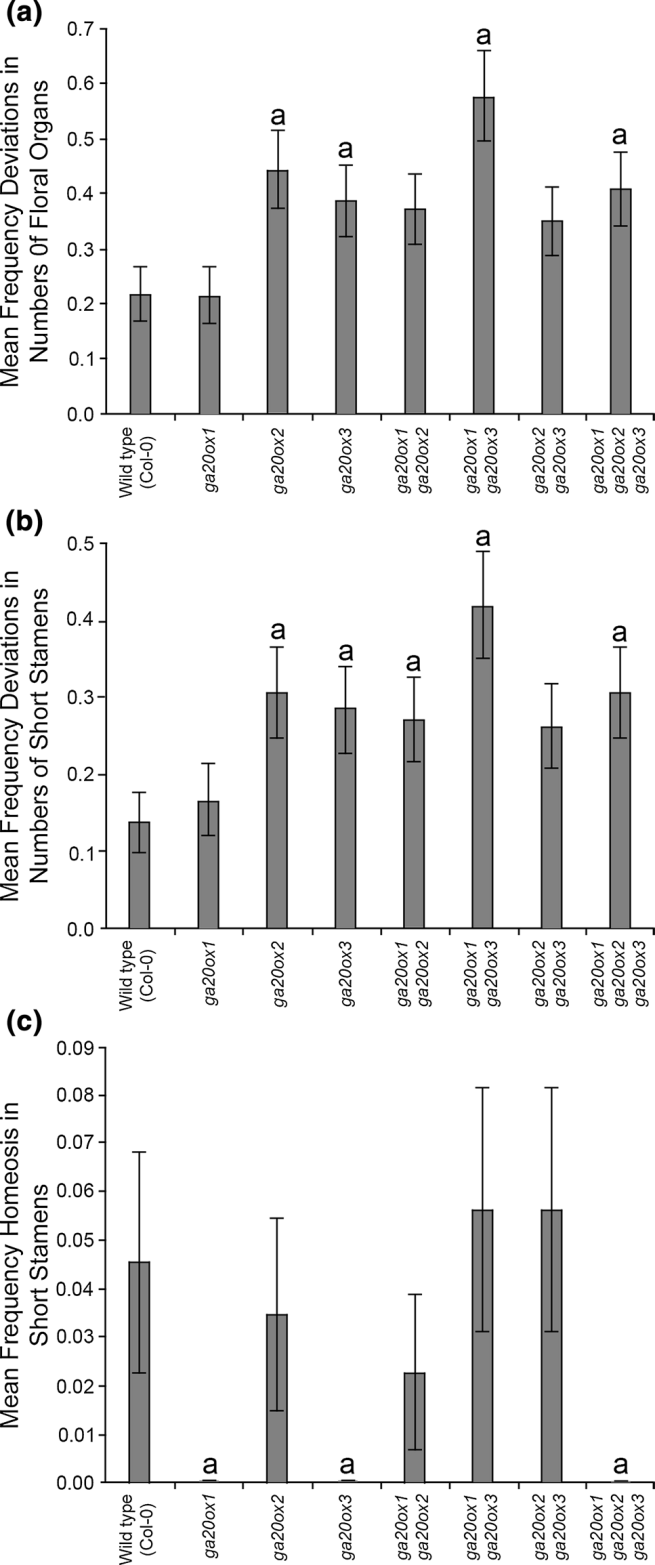
*ga20ox* mutants demonstrate differing short stamen phenotypes. Mean frequencies of deviations in floral organ number across the whole flower (**a**) and within short stamens alone (**b**), and the mean frequencies of short stamen homeosis (**c**), by genotype, averaged across growth conditions and all flower positions. Values shown are the mean of 352 independent flowers ± S.E. Letters denote a significant difference (*p* < 0.05) from wild type. Genotypes marked by different letters are significantly different from one another (*p* < 0.05). Pairwise comparisons were made using LSDs with a significance threshold of 5% (see Online Resource 5)

As described above, when the frequencies of all floral abnormalities were considered collectively, *ga20ox1* and *ga20ox1 ga20ox3* showed significant differences from wild type (Fig. 5a), but demonstrated contrary differences (Fig. 5a, b). However, genotype is only a significant factor within short stamens (Table 3), affecting both numbers of short stamens and short stamen homeosis. This argues that the differences between *ga20ox1* and *ga20ox1 ga20ox3* centre on short stamen development. In support of this, the effects of genotype on the deviations in numbers of all floral organs (Fig. 7a) can mostly be accounted for through genotype effects on short stamens (Fig. 7b). Within short stamens, a significant difference remained between *ga20ox1 ga20ox3* and wild type (*p* < 0.05; Fig. 7b). Other genotypes also demonstrated significant differences in short stamen number compared to wild type, including *ga20ox1 ga20ox2 ga20ox3* (*p* < 0.05; Fig. 7b), but interestingly *ga20ox1* did not (*p* > 0.05). In contrast, the frequency of short stamen homeosis in *ga20ox1* was significantly reduced compared to the wild type (*p* < 0.05; Fig. 7c), whereas homeosis in *ga20ox1 ga20ox3* short stamens was not significantly different from wild type (*p* > 0.05). These results reconcile the apparently contradictory phenotypes of *ga20ox1* and *ga20ox1 ga20ox3* found at the level of all floral abnormalities, with the two genotypes affecting separate processes in stamen development.

## Discussion

### Floral organ growth is linked within and between flowers of the Arabidopsis inflorescence

In this work, we demonstrate the presence of significant gradients of floral organ growth in the wild-type *Arabidopsis* inflorescence: stamen length at flower opening increases with advancing flower position, whilst pistil length remains static. These results indicate that *Arabidopsis* floral development is not independent of flower position on the early inflorescence and that floral development changes as flowering progresses. This differential growth between stamen and pistil is consistent with an identified trend of increasing probability of silique-set across the early inflorescence. Stamen growth continues after flower opening (Smyth et al. 1990), so changing stamen growth could reflect either increased final stamen length in later flowers or accelerated growth in later flowers to achieve the same length earlier in development. Within the same flowers, sepals demonstrated an opposing growth gradient, indicating that these changes are not simply a factor of increasing flower size overall. Floral organs are initiated in concentric whorls, beginning with sepals as the outermost organs and then progressing inwards (Smyth et al. 1990). Thus, changes in size between different organ types could reflect changes during early floral development, i.e. the timing of organ specification or the size of founder cell populations. The adaptive advantage to the existence of such gradients is as yet unclear, but could represent a mechanism through which plants can improve the chance of successful self-pollination under unfavourable environmental conditions (see below). Alternatively, outcrossing in plant species is usually achieved through differential growth of male and female reproductive organs—for example delaying stamen maturation until the pistil has been pollinated (Holtsford and Ellstrand 1992; Sherry and Lord 2000). The gradients found here, during flowering of this typically self-pollinating *Arabidopsis* laboratory strain, may be related to this mechanism, or act to increase the likelihood of outcrossing in early flowers as part of a bet-hedging strategy to improve reproductive success.

Modelling of floral organ growth across *ga20ox* mutant inflorescences found that significant gradients in floral organ growth persisted, even in the severely GA-deficient *ga20ox1 ga20ox2 ga20ox3*. The differences in stamen and pistil growth parameters between *ga20ox* mutants are sufficient to explain the silique-set phenotypes observed, and we identify potentially non-redundant roles for *GA20ox3*. The finding that reduced silique-set in *ga20ox1* is caused by increased pistil growth is surprising, as previous studies have supported expression of *GA20ox1* within the stamen and not the pistil (Plackett et al. 2012). However, the expression of *GA20ox1*, *GA20ox2* and *GA20ox3* has been shown to be interrelated through complex feedback mechanisms (Rieu et al. 2008). *GA20ox2* is expressed in pistil tissues during flower development (Plackett et al. 2012) and is significantly up-regulated in *ga20ox1* plant tissues (Rieu et al. 2008). Thus, feedback up-regulation of *GA20ox2* could feasibly enhance GA biosynthesis in the *ga20ox1* pistil and promote increased growth. Predictions of *GA20ox* gene expression changes in mutant backgrounds are complicated further by feedback links to *GA3ox* (GA biosynthesis) and *GA2ox* (GA catabolism) gene expression (Yamaguchi 2008). Reduced pistil growth in the *ga20ox2* mutant, as identified by linear modelling, is consistent with reported *GA20ox2* pistil expression patterns (Plackett et al. 2012), and neither *GA20ox1* nor *GA20ox3* are up-regulated in *ga20ox2* tissues (Rieu et al. 2008). Recent quantification of GA levels from the organs of single wild-type flowers has confirmed the presence of bioactive GA specifically in the pistil, stamen and the receptacle of newly opened flowers, although the flower position sampled on the inflorescence is not indicated (Li et al. 2017).

Both the success of silique-set and floral organ lengths were found to contain inherent variability when comparing between adjacent flower positions along the early inflorescence. In addition, whilst we observed floral phenotypes for early *ga20ox1 ga20ox2* flowers similar to those previously reported (Rieu et al. 2008), the observed rates at which spontaneous silique-set recovered appeared to differ between flower position 10 (this study) and approximately flower position 15 (Rieu et al. 2008). Thus, whilst we necessarily refer to differences between specific floral positions based on the results obtained in this study, the positions in themselves are unlikely to hold large biological relevance to the underlying mechanism and instead reflect positions on a gradient that might itself be subject to variability. The most likely cause of the difference between these two studies is uncontrolled small variations in experimental growth conditions. Similar phenotypic discrepancies in response to growth conditions have been noted previously in relation to floral development in the *GIBBERELLIN INSENSTIVE DWARF 1* (*GID1*) GA receptor triple mutant (Iuchi et al. 2007; Griffiths et al. 2007; Willige et al. 2007; Plackett et al. 2014). Environmental factors such as temperature are known to have a significant impact on plant fertility, for example through pollen development and fertilisation (Zinn et al. 2010; De Storme and Geelan 2014). In some plant species, environmental stress results in differential rates of self-pollination and outcrossing through impaired male fertility (Bishop et al. 2017). An interaction between *Arabidopsis* pollen development, GA and temperature sensitivity has previously been uncovered through the GA signalling negative regulator *SPINDLY1* (*SPY1*) (Jacobsen and Olszewski 1993), which is also involved in regulating responses to other abiotic stresses (Qin et al. 2011). Pollen development in the *Arabidopsis GAMYB* mutant *myb33 myb65* also demonstrates increased temperature sensitivity (Millar and Gublar 2005). It would be interesting to determine the extent to which floral organ growth patterns across the inflorescence are influenced by environmental factors.

An important question that remains is what are the pathways through which these gradients are achieved and to what extent they are GA dependent. Significant gradients were found to persist in GA-deficient backgrounds, and gradual recovery of stamen growth in the severely GA-deficient *ga1*-*3* mutant has also previously been reported during late flowering (Plackett et al. 2012). As such, these gradients are unlikely to be generated solely through changes in endogenous GA biosynthesis. The presence of bioactive GA is transduced into plant developmental responses through GA-dependent binding of the GID1 receptor protein to growth-repressing DELLA proteins, triggering their rapid degradation (Harberd et al. 2009). There are five DELLA paralogues in *Arabidopsis*, *GA INSENSTIVE* (*GAI*), *REPRESSOR OF GAI* (*RGA*) and *RGA*-*LIKE 1* (*RGL1*), *RGL2* and *RGL3*. These demonstrate differential expression levels in flowers (Tyler et al. 2004), but their precise tissue expression patterns remain unresolved. *Arabidopsis* mutants lacking or with impaired DELLA function show increased elongation of pistils, stamens and petals compared to wild type (Cheng et al. 2004; Fuentes et al. 2012), demonstrating that under normal development their growth remains under some level of repression. Flowers of the DELLA *global* mutant (lacking all five DELLA paralogues) in the *Arabidopsis* L*er* ecotype self-pollinate successfully, but have reduced post-pollination fertility (Fuentes et al. 2012). This mutant does demonstrate some reduction in silique-set compared to wild type L*er*, but this is not specific to early flowering (Plackett et al. 2014). *RGA*, *RGL1* and *RGL2* are the dominant DELLA paralogues regulating floral organ growth in L*er* (Cheng et al. 2004). A spontaneous late-flowering recovery of silique-set has been reported in L*er ga1*-*3* mutants lacking two specific combinations of DELLA paralogue, *GAI* and *RGA* or *RGA* and *RGL2* (Cheng et al. 2004). The early flowers of *rga*-*24 gai*-*t6* show impaired floral organ growth similar to *ga1*-*3* (Dill and Sun 2001; King et al. 2001), implying that in at least this line the late-stage rescue in silique-set occurs through recovered floral organ growth. Cheng et al. (2004) also found that *ga1*-*3* plants lacking *GAI, RGA*, *RGL1* and *RGL2* produce fertile flowers.

These data, the *global* mutant phenotype and the reported lack of phenotypic recovery in other pairwise combinations of DELLA paralogue in the *ga1*-*3* background (Cheng et al. 2004) suggest that the observed gradients in floral organ growth along the inflorescence do relate directly to the GA signalling pathway, although it would be interesting to test directly for changes in floral organ growth across the inflorescence in non-silique-setting mutants, as silique-set can also be inhibited by reductions in male or female fertility under chemically or genetically GA-overdosed conditions (Jacobsen and Olszewski 1993; Cheng et al. 2004; Fuentes et al. 2012; Plackett et al. 2014). It can also be hypothesised that the mechanisms underlying changing floral organ growth across the inflorescence acts either through modulating GA signal transduction itself or downstream of GA signalling. It would be interesting to determine whether changes in floral organ growth across the inflorescence also occur independently of DELLA-dependent signalling, for example in the *gid1* triple receptor mutant or the degradation-resistant DELLA mutant *gai*-*1* (Dill et al. 2001).

### The recovery of *ga20ox1 ga20ox2* floral organ growth is associated with an implied late-stage developmental checkpoint

Evidence presented in this study suggests that anther indehiscence in the earliest *ga20ox1 ga20ox2* flowers is correlated with a severe reduction of stamen (and petal) growth not seen in flowers with dehiscent anthers at the same flower position. This effect is overlaid onto a gradient of stamen growth similar to that seen in wild type, albeit with a reduced initial stamen length. Anther indehiscence was previously described in early flowers of *ga3ox* GA biosynthesis mutants (Hu et al. 2008), where stamen development apparently arrested late in flower development (stages 11 or 12). Stamen filament elongation primarily occurs in synchrony with anther maturation during late floral development, and stamen elongation in later *ga20ox1 ga20ox2* flowers is reduced at this stage (Plackett et al. 2012). The results of this study therefore support the existence of a GA-dependent, late-stage anther developmental block and further suggest a link, directly or indirectly, with late-stage stamen filament growth.

Mechanisms linking anther development with filament elongation are currently unknown. The sharp difference in filament lengths between stamens with dehiscent and indehiscent anthers argues against GA itself acting to coordinate the two tissues: in this scenario the relationship between stamen length and GA concentration would be expected to be continuous (analogue). Supporting this is the observation that recent sensitive assays were unable to detect bioactive GA in wild-type petals at flower opening (Li et al. 2017). Furthermore, although their average growth was reduced across the early *ga20ox1 ga20ox2* inflorescence, growth of pistils and sepals was not affected in flowers with indehiscent anthers. An alternative candidate is the phytohormone jasmonate (JA): the JA biosynthetic mutant *opr3* displays arrested anther development and inhibited stamen filament elongation (Stintzi and Browse 2000). However, petal development is apparently unaffected in this mutant. In contrast, the JA biosynthesis mutant *defective in anther dehiscence 1* (*dad1*) demonstrates reduced growth of both stamens and petals during late floral development (Ishiguro et al. 2001). *DAD1* expression is restricted to the stamen filament during floral development (Ishiguro et al. 2001), where it is up-regulated by GA signalling (Cheng et al. 2009), although treatment with either of these hormones cannot entirely rescue floral phenotypes caused by deficiency of the other (Cheng et al. 2009), suggesting the requirement for other downstream signalling cascades.

How *ga20ox1 ga20ox2* stamen development spontaneously overcomes this developmental block remains unclear, but presumably relates to changes in signalling along the early inflorescence. Although *GA20ox3* has been shown to be up-regulated in the *ga20ox1 ga20ox2* pistil and stamen filament (Plackett et al. 2012), Hu et al. (2008) detected no difference in GA levels between early and late *ga3ox* mutant flowers displaying a similar phenotypic recovery, making it unlikely that *ga20ox1 ga20ox2* stamen development recovers through direct changes in GA biosynthesis or signalling. Alternatively, pathways downstream of GA signalling might be modulated to promote continued development despite the absence of GA. Our results suggest that any such changes are likely to be located within the developing anther.

### Novel roles for GA in floral patterning and stamen development

*Arabidopsis* floral development is considered to be a highly determinant, invariant programme of events generating a fixed number of floral organs in sequential, concentric whorls from a floral meristem (FM). Some of the regulatory mechanisms governing these events are already known (reviewed in Airoldi 2010; Irish 2010). However, we detected a significantly higher incidence of abnormal floral development in the first flowers to emerge compared to later flowers across all genotypes. *Arabidopsis* FMs are produced sequentially from the flanks of the inflorescence meristem (IM), in place of leaf primordia generated during the vegetative phase of development. The positions of lateral organ primordia are determined through auxin maxima in both the vegetative and reproductive phases, the positions of which are influenced by the positions of preceding primordia (Reinhardt et al. 2003; Heisler et al. 2005). Both leaf primordia and FMs are generated in a spiral phyllotaxy, the direction of which is not predetermined and which can change between developing inflorescences on the same plant (Smyth et al. 1990). During the transition of the shoot apical meristem (SAM) to IM identity, the apex increases in size and changes shape (Mishke and Brown 1965). Development in the first FMs may thus be more plastic because they arise without pre-existing primordia of the same type to constrain developmental patterning.

Exogenous GA treatment did not increase the frequency of abnormalities in the first flowers to develop but prolonged their occurrence into later flowering, suggesting that it is necessary to restrict GA signalling to stabilise early floral development. In support of this, we noticed that inflorescence phyllotaxy appeared disturbed in plants grown under GA treatment (Online Resource 4). The GA biosynthetic genes *CPS* and *GA3ox1* are both expressed in early FMs prior to the outgrowth of the floral organs (Hu et al. 2008). Until now no developmental functions have been ascribed to GA at this stage of floral development. The FM identity gene *LEAFY* (*LFY*) has been shown to restrict GA signalling through enhanced GA catabolism to trigger up-regulation of its downstream target *APETALA1* (*AP1*) via DELLA accumulation (Yamaguchi et al. 2014). Our analysis of floral abnormalities at the whole flower level suggested that imbalanced GA signalling across the FM, rather than GA signalling per se, triggers abnormal development. Two genotypes, *ga20ox1* and *ga20ox1 ga20ox3*, had apparently contradictory effects on the frequency of abnormalities as a result of influencing different developmental processes (short stamen homeosis and short stamen numbers, respectively). The expression patterns of the *GA20ox* paralogues have not been mapped within the early FM, but could cause localised differences in bioactive GA levels through regulating local availability of the GA3ox substrate GA_9_.

We identified significant deviations in the numbers of floral organs produced under GA treatment. Changes to the number of organs that develop might reflect alterations to the relative sizes of the concentric whorls specified within the FM, either directly regulated by GA signalling or indirectly through a change in initial FM size: a larger FM could allow more sepals to arise at the periphery (as was found), the altered geometry subsequently affecting patterning in the later-developing inner whorls. The tendency towards loss of short stamens can also be explained by such a mechanism, as an outcome of their late emergence after the establishment of long stamens (Smyth et al. 1990). However, we did not find any clear correlation between changes in the number of organs across different whorls. Organ fusion events indicate defects in organogenesis beyond organ specification. Separate floral organ primordia are established within each whorl by the creation of boundaries between adjacent organs, comprised of specialised cell types (reviewed in Aida and Tasaka 2006; Wang et al. 2016). A number of transcription factors regulate stamen boundary formation, including *CUP*-*SHAPED COTYLEDON1* (*CUC1*) and *CUC2* (Aida et al. 1997), *FUSED FLORAL ORGANS1* (*FFO1*), *FFO2* and *FFO3* (Levin et al. 1998), *UNUSUAL FLORAL ORGANS (UFO*; Laufs et al. 2003) and *HANABA TARANU* (*HAN*; Zhao et al. 2004). Organ loss and/or stamen fusion has been observed in a number of mutants for these genes, and auxin signalling has been implicated as an organising factor (Furutani et al. 2004). The changes in organ number and organ fusions observed in this study could arise through mis-regulation of these or other boundary genes in very early flowers and under exogenous GA treatment. Regulation by GA might be direct, or changes in size of the initial FM or whorl domain might result in indirect perturbations downstream.

Abnormalities in floral organ development occurred most frequently in stamens. Similar stamen defects arise when auxin biosynthesis or polar auxin transport are disrupted (Cardarelli and Cecchetti 2014). Later stages of stamen development are heavily regulated by GA signalling (reviewed in Plackett et al. 2011). Floral organ identity is specified through interactions of MADS-box genes following the ABCE model (reviewed in Irish 2010): stamens are determined by interaction between the B class MADS-box genes *PISTILLATA* (*PI*) and *APETALA3* (*AP3*) and the C class gene *AGAMOUS* (*AG*). *AP3*, *PI* and *AG* are all known targets of GA signalling, either directly or via the GA downstream target *LFY* (Blázquez et al. 1998; Wagner et al. 1999; Yu et al. 2004). Prolonged *AG* expression during floral development is required to maintain stamen identity (Bowman et al. 1991; Ito et al. 2007), and as well as being a downstream target of GA signalling, AG up-regulates *GA3ox1* (Gómez-Mena et al. 2005). Stamens are converted to petals in *ag* loss-of-function mutants (Coen and Meyerowitz 1991) and GA treatment can also promote petal development in *ap1* and *ap2* homeotic mutant backgrounds (Okamuro et al. 1997), where petals are converted to stamen identity. The homeotic short stamen phenotype observed here is presumably linked to GA-dependent changes in A, B or C gene expression, although mis-regulation of some boundary genes such as *UFO* can also lead to the formation of mosaic floral organ identities (Levin and Meyerowitz 1995).

The analysis of *Arabidopsis* early flowering presented here has thus identified new potential roles for GA signalling during the very early stages of floral development. The developmental plasticity demonstrated by the first flowers represents an excellent vehicle to better understand the regulation of these early development processes.

#### Author contribution statement

ALP, PH, ST and ZAW designed research. ARGP conducted experimental work. SJP conducted modelling and statistical analysis. All authors read and approved the manuscript.

## Electronic supplementary material

Below is the link to the electronic supplementary material.

Supplementary material 1 (PDF 77 kb)

Supplementary material 2 (PDF 88 kb)

Supplementary material 3 (PDF 314 kb)

Supplementary material 4 (PDF 968 kb)

Supplementary material 5 (PDF 400 kb)
