# Supplementary material for: The early inflorescence of Arabidopsis thaliana demonstrates positional effects in floral organ growth and meristem patterning
Source: Plant Reprod. 2017 Dec 20;31(2):171–91. doi: 10.1007/s00497-017-0320-3 (PMC5940708; doi:10.1007/s00497-017-0320-3)
Supplement: Supplementary file 1 — Supplementary material 1 (PDF 77 kb) [file 497_2017_320_MOESM1_ESM.pdf]

ONLINE RESOURCE 1: Predicted means and LSD values (1%) of silique-set during early flowering.

Article Title: The early inflorescence of *Arabidopsis thaliana* demonstrates positional effects in floral organ growth and meristem patterning

Journal: Plant Reproduction

Authors: ARG Plackett, SJ Powers, AL Phillips, ZA Wilson, P Hedden, SG Thomas

Corresponding author: ARG Plackett
Address: University of Cambridge, Department of Plant Sciences, Downing Street, Cambridge, CB2 3EA, UK
E-mail: arp74@cam.ac.uk

1a. Means for the probability of silique-set (averaged across all flower positions) and LSD (1%) values for comparison, arising from the significant interaction between genotype and GA treatment ($p < 0.001$. Fig. 1a).

GA	GA-		GA+	
	Prediction	s.e.	Prediction	s.e.
Geno				
A	0.9249	0.01812	0.4250	0.04350
B	0.5217	0.03675	0.4338	0.04326
C	0.9600	0.01894	0.3917	0.04243
D	0.9099	0.02179	0.4061	0.04269
E	0.2075	0.03403	0.3884	0.04338
F	0.4710	0.04224	0.3083	0.03978
G	0.9131	0.02721	0.3833	0.04028
H	0.0000	0.00028	0.3431	0.04307
I	0.0000	0.00029	0.1670	0.03673

Least significant differences of predictions (1% level)

Geno A GA GA-	1	*				
Geno A GA GA+	2	0.1215	*			
Geno B GA GA-	3	0.1057	0.1469	*		
Geno B GA GA+	4	0.1209	0.1582	0.1462	*	
Geno C GA GA-	5	0.0676	0.1224	0.1067	0.1218	*
Geno C GA GA+	6	0.1190	0.1567	0.1448	0.1563	0.1198
Geno D GA GA-	7	0.0731	0.1255	0.1102	0.1249	0.0744
Geno D GA GA+	8	0.1196	0.1572	0.1454	0.1568	0.1204
Geno E GA GA-	9	0.0994	0.1424	0.1290	0.1417	0.1005
Geno E GA GA+	10	0.1212	0.1584	0.1464	0.1577	0.1221
Geno F GA GA-	11	0.1185	0.1564	0.1445	0.1560	0.1194
Geno F GA GA+	12	0.1127	0.1520	0.1397	0.1515	0.1136
Geno G GA GA-	13	0.0843	0.1323	0.1179	0.1319	0.0854
Geno G GA GA+	14	0.1139	0.1529	0.1406	0.1524	0.1148
Geno H GA GA-	15	0.0467	0.1122	0.0948	0.1115	0.0488
Geno H GA GA+	16	0.1205	0.1578	0.1459	0.1575	0.1214
Geno I GA GA-	17	0.0467	0.1122	0.0948	0.1115	0.0488
Geno I GA GA+	18	0.1056	0.1468	0.1342	0.1464	0.1066
		1	2	3	4	5

Geno C GA GA+	6	*				
Geno D GA GA-	7	0.1230	*			
Geno D GA GA+	8	0.1552	0.1236	*		
Geno E GA GA-	9	0.1403	0.1042	0.1408	*	
Geno E GA GA+	10	0.1565	0.1252	0.1570	0.1420	*
Geno F GA GA-	11	0.1544	0.1226	0.1549	0.1399	0.1562
Geno F GA GA+	12	0.1500	0.1169	0.1505	0.1350	0.1518
Geno G GA GA-	13	0.1300	0.0899	0.1305	0.1124	0.1322
Geno G GA GA+	14	0.1509	0.1181	0.1514	0.1360	0.1527
Geno H GA GA-	15	0.1094	0.0562	0.1101	0.0878	0.1119
Geno H GA GA+	16	0.1559	0.1245	0.1565	0.1416	0.1578
Geno I GA GA-	17	0.1094	0.0562	0.1101	0.0878	0.1119
Geno I GA GA+	18	0.1447	0.1101	0.1453	0.1292	0.1467
		6	7	8	9	10

Geno F GA GA-	11	*			
Geno F GA GA+	12	0.1496	*		
Geno G GA GA-	13	0.1297	0.1243	*	
Geno G GA GA+	14	0.1505	0.1460	0.1254	*

Geno H GA GA-	15	0.1089	0.1026	0.0702	0.1039	*
Geno H GA GA+	16	0.1553	0.1512	0.1314	0.1520	0.1111
Geno I GA GA-	17	0.1089	0.1026	0.0702	0.1039	0.0010
Geno I GA GA+	18	0.1442	0.1396	0.1179	0.1405	0.0947
		11	12	13	14	15

Geno H GA GA+	16	*		
Geno I GA GA-	17	0.1111	*	
Geno I GA GA+	18	0.1460	0.0947	*
		16	17	18

Genotypes are A (Wild Type Col-0), B (*ga20ox1*), C (*ga20ox2*), D (*ga20ox3*), E (*ga20ox1 ga20ox2*), F (*ga20ox1 ga20ox3*), G (*ga20ox2 ga20ox3*), H (*ga20ox1 ga20ox2 ga20ox3*), I (*ga1-3*(Col-0))

1b. Means for the probability of silique-set (averaged across all genotypes) and LSD (1%) values for comparison, arising from the significant interaction between GA treatment and flower position ($p < 0.001$, Fig. 1b).

Bud	1		2	
	Prediction	s.e.	Prediction	s.e.
GA				
GA-	0.2844	0.03249	0.4296	0.02874
GA+	0.1401	0.03301	0.2636	0.04061

Bud	3		4	
	Prediction	s.e.	Prediction	s.e.
GA				
GA-	0.4948	0.02652	0.5343	0.03067
GA+	0.2081	0.03880	0.3542	0.04479

Bud	5		6	
	Prediction	s.e.	Prediction	s.e.
GA				
GA-	0.5683	0.02553	0.5779	0.03031
GA+	0.3563	0.04499	0.3875	0.04662

Bud	7		8	
	Prediction	s.e.	Prediction	s.e.
GA				
GA-	0.6193	0.02872	0.6313	0.02391
GA+	0.4265	0.04679	0.5322	0.04630

Bud	9		10	
	Prediction	s.e.	Prediction	s.e.
GA				
GA-	0.6830	0.02536	0.7033	0.02504
GA+	0.4456	0.04868	0.5121	0.04810

Least significant differences of predictions (1% level)

GA GA- Bud 1	1	*				
GA GA- Bud 2	2	0.1118	*			
GA GA- Bud 3	3	0.1082	0.1008	*		
GA GA- Bud 4	4	0.1152	0.1084	0.1045	*	
GA GA- Bud 5	5	0.1065	0.0991	0.0949	0.1029	*
GA GA- Bud 6	6	0.1146	0.1077	0.1038	0.1112	0.1022
GA GA- Bud 7	7	0.1118	0.1048	0.1008	0.1083	0.0991
GA GA- Bud 8	8	0.1040	0.0964	0.0921	0.1003	0.0902
GA GA- Bud 9	9	0.1063	0.0989	0.0946	0.1026	0.0928
GA GA- Bud 10	10	0.1058	0.0983	0.0941	0.1021	0.0922
GA GA+ Bud 1	11	0.1194	0.1129	0.1092	0.1162	0.1076
GA GA+ Bud 2	12	0.1341	0.1283	0.1251	0.1312	0.1237
GA GA+ Bud 3	13	0.1305	0.1245	0.1212	0.1275	0.1198
GA GA+ Bud 4	14	0.1427	0.1372	0.1342	0.1400	0.1329
GA GA+ Bud 5	15	0.1431	0.1377	0.1347	0.1404	0.1334
GA GA+ Bud 6	16	0.1465	0.1412	0.1383	0.1439	0.1371
GA GA+ Bud 7	17	0.1469	0.1416	0.1387	0.1443	0.1374
GA GA+ Bud 8	18	0.1459	0.1405	0.1376	0.1432	0.1363
GA GA+ Bud 9	19	0.1509	0.1458	0.1429	0.1484	0.1417
GA GA+ Bud 10	20	0.1497	0.1445	0.1416	0.1471	0.1404
		1	2	3	4	5

GA GA- Bud 6	6	*				
GA GA- Bud 7	7	0.1076	*			
GA GA- Bud 8	8	0.0995	0.0963	*		
GA GA- Bud 9	9	0.1019	0.0988	0.0899	*	
GA GA- Bud 10	10	0.1014	0.0982	0.0893	0.0919	*
GA GA+ Bud 1	11	0.1156	0.1128	0.1051	0.1073	0.1068
GA GA+ Bud 2	12	0.1307	0.1282	0.1215	0.1235	0.1230
GA GA+ Bud 3	13	0.1269	0.1245	0.1175	0.1195	0.1191
GA GA+ Bud 4	14	0.1394	0.1372	0.1309	0.1327	0.1323
GA GA+ Bud 5	15	0.1399	0.1376	0.1314	0.1332	0.1328
GA GA+ Bud 6	16	0.1434	0.1412	0.1351	0.1368	0.1365
GA GA+ Bud 7	17	0.1437	0.1416	0.1355	0.1372	0.1368
GA GA+ Bud 8	18	0.1427	0.1405	0.1344	0.1361	0.1357
GA GA+ Bud 9	19	0.1479	0.1457	0.1398	0.1415	0.1412
GA GA+ Bud 10	20	0.1466	0.1445	0.1385	0.1402	0.1398
		6	7	8	9	10

GA GA+ Bud 1	11	*				
GA GA+ Bud 2	12	0.1349	*			
GA GA+ Bud 3	13	0.1314	0.1448	*		
GA GA+ Bud 4	14	0.1435	0.1559	0.1528	*	
GA GA+ Bud 5	15	0.1439	0.1563	0.1532	0.1637	*
GA GA+ Bud 6	16	0.1473	0.1594	0.1564	0.1667	0.1671
GA GA+ Bud 7	17	0.1477	0.1597	0.1567	0.1670	0.1674
GA GA+ Bud 8	18	0.1466	0.1588	0.1558	0.1661	0.1665
GA GA+ Bud 9	19	0.1517	0.1635	0.1605	0.1706	0.1709
GA GA+ Bud 10	20	0.1504	0.1623	0.1593	0.1695	0.1698
		11	12	13	14	15

GA GA+ Bud 6	16	*				
GA GA+ Bud 7	17	0.1703	*			
GA GA+ Bud 8	18	0.1694	0.1697	*		
GA GA+ Bud 9	19	0.1738	0.1741	0.1732	*	
GA GA+ Bud 10	20	0.1727	0.1730	0.1721	0.1765	*
		16	17	18	19	20
