# Supplementary material for: The early inflorescence of Arabidopsis thaliana demonstrates positional effects in floral organ growth and meristem patterning
Source: Plant Reprod. 2017 Dec 20;31(2):171–91. doi: 10.1007/s00497-017-0320-3 (PMC5940708; doi:10.1007/s00497-017-0320-3)
Supplement: Supplementary file 2 — Supplementary material 2 (PDF 88 kb) [file 497_2017_320_MOESM2_ESM.pdf]

ONLINE RESOURCE 2: Predicted means and LSD values (5%) of floral organ lengths during early flowering.

Article Title: The early inflorescence of *Arabidopsis thaliana* demonstrates positional effects in floral organ growth and meristem patterning

Journal: Plant Reproduction

Authors: ARG Plackett, SJ Powers, AL Phillips, ZA Wilson, P Hedden, SG Thomas

Corresponding author: ARG Plackett
Address: University of Cambridge, Department of Plant Sciences, Downing Street, Cambridge, CB2 3EA, UK
E-mail: arp74@cam.ac.uk

2a. Means for stamen length as a percentage of the pistil (Fig. 2q) used in ANOVA, with associated SED, df and LSD (5%) values.

Geno	GA	Flower	1	2	3	4	5
A	GA-		100.96	106.36	107.20	109.48	108.02
	GA+		100.43	104.58	106.07	110.05	110.50
B	GA-		93.36	93.85	98.96	98.13	109.21
	GA+		100.33	100.63	103.40	108.97	100.58
C	GA-		103.59	118.76	121.80	119.97	123.02
	GA+		108.16	108.93	107.41	115.39	109.34
D	GA-		102.85	106.47	113.24	113.31	115.59
	GA+		107.77	111.36	106.10	108.30	112.82
E	GA-		66.07	85.05	72.23	74.26	90.19
	GA+		99.42	100.50	105.24	101.17	105.60
F	GA-		100.66	104.52	105.77	104.88	106.12
	GA+		95.54	102.08	106.28	102.49	108.31
G	GA-		120.77	124.84	120.98	113.32	123.16
	GA+		102.44	101.01	101.21	105.96	108.76
H	GA-		52.66	56.53	55.92	53.30	54.09
	GA+		101.85	96.10	100.77	102.34	101.58

Geno	GA	Flower	6	7	8	9	10
A	GA-		112.10	112.62	113.46	111.90	124.95
	GA+		108.39	111.16	108.39	105.96	119.73
B	GA-		106.12	104.39	107.76	109.58	107.90
	GA+		108.43	103.10	106.34	109.27	122.15
C	GA-		120.91	119.74	122.82	125.09	127.21
	GA+		113.10	113.00	116.19	114.64	112.56
D	GA-		114.03	122.58	117.00	121.92	119.11
	GA+		111.27	107.13	109.65	102.78	114.88
E	GA-		103.76	105.84	111.79	113.67	118.08
	GA+		111.49	108.43	102.10	111.49	110.04
F	GA-		110.58	107.21	104.76	108.83	109.86
	GA+		107.60	107.03	113.59	108.31	111.78
G	GA-		118.37	119.70	121.58	117.08	134.26
	GA+		105.30	102.32	106.47	110.46	114.03
H	GA-		52.54	60.34	58.75	62.62	57.22
	GA+		100.26	103.38	110.80	107.07	109.30

Geno	GA	Flower	15
A	GA-		121.29
	GA+		103.36
B	GA-		110.80
	GA+		114.95
C	GA-		131.29
	GA+		115.64
D	GA-		123.28
	GA+		111.15
E	GA-		117.80
	GA+		109.42
F	GA-		118.95
	GA+		115.82
G	GA-		126.75
	GA+		113.50
H	GA-		58.20
	GA+		115.32

SED (means with same flower) = 5.699 on 472 df; LSD (5%) = 11.199
SED (means with same genotype) = 5.738 on 506 df; LSD (5%) = 11.273
SED (all other comparisons) = 5.707 on 462 df; LSD (5%) = 11.215

2b. Transformed means for pistil length (Fig. 2r) on the square root scale used in ANOVA, with associated SED, df and LSD (5%) values.

Geno	GA	Flower	1	2	3	4	5
A	GA-		1.5273	1.5510	1.5624	1.5446	1.4986
	GA+		1.6030	1.5354	1.5434	1.5300	1.5495
B	GA-		1.5948	1.5884	1.6186	1.5766	1.5904
	GA+		1.5684	1.5479	1.5329	1.5298	1.5394
C	GA-		1.5369	1.4243	1.4466	1.4533	1.4433
	GA+		1.5107	1.4767	1.5145	1.5030	1.5446
D	GA-		1.5216	1.5022	1.5188	1.5440	1.5004
	GA+		1.5105	1.4755	1.5771	1.5753	1.5068
E	GA-		1.2140	1.2154	1.2359	1.2986	1.3231
	GA+		1.5047	1.5437	1.5306	1.5299	1.5149
F	GA-		1.6296	1.6117	1.6083	1.6128	1.6127
	GA+		1.5953	1.5772	1.5817	1.6055	1.5267
G	GA-		1.4343	1.3925	1.3873	1.4350	1.4750
	GA+		1.5363	1.5897	1.5611	1.5320	1.5199
H	GA-		1.0429	1.0376	1.0988	1.0847	1.1156
	GA+		1.5856	1.5201	1.5490	1.5548	1.5230

Geno	GA	Flower	6	7	8	9	10
A	GA-		1.4975	1.5277	1.4917	1.5475	1.5330
	GA+		1.5452	1.5633	1.5770	1.5199	1.4327
B	GA-		1.6032	1.6331	1.5987	1.6027	1.6083
	GA+		1.5924	1.5202	1.4994	1.5453	1.4504
C	GA-		1.4716	1.4764	1.4772	1.4539	1.5296
	GA+		1.5329	1.5638	1.4607	1.4563	1.4837
D	GA-		1.5780	1.5006	1.4701	1.4996	1.4685
	GA+		1.5423	1.5770	1.5629	1.6047	1.4783
E	GA-		1.3136	1.3545	1.3193	1.3257	1.3142
	GA+		1.5032	1.5428	1.5206	1.5068	1.5137
F	GA-		1.6347	1.6989	1.6482	1.6692	1.6384
	GA+		1.5116	1.5877	1.5494	1.6135	1.5411
G	GA-		1.4580	1.4628	1.5417	1.5067	1.4680
	GA+		1.5604	1.6200	1.5885	1.5410	1.5162
H	GA-		1.1923	1.1506	1.1852	1.1974	1.2104
	GA+		1.5696	1.5127	1.4966	1.4986	1.5086

Geno	GA	Flower	15
A	GA-		1.5544
	GA+		1.5610
B	GA-		1.6291
	GA+		1.4907
C	GA-		1.4763
	GA+		1.4756
D	GA-		1.4754
	GA+		1.5252
E	GA-		1.3738
	GA+		1.5384
F	GA-		1.6182
	GA+		1.5118
G	GA-		1.5241
	GA+		1.4958
H	GA-		1.1774
	GA+		1.4695

SED (means with same flower) = 0.03639 on 364 df; LSD (5%) = 0.07157
SED (means with same genotype) = 0.03553 on 510 df; LSD (5%) = 0.06981
SED (all other comparisons) = 0.03630 on 384 df; LSD (5%) = 0.07137

2c. Transformed means for stamen length (Fig. 2s) on the square root scale used in ANOVA, with associated SED, df and LSD (5%) values.

Geno	GA	Flower	1	2	3	4	5
A	GA-		1.5335	1.5985	1.6178	1.6163	1.5567
	GA+		1.6051	1.5699	1.5885	1.6040	1.6284
B	GA-		1.5376	1.5372	1.6099	1.5617	1.6616
	GA+		1.5708	1.5517	1.5588	1.5966	1.5411
C	GA-		1.5583	1.5516	1.5963	1.5919	1.6003
	GA+		1.5694	1.5410	1.5625	1.6134	1.6151
D	GA-		1.5426	1.5485	1.6162	1.6430	1.6126
	GA+		1.5658	1.5561	1.6224	1.6387	1.5966
E	GA-		0.9828	1.1093	1.0391	1.1113	1.2542
	GA+		1.5001	1.5457	1.5699	1.5382	1.5516
F	GA-		1.6349	1.6448	1.6536	1.6508	1.6608
	GA+		1.5597	1.5928	1.6289	1.6224	1.5886
G	GA-		1.5752	1.5556	1.5252	1.5244	1.6370
	GA+		1.5552	1.5970	1.5698	1.5768	1.5844
H	GA-		0.7542	0.7792	0.8196	0.7906	0.8196
	GA+		1.5991	1.4905	1.5528	1.5726	1.5342

Geno	GA	Flower	6	7	8	9	10
A	GA-		1.5832	1.6204	1.5893	1.6368	1.7136
	GA+		1.6079	1.6471	1.6538	1.5644	1.5660
B	GA-		1.6516	1.6677	1.6588	1.6779	1.6688
	GA+		1.6578	1.5385	1.5435	1.6125	1.6118
C	GA-		1.6182	1.6155	1.6369	1.6257	1.7241
	GA+		1.6295	1.6623	1.5738	1.5610	1.5714
D	GA-		1.6847	1.6615	1.5899	1.6558	1.6024
	GA+		1.6259	1.6313	1.6335	1.6224	1.5838
E	GA-		1.3366	1.3930	1.3951	1.4114	1.4250
	GA+		1.5856	1.6067	1.5361	1.5889	1.5866
F	GA-		1.7178	1.7585	1.6869	1.7410	1.7174
	GA+		1.5682	1.6422	1.6468	1.6759	1.6254
G	GA-		1.5852	1.6003	1.6984	1.6318	1.7009
	GA+		1.6003	1.6381	1.6345	1.6189	1.6166
H	GA-		0.8626	0.8936	0.9075	0.9480	0.9152
	GA+		1.5762	1.5380	1.5748	1.5492	1.5767

Geno	GA	Flower	15
A	GA-		1.7106
	GA+		1.5868
B	GA-		1.7146
	GA+		1.5967
C	GA-		1.6902
	GA+		1.5851
D	GA-		1.6376
	GA+		1.6057
E	GA-		1.4908
	GA+		1.6060
F	GA-		1.7631
	GA+		1.6239
G	GA-		1.7155
	GA+		1.5930
H	GA-		0.8942
	GA+		1.5768

SED (means with same flower) = 0.04754 on 421 df; LSD (5%) = 0.09344
SED (means with same genotype) = 0.04716 on 507 df; LSD (5%) = 0.09266
SED (all other comparisons) = 0.04723 on 445 df; LSD (5%) = 0.09281

2d. Means for petal length (Fig. 2t) used in ANOVA, with associated SED, df and LSD (5%) values.

Geno	GA	Flower	1	2	3	4	5
A	GA-		3.029	3.268	3.327	3.505	3.055
	GA+		3.458	3.089	3.271	3.141	3.161
B	GA-		3.107	3.133	3.387	3.144	3.578
	GA+		3.276	3.054	3.176	2.922	2.776
C	GA-		3.217	3.147	3.235	3.191	3.212
	GA+		3.218	2.965	3.050	3.080	3.193
D	GA-		3.056	2.919	3.453	3.394	3.177
	GA+		3.130	3.135	3.362	3.435	2.943
E	GA-		1.440	1.649	1.461	1.816	2.130
	GA+		3.235	3.161	3.325	3.106	3.093
F	GA-		3.333	3.386	3.387	3.435	3.484
	GA+		3.361	3.452	3.385	3.191	2.873
G	GA-		3.140	3.074	2.940	3.092	3.602
	GA+		3.146	3.417	3.315	3.150	3.289
H	GA-		0.850	0.841	0.920	0.868	0.852
	GA+		3.336	3.013	2.997	3.147	2.926

Geno	GA	Flower	6	7	8	9	10
A	GA-		3.104	3.209	3.076	3.487	3.596
	GA+		3.298	3.243	3.348	3.121	2.707
B	GA-		3.501	3.499	3.399	3.498	3.536
	GA+		3.197	2.720	2.952	3.067	2.755
C	GA-		3.219	3.249	3.332	3.161	3.681
	GA+		3.176	3.449	3.040	2.913	3.021
D	GA-		3.700	3.452	3.149	3.346	3.207
	GA+		3.380	3.347	3.189	3.433	2.944
E	GA-		2.237	2.360	2.423	2.383	2.571
	GA+		3.211	3.310	3.195	3.239	3.283
F	GA-		3.856	4.103	3.777	3.882	3.849
	GA+		3.066	3.340	3.300	3.508	3.221
G	GA-		3.169	3.126	3.652	3.444	3.460
	GA+		3.094	3.447	3.373	3.184	3.123
H	GA-		1.135	1.219	1.492	1.428	1.278
	GA+		3.099	3.049	2.935	2.985	3.190

Geno	GA	Flower	15
A	GA-		3.596
	GA+		2.970
B	GA-		3.807
	GA+		2.960
C	GA-		3.312
	GA+		2.912
D	GA-		3.163
	GA+		3.023
E	GA-		2.773
	GA+		3.213
F	GA-		3.580
	GA+		3.103
G	GA-		3.661
	GA+		3.099
H	GA-		1.129
	GA+		3.051

SED (means with same flower) = 0.2020 on 281 df; LSD (5%) = 0.3975
SED (means with same genotype) = 0.1923 on 510 df; LSD (5%) = 0.3778
SED (all other comparisons) = 0.1998 on 384 df; LSD (5%) = 0.3931

2e. Means for sepal length (Fig. 2u, 2v) used in ANOVA, with associated SED, df and LSD (5%) values.

Geno	GA	GA-	GA+
A		2.4228	2.5557
B		2.6163	2.5239
C		2.3075	2.5010
D		2.4254	2.6424
E		2.1046	2.5901
F		2.6622	2.6029
G		2.2660	2.6503
H		2.0051	2.6155

SED (means with same GA level) = 0.04859 on 14 df; LSD (5%) = 0.10422
SED (all other comparisons) = 0.04584 on 14 df; LSD (5%) = 0.09805

GA	Flower	1	2	3	4	5	6
GA-		2.3666	2.3343	2.3696	2.3938	2.3387	2.3285
GA+		2.8265	2.6690	2.7145	2.5931	2.5588	2.5456

GA	Flower	7	8	9	10	15
GA-		2.3679	2.3275	2.3624	2.3286	2.3457
GA+		2.5734	2.5252	2.5079	2.4743	2.4490

SED (means with same GA level) = 0.03889 on 511 df; LSD (5%) = 0.07640
SED (all other comparisons) = 0.03755 on 401 df; LSD (5%) = 0.07382

Genotypes are A (Wild Type Col-0), B (*ga20ox1*), C (*ga20ox2*), D (*ga20ox3*), E (*ga20ox1 ga20ox2*), F (*ga20ox1 ga20ox3*), G (*ga20ox2 ga20ox3*), H (*ga20ox1 ga20ox2 ga20ox3*).
