# Supplementary material for: The early inflorescence of Arabidopsis thaliana demonstrates positional effects in floral organ growth and meristem patterning
Source: Plant Reprod. 2017 Dec 20;31(2):171–91. doi: 10.1007/s00497-017-0320-3 (PMC5940708; doi:10.1007/s00497-017-0320-3)
Supplement: Supplementary file 3 — Supplementary material 3 (PDF 314 kb) [file 497_2017_320_MOESM3_ESM.pdf]

ONLINE RESOURCE 3: Predicted means and LSD values (5%) of the frequency of floral abnormalities during early flowering.

Article Title: The early inflorescence of *Arabidopsis thaliana* demonstrates positional effects in floral organ growth and meristem patterning

Journal: Plant Reproduction

Authors: ARG Plackett, SJ Powers, AL Phillips, ZA Wilson, P Hedden, SG Thomas

Corresponding author: ARG Plackett
Address: University of Cambridge, Department of Plant Sciences, Downing Street, Cambridge, CB2 3EA, UK
E-mail: arp74@cam.ac.uk

3a. Predicted mean frequencies of floral abnormalities (averaged across all flower positions), standard error (S.E.) and LSD (5%) values for comparisons, arising from the significant interaction between genotype and GA treatment (p = 0.024, Fig. 4a).

	GA-		GA+	
	Prediction	s.e.	Prediction	s.e.
Geno				
A	0.2267	0.07172	0.4086	0.09631
B	0.0456	0.03219	0.5204	0.11456
C	0.2498	0.07539	0.8401	0.13816
D	0.2041	0.06803	0.6810	0.12433
E	0.1598	0.06031	0.7960	0.13444
F	0.5002	0.10665	0.8176	0.13624
G	0.2488	0.07513	0.6805	0.12423
H	0.0676	0.03922	0.8189	0.13638

Least significant differences of predictions (5% level) (526 df)

Geno A GA GA-	1	*				
Geno A GA GA+	2	0.2359	*			
Geno B GA GA-	3	0.1544	0.1995	*		
Geno B GA GA+	4	0.2655	0.2940	0.2338	*	
Geno C GA GA-	5	0.2044	0.2403	0.1610	0.2694	*
Geno C GA GA+	6	0.3058	0.3308	0.2787	0.3526	0.3092
Geno D GA GA-	7	0.1942	0.2316	0.1478	0.2617	0.1995
Geno D GA GA+	8	0.2820	0.3090	0.2523	0.3321	0.2856
Geno E GA GA-	9	0.1841	0.2232	0.1343	0.2543	0.1897
Geno E GA GA+	10	0.2993	0.3249	0.2716	0.3470	0.3028
Geno F GA GA-	11	0.2525	0.2823	0.2189	0.3075	0.2566
Geno F GA GA+	12	0.3025	0.3278	0.2750	0.3497	0.3059
Geno G GA GA-	13	0.2041	0.2400	0.1606	0.2691	0.2091
Geno G GA GA+	14	0.2818	0.3088	0.2521	0.3320	0.2855
Geno H GA GA-	15	0.1606	0.2043	0.0997	0.2379	0.1669
Geno H GA GA+	16	0.3027	0.3280	0.2753	0.3499	0.3061
		1	2	3	4	5

Geno C GA GA+	6	*				
Geno D GA GA-	7	0.3025	*			
Geno D GA GA+	8	0.3651	0.2784	*		
Geno E GA GA-	9	0.2961	0.1786	0.2715	*	
Geno E GA GA+	10	0.3787	0.2960	0.3597	0.2895	*
Geno F GA GA-	11	0.3429	0.2485	0.3218	0.2407	0.3371
Geno F GA GA+	12	0.3812	0.2991	0.3623	0.2927	0.3760
Geno G GA GA-	13	0.3090	0.1991	0.2854	0.1893	0.3026
Geno G GA GA+	14	0.3650	0.2782	0.3453	0.2713	0.3596
Geno H GA GA-	15	0.2821	0.1543	0.2561	0.1413	0.2751
Geno H GA GA+	16	0.3814	0.2994	0.3625	0.2929	0.3762
		6	7	8	9	10

Geno F GA GA-	11	*				
Geno F GA GA+	12	0.3399	*			
Geno G GA GA-	13	0.2563	0.3056	*		
Geno G GA GA+	14	0.3216	0.3622	0.2852	*	
Geno H GA GA-	15	0.2232	0.2785	0.1665	0.2559	*
Geno H GA GA+	16	0.3401	0.3787	0.3059	0.3624	0.2788
		11	12	13	14	15

Genotypes are: A (Wild Type Col-0), B (*ga20ox1*), C (*ga20ox2*), D (*ga20ox3*), E (*ga20ox1 ga20ox2*), F (*ga20ox1 ga20ox3*), G (*ga20ox2 ga20ox3*), H (*ga20ox1 ga20ox2 ga20ox3*).

3b. Predicted mean frequencies of floral abnormalities (averaged across control growth conditions and GA-treatment) ± S.E. and LSD (5%) values for comparison, arising from the significant interaction between genotype and inflorescence position ($p = 0.033$). Asterisks denote a significant difference ($p < 0.05$) from wild type at that flower position. Comparisons between inflorescence positions within a genotype are not shown.

	Genotype							
Flower Position	Wild type (Col-0)	*ga20ox1*	*ga20ox2*	*ga20ox3*	*ga20ox1 ga20ox2*	*ga20ox1 ga20ox3*	*ga20ox2 ga20ox3*	*ga20ox1 ga20ox2 ga20ox3*
1	**1.1267** ±0.3754	**0.1261*** ±0.1255	**0.8759** ±0.3309	**1.0020** ±0.3504	**0.8769** ±0.3310	**1.0006** ±0.3537	**0.2500*** ±0.1768	**0.6236** ±0.2790
2	**0.0000** ±0.0010	**0.5000*** ±0.2500	**0.7478*** ±0.3059	**0.5017*** ±0.2503	**0.8757*** ±0.3308	**1.0010*** ±0.3537	**0.9975*** ±0.3531	**0.6245*** ±0.2793
3	**0.5018** ±0.2505	**0.0000*** ±0.0010	**0.3738** ±0.2160	**0.1239** ±0.1245	**0.1265** ±0.1258	**0.7486** ±0.3059	**0.3765** ±0.2170	**0.0000*** ±0.0010
4	**0.1261** ±0.1255	**0.2515** ±0.1772	**0.1261** ±0.1255	**0.2497** ±0.1765	**0.1261** ±0.1255	**0.2500** ±0.1768	**0.4977** ±0.2493	**0.0000** ±0.0010
5	**0.1261** ±0.1255	**0.0000** ±0.0010	**0.7466** ±0.3054	**0.1236** ±0.1242	**0.6270** ±0.2797	**0.5006** ±0.2501	**0.0000** ±0.0010	**0.3752** ±0.4914
6	**0.1236** ±0.1242	**0.0000** ±0.0010	**0.2479** ±0.1759	**0.4993** ±0.2496	**0.3752** ±0.2164	**0.2526** ±0.1777	**0.3718** ±0.2155	**0.1261** ±0.1255
7	**0.1236** ±0.1242	**0.0000** ±0.0010	**0.2515** ±0.1772	**0.4968** ±0.2490	**0.1261** ±0.1255	**0.6241** ±0.2793	**0.1236** ±0.1242	**0.8752*** ±0.3305
8	**0.0000** ±0.0010	**0.3723** ±0.2156	**0.7500*** ±0.3060	**0.2479** ±0.1759	**0.2475** ±0.7590	**0.1236** ±0.1242	**0.3752** ±0.2164	**0.1261** ±0.1255
9	**0.2479** ±0.1759	**0.1236** ±0.1242	**0.6236** ±0.2790	**0.6247** ±0.2792	**0.6247** ±0.2792	**0.7510** ±0.3063	**0.3752** ±0.2164	**0.6258** ±0.2796
10	**0.4981** ±0.2493	**0.7469** ±0.3053	**0.7490** ±0.3058	**0.1261** ±0.1255	**0.6236** ±0.2790	**1.1248** ±0.3749	**0.6258** ±0.2795	**0.8739** ±0.3303
15	**0.6238** ±0.2792	**0.9998** ±0.4080	**0.4968** ±0.2490	**0.8751** ±0.3306	**0.6247** ±0.6247	**0.8718** ±0.3299	**1.1246** ±0.3748	**0.6224** ±0.2787

Least significant differences of predictions (5% level)

Geno A FlowerPosn 1	1	*			
Geno A FlowerPosn 2	2	0.7374	*		
Geno A FlowerPosn 3	3	0.8865	0.4920	*	
Geno A FlowerPosn 4	4	0.7775	0.2465	0.5503	*
Geno A FlowerPosn 5	5	0.7775	0.2465	0.5503	0.3486
Geno A FlowerPosn 6	6	0.7767	0.2440	0.5492	0.3468
Geno A FlowerPosn 7	7	0.7767	0.2440	0.5492	0.3468
Geno A FlowerPosn 8	8	0.7374	0.0027	0.4920	0.2465
Geno A FlowerPosn 9	9	0.8144	0.3455	0.6012	0.4244
Geno A FlowerPosn 10	10	0.8853	0.4898	0.6942	0.5483
Geno A FlowerPosn 15	11	0.9190	0.5485	0.7368	0.6013
Geno B FlowerPosn 1	12	0.7775	0.2465	0.5503	0.3486
Geno B FlowerPosn 2	13	0.8860	0.4911	0.6952	0.5495
Geno B FlowerPosn 3	14	0.7374	0.0027	0.4920	0.2465
Geno B FlowerPosn 4	15	0.8154	0.3480	0.6027	0.4265
Geno B FlowerPosn 5	16	0.7374	0.0027	0.4920	0.2465
Geno B FlowerPosn 6	17	0.7374	0.0027	0.4920	0.2465
Geno B FlowerPosn 7	18	0.7374	0.0027	0.4920	0.2465
Geno B FlowerPosn 8	19	0.8504	0.4235	0.6492	0.4900
Geno B FlowerPosn 9	20	0.7767	0.2440	0.5492	0.3468
Geno B FlowerPosn 10	21	0.9506	0.5998	0.7758	0.6485
Geno B FlowerPosn 15	22	1.0891	0.8015	0.9405	0.8386
Geno C FlowerPosn 1	23	0.9831	0.6501	0.8153	0.6953
Geno C FlowerPosn 2	24	0.9512	0.6009	0.7766	0.6495
Geno C FlowerPosn 3	25	0.8508	0.4243	0.6497	0.4907
Geno C FlowerPosn 4	26	0.7775	0.2465	0.5503	0.3486
Geno C FlowerPosn 5	27	0.9507	0.6000	0.7759	0.6486
Geno C FlowerPosn 6	28	0.8144	0.3455	0.6012	0.4244
Geno C FlowerPosn 7	29	0.8154	0.3480	0.6027	0.4265
Geno C FlowerPosn 8	30	0.9513	0.6011	0.7768	0.6496
Geno C FlowerPosn 9	31	0.9188	0.5481	0.7365	0.6009
Geno C FlowerPosn 10	32	0.9511	0.6006	0.7764	0.6492
Geno C FlowerPosn 15	33	0.8849	0.4892	0.6938	0.5478
Geno D FlowerPosn 1	34	1.0136	0.6954	0.8518	0.7378
Geno D FlowerPosn 2	35	0.8863	0.4918	0.6956	0.5501
Geno D FlowerPosn 3	36	0.7769	0.2447	0.5495	0.3473
Geno D FlowerPosn 4	37	0.8149	0.3468	0.6019	0.4254
Geno D FlowerPosn 5	38	0.7767	0.2440	0.5492	0.3468
Geno D FlowerPosn 6	39	0.8856	0.4904	0.6947	0.5489
Geno D FlowerPosn 7	40	0.8849	0.4892	0.6938	0.5478
Geno D FlowerPosn 8	41	0.8144	0.3455	0.6012	0.4244
Geno D FlowerPosn 9	42	0.9191	0.5486	0.7369	0.6014
Geno D FlowerPosn 10	43	0.7775	0.2465	0.5503	0.3486
Geno D FlowerPosn 15	44	0.9827	0.6495	0.8148	0.6947
Geno E FlowerPosn 1	45	0.9832	0.6503	0.8155	0.6954
Geno E FlowerPosn 2	46	0.9828	0.6498	0.8150	0.6949
Geno E FlowerPosn 3	47	0.7777	0.2472	0.5506	0.3490
Geno E FlowerPosn 4	48	0.7775	0.2465	0.5503	0.3486
Geno E FlowerPosn 5	49	0.9197	0.5496	0.7376	0.6023
Geno E FlowerPosn 6	50	0.8512	0.4251	0.6502	0.4914
Geno E FlowerPosn 7	51	0.7775	0.2465	0.5503	0.3486
Geno E FlowerPosn 8	52	0.8143	0.3455	0.6012	0.4244
Geno E FlowerPosn 9	53	0.9191	0.5486	0.7369	0.6014
Geno E FlowerPosn 10	54	0.9188	0.5481	0.7365	0.6009
Geno E FlowerPosn 15	55	0.9191	0.5486	0.7369	0.6014
Geno F FlowerPosn 1	56	1.0132	0.6949	0.8514	0.7373
Geno F FlowerPosn 2	57	1.0131	0.6948	0.8513	0.7372
Geno F FlowerPosn 3	58	0.9512	0.6009	0.7766	0.6495
Geno F FlowerPosn 4	59	0.8151	0.3473	0.6022	0.4259
Geno F FlowerPosn 5	60	0.8860	0.4912	0.6953	0.5496
Geno F FlowerPosn 6	61	0.8158	0.3490	0.6032	0.4273
Geno F FlowerPosn 7	62	0.9191	0.5486	0.7369	0.6014
Geno F FlowerPosn 8	63	0.7767	0.2440	0.5492	0.3468
Geno F FlowerPosn 9	64	0.9518	0.6017	0.7773	0.6502
Geno F FlowerPosn 10	65	1.0422	0.7364	0.8857	0.7766
Geno F FlowerPosn 15	66	0.9818	0.6482	0.8138	0.6935

Geno G FlowerPosn 1	67	0.8151	0.3473	0.6022	0.4258
Geno G FlowerPosn 2	68	1.0124	0.6937	0.8504	0.7362
Geno G FlowerPosn 3	69	0.8517	0.4263	0.6510	0.4924
Geno G FlowerPosn 4	70	0.8852	0.4898	0.6942	0.5483
Geno G FlowerPosn 5	71	0.7374	0.0027	0.4920	0.2465
Geno G FlowerPosn 6	72	0.8503	0.4234	0.6491	0.4899
Geno G FlowerPosn 7	73	0.7767	0.2440	0.5492	0.3468
Geno G FlowerPosn 8	74	0.8512	0.4251	0.6502	0.4914
Geno G FlowerPosn 9	75	0.8512	0.4251	0.6502	0.4914
Geno G FlowerPosn 10	76	0.9194	0.5491	0.7373	0.6018
Geno G FlowerPosn 15	77	1.0420	0.7363	0.8855	0.7764
Geno H FlowerPosn 1	78	0.9188	0.5481	0.7365	0.6009
Geno H FlowerPosn 2	79	0.9191	0.5486	0.7369	0.6014
Geno H FlowerPosn 3	80	0.7374	0.0027	0.4920	0.2465
Geno H FlowerPosn 4	81	0.7374	0.0027	0.4920	0.2465
Geno H FlowerPosn 5	82	0.8512	0.4251	0.6502	0.4914
Geno H FlowerPosn 6	83	0.7775	0.2465	0.5503	0.3486
Geno H FlowerPosn 7	84	0.9825	0.6493	0.8147	0.6945
Geno H FlowerPosn 8	85	0.7775	0.2465	0.5503	0.3486
Geno H FlowerPosn 9	86	0.9194	0.5492	0.7373	0.6019
Geno H FlowerPosn 10	87	0.9823	0.6489	0.8144	0.6942
Geno H FlowerPosn 15	88	0.9185	0.5476	0.7361	0.6005
		1	2	3	4

Geno A FlowerPosn 5	5	*			
Geno A FlowerPosn 6	6	0.3468	*		
Geno A FlowerPosn 7	7	0.3468	0.3450	*	
Geno A FlowerPosn 8	8	0.2465	0.2440	0.2440	*
Geno A FlowerPosn 9	9	0.4244	0.4230	0.4230	0.3455
Geno A FlowerPosn 10	10	0.5483	0.5472	0.5472	0.4898
Geno A FlowerPosn 15	11	0.6013	0.6003	0.6003	0.5485
Geno B FlowerPosn 1	12	0.3486	0.3468	0.3468	0.2465
Geno B FlowerPosn 2	13	0.5495	0.5484	0.5484	0.4911
Geno B FlowerPosn 3	14	0.2465	0.2440	0.2440	0.0027
Geno B FlowerPosn 4	15	0.4265	0.4250	0.4250	0.3480
Geno B FlowerPosn 5	16	0.2465	0.2440	0.2440	0.0027
Geno B FlowerPosn 6	17	0.2465	0.2440	0.2440	0.0027
Geno B FlowerPosn 7	18	0.2465	0.2440	0.2440	0.0027
Geno B FlowerPosn 8	19	0.4900	0.4887	0.4887	0.4235
Geno B FlowerPosn 9	20	0.3468	0.3450	0.3450	0.2440
Geno B FlowerPosn 10	21	0.6485	0.6475	0.6475	0.5998
Geno B FlowerPosn 15	22	0.8386	0.8378	0.8378	0.8015
Geno C FlowerPosn 1	23	0.6953	0.6944	0.6944	0.6501
Geno C FlowerPosn 2	24	0.6495	0.6485	0.6485	0.6009
Geno C FlowerPosn 3	25	0.4907	0.4894	0.4894	0.4243
Geno C FlowerPosn 4	26	0.3486	0.3468	0.3468	0.2465
Geno C FlowerPosn 5	27	0.6486	0.6477	0.6477	0.6000
Geno C FlowerPosn 6	28	0.4244	0.4230	0.4230	0.3455
Geno C FlowerPosn 7	29	0.4265	0.4250	0.4250	0.3480
Geno C FlowerPosn 8	30	0.6496	0.6487	0.6487	0.6011
Geno C FlowerPosn 9	31	0.6009	0.5999	0.5999	0.5481
Geno C FlowerPosn 10	32	0.6492	0.6483	0.6483	0.6006
Geno C FlowerPosn 15	33	0.5478	0.5466	0.5466	0.4892
Geno D FlowerPosn 1	34	0.7378	0.7369	0.7369	0.6954
Geno D FlowerPosn 2	35	0.5501	0.5490	0.5490	0.4918
Geno D FlowerPosn 3	36	0.3473	0.3455	0.3455	0.2447
Geno D FlowerPosn 4	37	0.4254	0.4240	0.4240	0.3468
Geno D FlowerPosn 5	38	0.3468	0.3450	0.3450	0.2440
Geno D FlowerPosn 6	39	0.5489	0.5478	0.5478	0.4904
Geno D FlowerPosn 7	40	0.5478	0.5466	0.5466	0.4892
Geno D FlowerPosn 8	41	0.4244	0.4230	0.4230	0.3455
Geno D FlowerPosn 9	42	0.6014	0.6004	0.6004	0.5486
Geno D FlowerPosn 10	43	0.3486	0.3468	0.3468	0.2465
Geno D FlowerPosn 15	44	0.6947	0.6938	0.6938	0.6495
Geno E FlowerPosn 1	45	0.6954	0.6946	0.6946	0.6503
Geno E FlowerPosn 2	46	0.6949	0.6940	0.6940	0.6498
Geno E FlowerPosn 3	47	0.3490	0.3473	0.3473	0.2472
Geno E FlowerPosn 4	48	0.3486	0.3468	0.3468	0.2465

Geno E FlowerPosn	5	49	0.6023	0.6013	0.6013	0.5496
Geno E FlowerPosn	6	50	0.4914	0.4902	0.4902	0.4251
Geno E FlowerPosn	7	51	0.3486	0.3468	0.3468	0.2465
Geno E FlowerPosn	8	52	0.4244	0.4230	0.4230	0.3455
Geno E FlowerPosn	9	53	0.6014	0.6004	0.6004	0.5486
Geno E FlowerPosn	10	54	0.6009	0.5999	0.5999	0.5481
Geno E FlowerPosn	15	55	0.6014	0.6004	0.6004	0.5486
Geno F FlowerPosn	1	56	0.7373	0.7364	0.7364	0.6949
Geno F FlowerPosn	2	57	0.7372	0.7364	0.7364	0.6948
Geno F FlowerPosn	3	58	0.6495	0.6485	0.6485	0.6009
Geno F FlowerPosn	4	59	0.4259	0.4244	0.4244	0.3473
Geno F FlowerPosn	5	60	0.5496	0.5485	0.5485	0.4912
Geno F FlowerPosn	6	61	0.4273	0.4259	0.4259	0.3490
Geno F FlowerPosn	7	62	0.6014	0.6004	0.6004	0.5486
Geno F FlowerPosn	8	63	0.3468	0.3450	0.3450	0.2440
Geno F FlowerPosn	9	64	0.6502	0.6493	0.6493	0.6017
Geno F FlowerPosn	10	65	0.7766	0.7758	0.7758	0.7364
Geno F FlowerPosn	15	66	0.6935	0.6926	0.6926	0.6482
Geno G FlowerPosn	1	67	0.4258	0.4244	0.4244	0.3473
Geno G FlowerPosn	2	68	0.7362	0.7353	0.7353	0.6937
Geno G FlowerPosn	3	69	0.4924	0.4911	0.4911	0.4263
Geno G FlowerPosn	4	70	0.5483	0.5472	0.5472	0.4898
Geno G FlowerPosn	5	71	0.2465	0.2440	0.2440	0.0027
Geno G FlowerPosn	6	72	0.4899	0.4886	0.4886	0.4234
Geno G FlowerPosn	7	73	0.3468	0.3450	0.3450	0.2440
Geno G FlowerPosn	8	74	0.4914	0.4902	0.4902	0.4251
Geno G FlowerPosn	9	75	0.4914	0.4902	0.4902	0.4251
Geno G FlowerPosn	10	76	0.6018	0.6008	0.6008	0.5491
Geno G FlowerPosn	15	77	0.7764	0.7756	0.7756	0.7363
Geno H FlowerPosn	1	78	0.6009	0.5999	0.5999	0.5481
Geno H FlowerPosn	2	79	0.6014	0.6004	0.6004	0.5486
Geno H FlowerPosn	3	80	0.2465	0.2440	0.2440	0.0027
Geno H FlowerPosn	4	81	0.2465	0.2440	0.2440	0.0027
Geno H FlowerPosn	5	82	0.4914	0.4902	0.4902	0.4251
Geno H FlowerPosn	6	83	0.3486	0.3468	0.3468	0.2465
Geno H FlowerPosn	7	84	0.6945	0.6936	0.6936	0.6493
Geno H FlowerPosn	8	85	0.3486	0.3468	0.3468	0.2465
Geno H FlowerPosn	9	86	0.6019	0.6009	0.6009	0.5492
Geno H FlowerPosn	10	87	0.6942	0.6933	0.6933	0.6489
Geno H FlowerPosn	15	88	0.6005	0.5995	0.5995	0.5476

			5	6	7	8
Geno A FlowerPosn	9	9	*			
Geno A FlowerPosn	10	10	0.5994	*		
Geno A FlowerPosn	15	11	0.6482	0.7353	*	
Geno B FlowerPosn	1	12	0.4244	0.5483	0.6013	*
Geno B FlowerPosn	2	13	0.6005	0.6936	0.7362	0.5495
Geno B FlowerPosn	3	14	0.3455	0.4898	0.5485	0.2465
Geno B FlowerPosn	4	15	0.4904	0.6009	0.6496	0.4265
Geno B FlowerPosn	5	16	0.3455	0.4898	0.5485	0.2465
Geno B FlowerPosn	6	17	0.3455	0.4898	0.5485	0.2465
Geno B FlowerPosn	7	18	0.3455	0.4898	0.5485	0.2465
Geno B FlowerPosn	8	19	0.5466	0.6475	0.6929	0.4900
Geno B FlowerPosn	9	20	0.4230	0.5472	0.6003	0.3468
Geno B FlowerPosn	10	21	0.6922	0.7744	0.8128	0.6485
Geno B FlowerPosn	15	22	0.8728	0.9393	0.9712	0.8386
Geno C FlowerPosn	1	23	0.7362	0.8140	0.8506	0.6953
Geno C FlowerPosn	2	24	0.6931	0.7752	0.8136	0.6495
Geno C FlowerPosn	3	25	0.5472	0.6480	0.6934	0.4907
Geno C FlowerPosn	4	26	0.4244	0.5483	0.6013	0.3486
Geno C FlowerPosn	5	27	0.6924	0.7745	0.8129	0.6486
Geno C FlowerPosn	6	28	0.4887	0.5994	0.6482	0.4244
Geno C FlowerPosn	7	29	0.4904	0.6009	0.6496	0.4265
Geno C FlowerPosn	8	30	0.6933	0.7754	0.8137	0.6496
Geno C FlowerPosn	9	31	0.6479	0.7350	0.7754	0.6009
Geno C FlowerPosn	10	32	0.6929	0.7750	0.8134	0.6492
Geno C FlowerPosn	15	33	0.5989	0.6922	0.7349	0.5478
Geno D FlowerPosn	1	34	0.7765	0.8506	0.8856	0.7378

Geno D FlowerPosn	2	35	0.6010	0.6941	0.7366	0.5501
Geno D FlowerPosn	3	36	0.4234	0.5475	0.6006	0.3473
Geno D FlowerPosn	4	37	0.4895	0.6001	0.6489	0.4254
Geno D FlowerPosn	5	38	0.4230	0.5472	0.6003	0.3468
Geno D FlowerPosn	6	39	0.5999	0.6931	0.7358	0.5489
Geno D FlowerPosn	7	40	0.5989	0.6922	0.7349	0.5478
Geno D FlowerPosn	8	41	0.4887	0.5994	0.6482	0.4244
Geno D FlowerPosn	9	42	0.6483	0.7354	0.7757	0.6014
Geno D FlowerPosn	10	43	0.4244	0.5483	0.6013	0.3486
Geno D FlowerPosn	15	44	0.7357	0.8135	0.8501	0.6947
Geno E FlowerPosn	1	45	0.7364	0.8141	0.8507	0.6954
Geno E FlowerPosn	2	46	0.7359	0.8137	0.8503	0.6949
Geno E FlowerPosn	3	47	0.4248	0.5486	0.6016	0.3490
Geno E FlowerPosn	4	48	0.4244	0.5483	0.6013	0.3486
Geno E FlowerPosn	5	49	0.6492	0.7362	0.7764	0.6023
Geno E FlowerPosn	6	50	0.5478	0.6486	0.6939	0.4914
Geno E FlowerPosn	7	51	0.4244	0.5483	0.6013	0.3486
Geno E FlowerPosn	8	52	0.4886	0.5994	0.6482	0.4244
Geno E FlowerPosn	9	53	0.6483	0.7354	0.7757	0.6014
Geno E FlowerPosn	10	54	0.6479	0.7350	0.7754	0.6009
Geno E FlowerPosn	15	55	0.6483	0.7354	0.7757	0.6014
Geno F FlowerPosn	1	56	0.7760	0.8501	0.8852	0.7373
Geno F FlowerPosn	2	57	0.7759	0.8501	0.8852	0.7372
Geno F FlowerPosn	3	58	0.6932	0.7752	0.8136	0.6495
Geno F FlowerPosn	4	59	0.4899	0.6004	0.6492	0.4259
Geno F FlowerPosn	5	60	0.6006	0.6937	0.7363	0.5496
Geno F FlowerPosn	6	61	0.4911	0.6014	0.6501	0.4273
Geno F FlowerPosn	7	62	0.6484	0.7355	0.7758	0.6014
Geno F FlowerPosn	8	63	0.4230	0.5472	0.6003	0.3468
Geno F FlowerPosn	9	64	0.6939	0.7759	0.8142	0.6502
Geno F FlowerPosn	10	65	0.8135	0.8844	0.9182	0.7766
Geno F FlowerPosn	15	66	0.7345	0.8124	0.8491	0.6935
Geno G FlowerPosn	1	67	0.4899	0.6004	0.6492	0.4258
Geno G FlowerPosn	2	68	0.7750	0.8492	0.8843	0.7362
Geno G FlowerPosn	3	69	0.5487	0.6493	0.6946	0.4924
Geno G FlowerPosn	4	70	0.5994	0.6927	0.7353	0.5483
Geno G FlowerPosn	5	71	0.3455	0.4898	0.5485	0.2465
Geno G FlowerPosn	6	72	0.5465	0.6474	0.6929	0.4899
Geno G FlowerPosn	7	73	0.4230	0.5472	0.6003	0.3468
Geno G FlowerPosn	8	74	0.5478	0.6486	0.6939	0.4914
Geno G FlowerPosn	9	75	0.5478	0.6486	0.6939	0.4914
Geno G FlowerPosn	10	76	0.6487	0.7358	0.7761	0.6018
Geno G FlowerPosn	15	77	0.8133	0.8843	0.9181	0.7764
Geno H FlowerPosn	1	78	0.6479	0.7350	0.7754	0.6009
Geno H FlowerPosn	2	79	0.6484	0.7355	0.7758	0.6014
Geno H FlowerPosn	3	80	0.3455	0.4898	0.5485	0.2465
Geno H FlowerPosn	4	81	0.3455	0.4898	0.5485	0.2465
Geno H FlowerPosn	5	82	0.5478	0.6486	0.6939	0.4914
Geno H FlowerPosn	6	83	0.4244	0.5483	0.6013	0.3486
Geno H FlowerPosn	7	84	0.7355	0.8133	0.8499	0.6945
Geno H FlowerPosn	8	85	0.4244	0.5483	0.6013	0.3486
Geno H FlowerPosn	9	86	0.6488	0.7359	0.7762	0.6019
Geno H FlowerPosn	10	87	0.7352	0.8130	0.8497	0.6942
Geno H FlowerPosn	15	88	0.6475	0.7347	0.7750	0.6005

			9	10	11	12
Geno B FlowerPosn	2	13	*			
Geno B FlowerPosn	3	14	0.4911	*		
Geno B FlowerPosn	4	15	0.6019	0.3480	*	
Geno B FlowerPosn	5	16	0.4911	0.0027	0.3480	*
Geno B FlowerPosn	6	17	0.4911	0.0027	0.3480	0.0027
Geno B FlowerPosn	7	18	0.4911	0.0027	0.3480	0.0027
Geno B FlowerPosn	8	19	0.6485	0.4235	0.5481	0.4235
Geno B FlowerPosn	9	20	0.5484	0.2440	0.4250	0.2440
Geno B FlowerPosn	10	21	0.7752	0.5998	0.6935	0.5998
Geno B FlowerPosn	15	22	0.9400	0.8015	0.8738	0.8015
Geno C FlowerPosn	1	23	0.8148	0.6501	0.7374	0.6501
Geno C FlowerPosn	2	24	0.7761	0.6009	0.6944	0.6009

Geno C FlowerPosn 3	25	0.6490	0.4243	0.5488	0.4243
Geno C FlowerPosn 4	26	0.5495	0.2465	0.4265	0.2465
Geno C FlowerPosn 5	27	0.7754	0.6000	0.6936	0.6000
Geno C FlowerPosn 6	28	0.6005	0.3455	0.4904	0.3455
Geno C FlowerPosn 7	29	0.6019	0.3480	0.4922	0.3480
Geno C FlowerPosn 8	30	0.7762	0.6011	0.6946	0.6011
Geno C FlowerPosn 9	31	0.7359	0.5481	0.6492	0.5481
Geno C FlowerPosn 10	32	0.7759	0.6006	0.6942	0.6006
Geno C FlowerPosn 15	33	0.6932	0.4892	0.6003	0.4892
Geno D FlowerPosn 1	34	0.8513	0.6954	0.7776	0.6954
Geno D FlowerPosn 2	35	0.6950	0.4918	0.6025	0.4918
Geno D FlowerPosn 3	36	0.5487	0.2447	0.4254	0.2447
Geno D FlowerPosn 4	37	0.6012	0.3468	0.4913	0.3468
Geno D FlowerPosn 5	38	0.5484	0.2440	0.4250	0.2440
Geno D FlowerPosn 6	39	0.6941	0.4904	0.6014	0.4904
Geno D FlowerPosn 7	40	0.6932	0.4892	0.6003	0.4892
Geno D FlowerPosn 8	41	0.6005	0.3455	0.4904	0.3455
Geno D FlowerPosn 9	42	0.7363	0.5486	0.6497	0.5486
Geno D FlowerPosn 10	43	0.5495	0.2465	0.4265	0.2465
Geno D FlowerPosn 15	44	0.8143	0.6495	0.7369	0.6495
Geno E FlowerPosn 1	45	0.8149	0.6503	0.7376	0.6503
Geno E FlowerPosn 2	46	0.8145	0.6498	0.7371	0.6498
Geno E FlowerPosn 3	47	0.5498	0.2472	0.4269	0.2472
Geno E FlowerPosn 4	48	0.5495	0.2465	0.4265	0.2465
Geno E FlowerPosn 5	49	0.7370	0.5496	0.6505	0.5496
Geno E FlowerPosn 6	50	0.6496	0.4251	0.5494	0.4251
Geno E FlowerPosn 7	51	0.5495	0.2465	0.4265	0.2465
Geno E FlowerPosn 8	52	0.6005	0.3455	0.4904	0.3455
Geno E FlowerPosn 9	53	0.7363	0.5486	0.6497	0.5486
Geno E FlowerPosn 10	54	0.7359	0.5481	0.6492	0.5481
Geno E FlowerPosn 15	55	0.7363	0.5486	0.6497	0.5486
Geno F FlowerPosn 1	56	0.8509	0.6949	0.7771	0.6949
Geno F FlowerPosn 2	57	0.8508	0.6948	0.7771	0.6948
Geno F FlowerPosn 3	58	0.7761	0.6009	0.6944	0.6009
Geno F FlowerPosn 4	59	0.6015	0.3473	0.4917	0.3473
Geno F FlowerPosn 5	60	0.6946	0.4912	0.6020	0.4912
Geno F FlowerPosn 6	61	0.6025	0.3490	0.4929	0.3490
Geno F FlowerPosn 7	62	0.7363	0.5486	0.6497	0.5486
Geno F FlowerPosn 8	63	0.5484	0.2440	0.4250	0.2440
Geno F FlowerPosn 9	64	0.7767	0.6017	0.6951	0.6017
Geno F FlowerPosn 10	65	0.8852	0.7364	0.8145	0.7364
Geno F FlowerPosn 15	66	0.8132	0.6482	0.7357	0.6482
Geno G FlowerPosn 1	67	0.6015	0.3473	0.4917	0.3473
Geno G FlowerPosn 2	68	0.8499	0.6937	0.7761	0.6937
Geno G FlowerPosn 3	69	0.6503	0.4263	0.5503	0.4263
Geno G FlowerPosn 4	70	0.6936	0.4898	0.6008	0.4898
Geno G FlowerPosn 5	71	0.4911	0.0027	0.3480	0.0027
Geno G FlowerPosn 6	72	0.6484	0.4234	0.5481	0.4234
Geno G FlowerPosn 7	73	0.5484	0.2440	0.4250	0.2440
Geno G FlowerPosn 8	74	0.6496	0.4251	0.5494	0.4251
Geno G FlowerPosn 9	75	0.6496	0.4251	0.5494	0.4251
Geno G FlowerPosn 10	76	0.7367	0.5491	0.6501	0.5491
Geno G FlowerPosn 15	77	0.8850	0.7363	0.8144	0.7363
Geno H FlowerPosn 1	78	0.7359	0.5481	0.6492	0.5481
Geno H FlowerPosn 2	79	0.7363	0.5486	0.6497	0.5486
Geno H FlowerPosn 3	80	0.4911	0.0027	0.3480	0.0027
Geno H FlowerPosn 4	81	0.4911	0.0027	0.3480	0.0027
Geno H FlowerPosn 5	82	0.6496	0.4251	0.5494	0.4251
Geno H FlowerPosn 6	83	0.5495	0.2465	0.4265	0.2465
Geno H FlowerPosn 7	84	0.8141	0.6493	0.7367	0.6493
Geno H FlowerPosn 8	85	0.5495	0.2465	0.4265	0.2465
Geno H FlowerPosn 9	86	0.7368	0.5492	0.6502	0.5492
Geno H FlowerPosn 10	87	0.8138	0.6489	0.7364	0.6489
Geno H FlowerPosn 15	88	0.7355	0.5476	0.6488	0.5476
		13	14	15	16

Geno	B	FlowerPosn	6	17	*			
Geno	B	FlowerPosn	7	18	0.0027	*		
Geno	B	FlowerPosn	8	19	0.4235	0.4235	*	
Geno	B	FlowerPosn	9	20	0.2440	0.2440	0.4887	*
Geno	B	FlowerPosn	10	21	0.5998	0.5998	0.7342	0.6475
Geno	B	FlowerPosn	15	22	0.8015	0.8015	0.9065	0.8378
Geno	C	FlowerPosn	1	23	0.6501	0.6501	0.7759	0.6944
Geno	C	FlowerPosn	2	24	0.6009	0.6009	0.7351	0.6485
Geno	C	FlowerPosn	3	25	0.4243	0.4243	0.5995	0.4894
Geno	C	FlowerPosn	4	26	0.2465	0.2465	0.4900	0.3468
Geno	C	FlowerPosn	5	27	0.6000	0.6000	0.7344	0.6477
Geno	C	FlowerPosn	6	28	0.3455	0.3455	0.5466	0.4230
Geno	C	FlowerPosn	7	29	0.3480	0.3480	0.5481	0.4250
Geno	C	FlowerPosn	8	30	0.6011	0.6011	0.7353	0.6487
Geno	C	FlowerPosn	9	31	0.5481	0.5481	0.6926	0.5999
Geno	C	FlowerPosn	10	32	0.6006	0.6006	0.7349	0.6483
Geno	C	FlowerPosn	15	33	0.4892	0.4892	0.6470	0.5466
Geno	D	FlowerPosn	1	34	0.6954	0.6954	0.8142	0.7369
Geno	D	FlowerPosn	2	35	0.4918	0.4918	0.6490	0.5490
Geno	D	FlowerPosn	3	36	0.2447	0.2447	0.4891	0.3455
Geno	D	FlowerPosn	4	37	0.3468	0.3468	0.5473	0.4240
Geno	D	FlowerPosn	5	38	0.2440	0.2440	0.4887	0.3450
Geno	D	FlowerPosn	6	39	0.4904	0.4904	0.6480	0.5478
Geno	D	FlowerPosn	7	40	0.4892	0.4892	0.6470	0.5466
Geno	D	FlowerPosn	8	41	0.3455	0.3455	0.5466	0.4230
Geno	D	FlowerPosn	9	42	0.5486	0.5486	0.6930	0.6004
Geno	D	FlowerPosn	10	43	0.2465	0.2465	0.4900	0.3468
Geno	D	FlowerPosn	15	44	0.6495	0.6495	0.7754	0.6938
Geno	E	FlowerPosn	1	45	0.6503	0.6503	0.7760	0.6946
Geno	E	FlowerPosn	2	46	0.6498	0.6498	0.7756	0.6940
Geno	E	FlowerPosn	3	47	0.2472	0.2472	0.4903	0.3473
Geno	E	FlowerPosn	4	48	0.2465	0.2465	0.4900	0.3468
Geno	E	FlowerPosn	5	49	0.5496	0.5496	0.6938	0.6013
Geno	E	FlowerPosn	6	50	0.4251	0.4251	0.6001	0.4902
Geno	E	FlowerPosn	7	51	0.2465	0.2465	0.4900	0.3468
Geno	E	FlowerPosn	8	52	0.3455	0.3455	0.5465	0.4230
Geno	E	FlowerPosn	9	53	0.5486	0.5486	0.6930	0.6004
Geno	E	FlowerPosn	10	54	0.5481	0.5481	0.6926	0.5999
Geno	E	FlowerPosn	15	55	0.5486	0.5486	0.6930	0.6004
Geno	F	FlowerPosn	1	56	0.6949	0.6949	0.8137	0.7364
Geno	F	FlowerPosn	2	57	0.6948	0.6948	0.8136	0.7364
Geno	F	FlowerPosn	3	58	0.6009	0.6009	0.7351	0.6485
Geno	F	FlowerPosn	4	59	0.3473	0.3473	0.5477	0.4244
Geno	F	FlowerPosn	5	60	0.4912	0.4912	0.6486	0.5485
Geno	F	FlowerPosn	6	61	0.3490	0.3490	0.5488	0.4259
Geno	F	FlowerPosn	7	62	0.5486	0.5486	0.6930	0.6004
Geno	F	FlowerPosn	8	63	0.2440	0.2440	0.4887	0.3450
Geno	F	FlowerPosn	9	64	0.6017	0.6017	0.7358	0.6493
Geno	F	FlowerPosn	10	65	0.7364	0.7364	0.8495	0.7758
Geno	F	FlowerPosn	15	66	0.6482	0.6482	0.7742	0.6926
Geno	G	FlowerPosn	1	67	0.3473	0.3473	0.5477	0.4244
Geno	G	FlowerPosn	2	68	0.6937	0.6937	0.8127	0.7353
Geno	G	FlowerPosn	3	69	0.4263	0.4263	0.6008	0.4911
Geno	G	FlowerPosn	4	70	0.4898	0.4898	0.6475	0.5472
Geno	G	FlowerPosn	5	71	0.0027	0.0027	0.4235	0.2440
Geno	G	FlowerPosn	6	72	0.4234	0.4234	0.5988	0.4886
Geno	G	FlowerPosn	7	73	0.2440	0.2440	0.4887	0.3450
Geno	G	FlowerPosn	8	74	0.4251	0.4251	0.6001	0.4902
Geno	G	FlowerPosn	9	75	0.4251	0.4251	0.6001	0.4902
Geno	G	FlowerPosn	10	76	0.5491	0.5491	0.6934	0.6008
Geno	G	FlowerPosn	15	77	0.7363	0.7363	0.8494	0.7756
Geno	H	FlowerPosn	1	78	0.5481	0.5481	0.6926	0.5999
Geno	H	FlowerPosn	2	79	0.5486	0.5486	0.6930	0.6004
Geno	H	FlowerPosn	3	80	0.0027	0.0027	0.4235	0.2440
Geno	H	FlowerPosn	4	81	0.0027	0.0027	0.4235	0.2440
Geno	H	FlowerPosn	5	82	0.4251	0.4251	0.6001	0.4902
Geno	H	FlowerPosn	6	83	0.2465	0.2465	0.4900	0.3468
Geno	H	FlowerPosn	7	84	0.6493	0.6493	0.7752	0.6936

Geno H FlowerPosn 8	85	0.2465	0.2465	0.4900	0.3468
Geno H FlowerPosn 9	86	0.5492	0.5492	0.6935	0.6009
Geno H FlowerPosn 10	87	0.6489	0.6489	0.7749	0.6933
Geno H FlowerPosn 15	88	0.5476	0.5476	0.6922	0.5995

		17	18	19	20
Geno B FlowerPosn 10	21	*			
Geno B FlowerPosn 15	22	1.0011	*		
Geno C FlowerPosn 1	23	0.8845	1.0320	*	
Geno C FlowerPosn 2	24	0.8490	1.0017	0.8853	*
Geno C FlowerPosn 3	25	0.7347	0.9069	0.7763	0.7356
Geno C FlowerPosn 4	26	0.6485	0.8386	0.6953	0.6495
Geno C FlowerPosn 5	27	0.8484	1.0012	0.8847	0.8491
Geno C FlowerPosn 6	28	0.6922	0.8728	0.7362	0.6931
Geno C FlowerPosn 7	29	0.6935	0.8738	0.7374	0.6944
Geno C FlowerPosn 8	30	0.8491	1.0019	0.8854	0.8499
Geno C FlowerPosn 9	31	0.8125	0.9710	0.8503	0.8133
Geno C FlowerPosn 10	32	0.8489	1.0016	0.8851	0.8496
Geno C FlowerPosn 15	33	0.7740	0.9390	0.8136	0.7748
Geno D FlowerPosn 1	34	0.9183	1.0611	0.9519	0.9190
Geno D FlowerPosn 2	35	0.7756	0.9404	0.8151	0.7765
Geno D FlowerPosn 3	36	0.6478	0.8380	0.6946	0.6488
Geno D FlowerPosn 4	37	0.6928	0.8733	0.7368	0.6938
Geno D FlowerPosn 5	38	0.6475	0.8378	0.6944	0.6485
Geno D FlowerPosn 6	39	0.7748	0.9397	0.8143	0.7756
Geno D FlowerPosn 7	40	0.7740	0.9390	0.8136	0.7748
Geno D FlowerPosn 8	41	0.6922	0.8728	0.7362	0.6931
Geno D FlowerPosn 9	42	0.8128	0.9713	0.8506	0.8136
Geno D FlowerPosn 10	43	0.6485	0.8386	0.6953	0.6495
Geno D FlowerPosn 15	44	0.8841	1.0317	0.9190	0.8848
Geno E FlowerPosn 1	45	0.8847	1.0322	0.9195	0.8854
Geno E FlowerPosn 2	46	0.8843	1.0318	0.9191	0.8850
Geno E FlowerPosn 3	47	0.6487	0.8388	0.6955	0.6497
Geno E FlowerPosn 4	48	0.6485	0.8386	0.6953	0.6495
Geno E FlowerPosn 5	49	0.8135	0.9718	0.8513	0.8143
Geno E FlowerPosn 6	50	0.7352	0.9073	0.7768	0.7361
Geno E FlowerPosn 7	51	0.6485	0.8386	0.6953	0.6495
Geno E FlowerPosn 8	52	0.6922	0.8728	0.7362	0.6931
Geno E FlowerPosn 9	53	0.8128	0.9713	0.8506	0.8136
Geno E FlowerPosn 10	54	0.8125	0.9710	0.8503	0.8133
Geno E FlowerPosn 15	55	0.8128	0.9713	0.8506	0.8136
Geno F FlowerPosn 1	56	0.9179	1.0608	0.9516	0.9186
Geno F FlowerPosn 2	57	0.9179	1.0607	0.9515	0.9186
Geno F FlowerPosn 3	58	0.8490	1.0018	0.8853	0.8498
Geno F FlowerPosn 4	59	0.6931	0.8735	0.7370	0.6940
Geno F FlowerPosn 5	60	0.7753	0.9401	0.8148	0.7761
Geno F FlowerPosn 6	61	0.6940	0.8742	0.7379	0.6949
Geno F FlowerPosn 7	62	0.8129	0.9713	0.8507	0.8137
Geno F FlowerPosn 8	63	0.6475	0.8378	0.6944	0.6485
Geno F FlowerPosn 9	64	0.8496	1.0023	0.8858	0.8504
Geno F FlowerPosn 10	65	0.9498	1.0885	0.9823	0.9505
Geno F FlowerPosn 15	66	0.8831	1.0308	0.9180	0.8838
Geno G FlowerPosn 1	67	0.6931	0.8735	0.7370	0.6940
Geno G FlowerPosn 2	68	0.9170	1.0600	0.9507	0.9177
Geno G FlowerPosn 3	69	0.7358	0.9078	0.7774	0.7367
Geno G FlowerPosn 4	70	0.7744	0.9393	0.8140	0.7752
Geno G FlowerPosn 5	71	0.5998	0.8015	0.6501	0.6009
Geno G FlowerPosn 6	72	0.7342	0.9065	0.7758	0.7351
Geno G FlowerPosn 7	73	0.6475	0.8378	0.6944	0.6485
Geno G FlowerPosn 8	74	0.7352	0.9073	0.7768	0.7361
Geno G FlowerPosn 9	75	0.7352	0.9073	0.7768	0.7361
Geno G FlowerPosn 10	76	0.8132	0.9716	0.8509	0.8140
Geno G FlowerPosn 15	77	0.9497	1.0884	0.9822	0.9503
Geno H FlowerPosn 1	78	0.8125	0.9710	0.8503	0.8133
Geno H FlowerPosn 2	79	0.8129	0.9713	0.8507	0.8137
Geno H FlowerPosn 3	80	0.5998	0.8015	0.6501	0.6009
Geno H FlowerPosn 4	81	0.5998	0.8015	0.6501	0.6009
Geno H FlowerPosn 5	82	0.7352	0.9073	0.7768	0.7361

Geno	H	FlowerPosn	6	83	0.6485	0.8386	0.6953	0.6495
Geno	H	FlowerPosn	7	84	0.8840	1.0315	0.9188	0.8847
Geno	H	FlowerPosn	8	85	0.6485	0.8386	0.6953	0.6495
Geno	H	FlowerPosn	9	86	0.8132	0.9716	0.8510	0.8140
Geno	H	FlowerPosn	10	87	0.8837	1.0313	0.9186	0.8844
Geno	H	FlowerPosn	15	88	0.8122	0.9707	0.8500	0.8129
					21	22	23	24
Geno	C	FlowerPosn	3	25	*			
Geno	C	FlowerPosn	4	26	0.4907	*		
Geno	C	FlowerPosn	5	27	0.7349	0.6486	*	
Geno	C	FlowerPosn	6	28	0.5472	0.4244	0.6924	*
Geno	C	FlowerPosn	7	29	0.5488	0.4265	0.6936	0.4904
Geno	C	FlowerPosn	8	30	0.7357	0.6496	0.8493	0.6933
Geno	C	FlowerPosn	9	31	0.6931	0.6009	0.8126	0.6479
Geno	C	FlowerPosn	10	32	0.7354	0.6492	0.8490	0.6929
Geno	C	FlowerPosn	15	33	0.6476	0.5478	0.7741	0.5989
Geno	D	FlowerPosn	1	34	0.8146	0.7378	0.9184	0.7765
Geno	D	FlowerPosn	2	35	0.6495	0.5501	0.7758	0.6010
Geno	D	FlowerPosn	3	36	0.4898	0.3473	0.6479	0.4234
Geno	D	FlowerPosn	4	37	0.5480	0.4254	0.6930	0.4895
Geno	D	FlowerPosn	5	38	0.4894	0.3468	0.6477	0.4230
Geno	D	FlowerPosn	6	39	0.6485	0.5489	0.7749	0.5999
Geno	D	FlowerPosn	7	40	0.6476	0.5478	0.7741	0.5989
Geno	D	FlowerPosn	8	41	0.5472	0.4244	0.6924	0.4887
Geno	D	FlowerPosn	9	42	0.6935	0.6014	0.8130	0.6483
Geno	D	FlowerPosn	10	43	0.4907	0.3486	0.6486	0.4244
Geno	D	FlowerPosn	15	44	0.7758	0.6947	0.8842	0.7357
Geno	E	FlowerPosn	1	45	0.7765	0.6954	0.8848	0.7364
Geno	E	FlowerPosn	2	46	0.7760	0.6949	0.8844	0.7359
Geno	E	FlowerPosn	3	47	0.4910	0.3490	0.6489	0.4248
Geno	E	FlowerPosn	4	48	0.4907	0.3486	0.6486	0.4244
Geno	E	FlowerPosn	5	49	0.6943	0.6023	0.8136	0.6492
Geno	E	FlowerPosn	6	50	0.6007	0.4914	0.7353	0.5478
Geno	E	FlowerPosn	7	51	0.4907	0.3486	0.6486	0.4244
Geno	E	FlowerPosn	8	52	0.5472	0.4244	0.6924	0.4886
Geno	E	FlowerPosn	9	53	0.6935	0.6014	0.8130	0.6483
Geno	E	FlowerPosn	10	54	0.6931	0.6009	0.8126	0.6479
Geno	E	FlowerPosn	15	55	0.6935	0.6014	0.8130	0.6483
Geno	F	FlowerPosn	1	56	0.8142	0.7373	0.9180	0.7760
Geno	F	FlowerPosn	2	57	0.8141	0.7372	0.9180	0.7759
Geno	F	FlowerPosn	3	58	0.7356	0.6495	0.8492	0.6932
Geno	F	FlowerPosn	4	59	0.5483	0.4259	0.6932	0.4899
Geno	F	FlowerPosn	5	60	0.6491	0.5496	0.7754	0.6006
Geno	F	FlowerPosn	6	61	0.5494	0.4273	0.6941	0.4911
Geno	F	FlowerPosn	7	62	0.6936	0.6014	0.8130	0.6484
Geno	F	FlowerPosn	8	63	0.4894	0.3468	0.6477	0.4230
Geno	F	FlowerPosn	9	64	0.7363	0.6502	0.8497	0.6939
Geno	F	FlowerPosn	10	65	0.8499	0.7766	0.9499	0.8135
Geno	F	FlowerPosn	15	66	0.7747	0.6935	0.8832	0.7345
Geno	G	FlowerPosn	1	67	0.5483	0.4258	0.6932	0.4899
Geno	G	FlowerPosn	2	68	0.8132	0.7362	0.9171	0.7750
Geno	G	FlowerPosn	3	69	0.6014	0.4924	0.7360	0.5487
Geno	G	FlowerPosn	4	70	0.6480	0.5483	0.7745	0.5994
Geno	G	FlowerPosn	5	71	0.4243	0.2465	0.6000	0.3455
Geno	G	FlowerPosn	6	72	0.5994	0.4899	0.7343	0.5465
Geno	G	FlowerPosn	7	73	0.4894	0.3468	0.6477	0.4230
Geno	G	FlowerPosn	8	74	0.6007	0.4914	0.7353	0.5478
Geno	G	FlowerPosn	9	75	0.6007	0.4914	0.7353	0.5478
Geno	G	FlowerPosn	10	76	0.6939	0.6018	0.8133	0.6487
Geno	G	FlowerPosn	15	77	0.8498	0.7764	0.9498	0.8133
Geno	H	FlowerPosn	1	78	0.6931	0.6009	0.8126	0.6479
Geno	H	FlowerPosn	2	79	0.6936	0.6014	0.8130	0.6484
Geno	H	FlowerPosn	3	80	0.4243	0.2465	0.6000	0.3455
Geno	H	FlowerPosn	4	81	0.4243	0.2465	0.6000	0.3455
Geno	H	FlowerPosn	5	82	0.6007	0.4914	0.7353	0.5478
Geno	H	FlowerPosn	6	83	0.4907	0.3486	0.6486	0.4244
Geno	H	FlowerPosn	7	84	0.7757	0.6945	0.8841	0.7355

Geno	H	FlowerPosn	8	85	0.4907	0.3486	0.6486	0.4244
Geno	H	FlowerPosn	9	86	0.6940	0.6019	0.8134	0.6488
Geno	H	FlowerPosn	10	87	0.7753	0.6942	0.8838	0.7352
Geno	H	FlowerPosn	15	88	0.6927	0.6005	0.8123	0.6475
					25	26	27	28

Geno	C	FlowerPosn	7	29	*			
Geno	C	FlowerPosn	8	30	0.6946	*		
Geno	C	FlowerPosn	9	31	0.6492	0.8134	*	
Geno	C	FlowerPosn	10	32	0.6942	0.8497	0.8131	*
Geno	C	FlowerPosn	15	33	0.6003	0.7750	0.7346	0.7746
Geno	D	FlowerPosn	1	34	0.7776	0.9191	0.8854	0.9189
Geno	D	FlowerPosn	2	35	0.6025	0.7766	0.7363	0.7763
Geno	D	FlowerPosn	3	36	0.4254	0.6489	0.6002	0.6486
Geno	D	FlowerPosn	4	37	0.4913	0.6939	0.6486	0.6936
Geno	D	FlowerPosn	5	38	0.4250	0.6487	0.5999	0.6483
Geno	D	FlowerPosn	6	39	0.6014	0.7758	0.7355	0.7754
Geno	D	FlowerPosn	7	40	0.6003	0.7750	0.7346	0.7746
Geno	D	FlowerPosn	8	41	0.4904	0.6933	0.6479	0.6929
Geno	D	FlowerPosn	9	42	0.6497	0.8138	0.7754	0.8134
Geno	D	FlowerPosn	10	43	0.4265	0.6496	0.6009	0.6492
Geno	D	FlowerPosn	15	44	0.7369	0.8850	0.8498	0.8847
Geno	E	FlowerPosn	1	45	0.7376	0.8855	0.8505	0.8853
Geno	E	FlowerPosn	2	46	0.7371	0.8851	0.8500	0.8849
Geno	E	FlowerPosn	3	47	0.4269	0.6499	0.6012	0.6495
Geno	E	FlowerPosn	4	48	0.4265	0.6496	0.6009	0.6492
Geno	E	FlowerPosn	5	49	0.6505	0.8144	0.7761	0.8141
Geno	E	FlowerPosn	6	50	0.5494	0.7362	0.6936	0.7359
Geno	E	FlowerPosn	7	51	0.4265	0.6496	0.6009	0.6492
Geno	E	FlowerPosn	8	52	0.4904	0.6933	0.6479	0.6929
Geno	E	FlowerPosn	9	53	0.6497	0.8138	0.7754	0.8134
Geno	E	FlowerPosn	10	54	0.6492	0.8134	0.7751	0.8131
Geno	E	FlowerPosn	15	55	0.6497	0.8138	0.7754	0.8134
Geno	F	FlowerPosn	1	56	0.7771	0.9188	0.8850	0.9185
Geno	F	FlowerPosn	2	57	0.7771	0.9187	0.8849	0.9184
Geno	F	FlowerPosn	3	58	0.6944	0.8499	0.8133	0.8496
Geno	F	FlowerPosn	4	59	0.4917	0.6942	0.6488	0.6938
Geno	F	FlowerPosn	5	60	0.6020	0.7763	0.7360	0.7759
Geno	F	FlowerPosn	6	61	0.4929	0.6951	0.6498	0.6947
Geno	F	FlowerPosn	7	62	0.6497	0.8138	0.7755	0.8135
Geno	F	FlowerPosn	8	63	0.4250	0.6487	0.5999	0.6483
Geno	F	FlowerPosn	9	64	0.6951	0.8505	0.8139	0.8502
Geno	F	FlowerPosn	10	65	0.8145	0.9506	0.9180	0.9503
Geno	F	FlowerPosn	15	66	0.7357	0.8840	0.8488	0.8837
Geno	G	FlowerPosn	1	67	0.4917	0.6942	0.6488	0.6938
Geno	G	FlowerPosn	2	68	0.7761	0.9179	0.8841	0.9176
Geno	G	FlowerPosn	3	69	0.5503	0.7369	0.6943	0.7365
Geno	G	FlowerPosn	4	70	0.6008	0.7753	0.7350	0.7750
Geno	G	FlowerPosn	5	71	0.3480	0.6011	0.5481	0.6006
Geno	G	FlowerPosn	6	72	0.5481	0.7352	0.6925	0.7349
Geno	G	FlowerPosn	7	73	0.4250	0.6487	0.5999	0.6483
Geno	G	FlowerPosn	8	74	0.5494	0.7362	0.6936	0.7359
Geno	G	FlowerPosn	9	75	0.5494	0.7362	0.6936	0.7359
Geno	G	FlowerPosn	10	76	0.6501	0.8141	0.7758	0.8138
Geno	G	FlowerPosn	15	77	0.8144	0.9504	0.9179	0.9502
Geno	H	FlowerPosn	1	78	0.6492	0.8134	0.7751	0.8131
Geno	H	FlowerPosn	2	79	0.6497	0.8138	0.7755	0.8135
Geno	H	FlowerPosn	3	80	0.3480	0.6011	0.5481	0.6006
Geno	H	FlowerPosn	4	81	0.3480	0.6011	0.5481	0.6006
Geno	H	FlowerPosn	5	82	0.5494	0.7362	0.6936	0.7359
Geno	H	FlowerPosn	6	83	0.4265	0.6496	0.6009	0.6492
Geno	H	FlowerPosn	7	84	0.7367	0.8848	0.8497	0.8845
Geno	H	FlowerPosn	8	85	0.4265	0.6496	0.6009	0.6492
Geno	H	FlowerPosn	9	86	0.6502	0.8142	0.7759	0.8139
Geno	H	FlowerPosn	10	87	0.7364	0.8845	0.8494	0.8843
Geno	H	FlowerPosn	15	88	0.6488	0.8131	0.7747	0.8128
					29	30	31	32

Geno C FlowerPosn 15	33	*				
Geno D FlowerPosn 1	34	0.8502	*			
Geno D FlowerPosn 2	35	0.6936	0.8517	*		
Geno D FlowerPosn 3	36	0.5469	0.7372	0.5493	*	
Geno D FlowerPosn 4	37	0.5996	0.7770	0.6017	0.4244	
Geno D FlowerPosn 5	38	0.5466	0.7369	0.5490	0.3455	
Geno D FlowerPosn 6	39	0.6927	0.8509	0.6945	0.5481	
Geno D FlowerPosn 7	40	0.6918	0.8502	0.6936	0.5469	
Geno D FlowerPosn 8	41	0.5989	0.7765	0.6010	0.4234	
Geno D FlowerPosn 9	42	0.7350	0.8857	0.7367	0.6006	
Geno D FlowerPosn 10	43	0.5478	0.7378	0.5501	0.3473	
Geno D FlowerPosn 15	44	0.8131	0.9515	0.8147	0.6941	
Geno E FlowerPosn 1	45	0.8138	0.9521	0.8153	0.6948	
Geno E FlowerPosn 2	46	0.8133	0.9517	0.8149	0.6943	
Geno E FlowerPosn 3	47	0.5481	0.7380	0.5504	0.3478	
Geno E FlowerPosn 4	48	0.5478	0.7378	0.5501	0.3473	
Geno E FlowerPosn 5	49	0.7357	0.8863	0.7375	0.6016	
Geno E FlowerPosn 6	50	0.6481	0.8150	0.6501	0.4905	
Geno E FlowerPosn 7	51	0.5478	0.7378	0.5501	0.3473	
Geno E FlowerPosn 8	52	0.5989	0.7765	0.6010	0.4234	
Geno E FlowerPosn 9	53	0.7350	0.8857	0.7367	0.6006	
Geno E FlowerPosn 10	54	0.7346	0.8854	0.7363	0.6002	
Geno E FlowerPosn 15	55	0.7350	0.8857	0.7367	0.6006	
Geno F FlowerPosn 1	56	0.8498	0.9830	0.8513	0.7367	
Geno F FlowerPosn 2	57	0.8497	0.9830	0.8512	0.7366	
Geno F FlowerPosn 3	58	0.7748	0.9190	0.7765	0.6488	
Geno F FlowerPosn 4	59	0.5999	0.7773	0.6020	0.4248	
Geno F FlowerPosn 5	60	0.6932	0.8514	0.6951	0.5488	
Geno F FlowerPosn 6	61	0.6009	0.7781	0.6030	0.4263	
Geno F FlowerPosn 7	62	0.7350	0.8857	0.7368	0.6007	
Geno F FlowerPosn 8	63	0.5466	0.7369	0.5490	0.3455	
Geno F FlowerPosn 9	64	0.7755	0.9196	0.7771	0.6496	
Geno F FlowerPosn 10	65	0.8841	1.0129	0.8855	0.7760	
Geno F FlowerPosn 15	66	0.8120	0.9506	0.8136	0.6928	
Geno G FlowerPosn 1	67	0.5999	0.7773	0.6020	0.4248	
Geno G FlowerPosn 2	68	0.8488	0.9822	0.8503	0.7356	
Geno G FlowerPosn 3	69	0.6488	0.8156	0.6508	0.4915	
Geno G FlowerPosn 4	70	0.6922	0.8505	0.6941	0.5475	
Geno G FlowerPosn 5	71	0.4892	0.6954	0.4918	0.2447	
Geno G FlowerPosn 6	72	0.6469	0.8141	0.6489	0.4890	
Geno G FlowerPosn 7	73	0.5466	0.7369	0.5490	0.3455	
Geno G FlowerPosn 8	74	0.6481	0.8150	0.6501	0.4905	
Geno G FlowerPosn 9	75	0.6481	0.8150	0.6501	0.4905	
Geno G FlowerPosn 10	76	0.7354	0.8860	0.7371	0.6011	
Geno G FlowerPosn 15	77	0.8840	1.0127	0.8854	0.7758	
Geno H FlowerPosn 1	78	0.7346	0.8854	0.7363	0.6002	
Geno H FlowerPosn 2	79	0.7350	0.8857	0.7368	0.6007	
Geno H FlowerPosn 3	80	0.4892	0.6954	0.4918	0.2447	
Geno H FlowerPosn 4	81	0.4892	0.6954	0.4918	0.2447	
Geno H FlowerPosn 5	82	0.6481	0.8150	0.6501	0.4905	
Geno H FlowerPosn 6	83	0.5478	0.7378	0.5501	0.3473	
Geno H FlowerPosn 7	84	0.8130	0.9514	0.8145	0.6939	
Geno H FlowerPosn 8	85	0.5478	0.7378	0.5501	0.3473	
Geno H FlowerPosn 9	86	0.7355	0.8861	0.7372	0.6012	
Geno H FlowerPosn 10	87	0.8127	0.9511	0.8142	0.6935	
Geno H FlowerPosn 15	88	0.7342	0.8851	0.7360	0.5997	
		33	34	35	36	

Geno D FlowerPosn 4	37	*			
Geno D FlowerPosn 5	38	0.4240	*		
Geno D FlowerPosn 6	39	0.6007	0.5478	*	
Geno D FlowerPosn 7	40	0.5996	0.5466	0.6927	*
Geno D FlowerPosn 8	41	0.4895	0.4230	0.5999	0.5989
Geno D FlowerPosn 9	42	0.6490	0.6004	0.7358	0.7350
Geno D FlowerPosn 10	43	0.4254	0.3468	0.5489	0.5478
Geno D FlowerPosn 15	44	0.7363	0.6938	0.8139	0.8131
Geno E FlowerPosn 1	45	0.7370	0.6946	0.8145	0.8138
Geno E FlowerPosn 2	46	0.7365	0.6940	0.8141	0.8133

Geno E FlowerPosn	3	47	0.4259	0.3473	0.5492	0.5481
Geno E FlowerPosn	4	48	0.4254	0.3468	0.5489	0.5478
Geno E FlowerPosn	5	49	0.6498	0.6013	0.7366	0.7357
Geno E FlowerPosn	6	50	0.5486	0.4902	0.6490	0.6481
Geno E FlowerPosn	7	51	0.4254	0.3468	0.5489	0.5478
Geno E FlowerPosn	8	52	0.4895	0.4230	0.5999	0.5989
Geno E FlowerPosn	9	53	0.6490	0.6004	0.7358	0.7350
Geno E FlowerPosn	10	54	0.6486	0.5999	0.7355	0.7346
Geno E FlowerPosn	15	55	0.6490	0.6004	0.7358	0.7350
Geno F FlowerPosn	1	56	0.7766	0.7364	0.8505	0.8498
Geno F FlowerPosn	2	57	0.7765	0.7364	0.8504	0.8497
Geno F FlowerPosn	3	58	0.6938	0.6485	0.7756	0.7748
Geno F FlowerPosn	4	59	0.4908	0.4244	0.6009	0.5999
Geno F FlowerPosn	5	60	0.6013	0.5485	0.6941	0.6932
Geno F FlowerPosn	6	61	0.4920	0.4259	0.6020	0.6009
Geno F FlowerPosn	7	62	0.6490	0.6004	0.7359	0.7350
Geno F FlowerPosn	8	63	0.4240	0.3450	0.5478	0.5466
Geno F FlowerPosn	9	64	0.6945	0.6493	0.7763	0.7755
Geno F FlowerPosn	10	65	0.8140	0.7758	0.8848	0.8841
Geno F FlowerPosn	15	66	0.7351	0.6926	0.8128	0.8120
Geno G FlowerPosn	1	67	0.4908	0.4244	0.6009	0.5999
Geno G FlowerPosn	2	68	0.7755	0.7353	0.8495	0.8488
Geno G FlowerPosn	3	69	0.5495	0.4911	0.6498	0.6488
Geno G FlowerPosn	4	70	0.6001	0.5472	0.6931	0.6922
Geno G FlowerPosn	5	71	0.3468	0.2440	0.4904	0.4892
Geno G FlowerPosn	6	72	0.5473	0.4886	0.6479	0.6469
Geno G FlowerPosn	7	73	0.4240	0.3450	0.5478	0.5466
Geno G FlowerPosn	8	74	0.5486	0.4902	0.6490	0.6481
Geno G FlowerPosn	9	75	0.5486	0.4902	0.6490	0.6481
Geno G FlowerPosn	10	76	0.6494	0.6008	0.7362	0.7354
Geno G FlowerPosn	15	77	0.8138	0.7756	0.8846	0.8840
Geno H FlowerPosn	1	78	0.6486	0.5999	0.7355	0.7346
Geno H FlowerPosn	2	79	0.6490	0.6004	0.7359	0.7350
Geno H FlowerPosn	3	80	0.3468	0.2440	0.4904	0.4892
Geno H FlowerPosn	4	81	0.3468	0.2440	0.4904	0.4892
Geno H FlowerPosn	5	82	0.5486	0.4902	0.6490	0.6481
Geno H FlowerPosn	6	83	0.4254	0.3468	0.5489	0.5478
Geno H FlowerPosn	7	84	0.7361	0.6936	0.8137	0.8130
Geno H FlowerPosn	8	85	0.4254	0.3468	0.5489	0.5478
Geno H FlowerPosn	9	86	0.6495	0.6009	0.7363	0.7355
Geno H FlowerPosn	10	87	0.7358	0.6933	0.8134	0.8127
Geno H FlowerPosn	15	88	0.6481	0.5995	0.7351	0.7342

			37	38	39	40
Geno D FlowerPosn	8	41	*			
Geno D FlowerPosn	9	42	0.6483	*		
Geno D FlowerPosn	10	43	0.4244	0.6014	*	
Geno D FlowerPosn	15	44	0.7357	0.8502	0.6947	*
Geno E FlowerPosn	1	45	0.7364	0.8508	0.6954	0.9191
Geno E FlowerPosn	2	46	0.7359	0.8504	0.6949	0.9187
Geno E FlowerPosn	3	47	0.4248	0.6017	0.3490	0.6950
Geno E FlowerPosn	4	48	0.4244	0.6014	0.3486	0.6947
Geno E FlowerPosn	5	49	0.6492	0.7765	0.6023	0.8508
Geno E FlowerPosn	6	50	0.5478	0.6940	0.4914	0.7763
Geno E FlowerPosn	7	51	0.4244	0.6014	0.3486	0.6947
Geno E FlowerPosn	8	52	0.4886	0.6483	0.4244	0.7357
Geno E FlowerPosn	9	53	0.6483	0.7758	0.6014	0.8502
Geno E FlowerPosn	10	54	0.6479	0.7754	0.6009	0.8498
Geno E FlowerPosn	15	55	0.6483	0.7758	0.6014	0.8502
Geno F FlowerPosn	1	56	0.7760	0.8853	0.7373	0.9512
Geno F FlowerPosn	2	57	0.7759	0.8852	0.7372	0.9511
Geno F FlowerPosn	3	58	0.6932	0.8136	0.6495	0.8849
Geno F FlowerPosn	4	59	0.4899	0.6493	0.4259	0.7365
Geno F FlowerPosn	5	60	0.6006	0.7364	0.5496	0.8144
Geno F FlowerPosn	6	61	0.4911	0.6502	0.4273	0.7374
Geno F FlowerPosn	7	62	0.6484	0.7758	0.6014	0.8502
Geno F FlowerPosn	8	63	0.4230	0.6004	0.3468	0.6938
Geno F FlowerPosn	9	64	0.6939	0.8142	0.6502	0.8854

Geno F FlowerPosn 10	65	0.8135	0.9183	0.7766	0.9819
Geno F FlowerPosn 15	66	0.7345	0.8491	0.6935	0.9176
Geno G FlowerPosn 1	67	0.4899	0.6492	0.4258	0.7365
Geno G FlowerPosn 2	68	0.7750	0.8844	0.7362	0.9503
Geno G FlowerPosn 3	69	0.5487	0.6947	0.4924	0.7769
Geno G FlowerPosn 4	70	0.5994	0.7354	0.5483	0.8135
Geno G FlowerPosn 5	71	0.3455	0.5486	0.2465	0.6495
Geno G FlowerPosn 6	72	0.5465	0.6929	0.4899	0.7753
Geno G FlowerPosn 7	73	0.4230	0.6004	0.3468	0.6938
Geno G FlowerPosn 8	74	0.5478	0.6940	0.4914	0.7763
Geno G FlowerPosn 9	75	0.5478	0.6940	0.4914	0.7763
Geno G FlowerPosn 10	76	0.6487	0.7761	0.6018	0.8505
Geno G FlowerPosn 15	77	0.8133	0.9182	0.7764	0.9818
Geno H FlowerPosn 1	78	0.6479	0.7754	0.6009	0.8498
Geno H FlowerPosn 2	79	0.6484	0.7758	0.6014	0.8502
Geno H FlowerPosn 3	80	0.3455	0.5486	0.2465	0.6495
Geno H FlowerPosn 4	81	0.3455	0.5486	0.2465	0.6495
Geno H FlowerPosn 5	82	0.5478	0.6940	0.4914	0.7763
Geno H FlowerPosn 6	83	0.4244	0.6014	0.3486	0.6947
Geno H FlowerPosn 7	84	0.7355	0.8500	0.6945	0.9184
Geno H FlowerPosn 8	85	0.4244	0.6014	0.3486	0.6947
Geno H FlowerPosn 9	86	0.6488	0.7762	0.6019	0.8506
Geno H FlowerPosn 10	87	0.7352	0.8497	0.6942	0.9181
Geno H FlowerPosn 15	88	0.6475	0.7751	0.6005	0.8495

		41	42	43	44
Geno E FlowerPosn 1	45	*			
Geno E FlowerPosn 2	46	0.9193	*		
Geno E FlowerPosn 3	47	0.6957	0.6952	*	
Geno E FlowerPosn 4	48	0.6954	0.6949	0.3490	*
Geno E FlowerPosn 5	49	0.8514	0.8510	0.6026	0.6023
Geno E FlowerPosn 6	50	0.7769	0.7765	0.4918	0.4914
Geno E FlowerPosn 7	51	0.6954	0.6949	0.3490	0.3486
Geno E FlowerPosn 8	52	0.7364	0.7359	0.4248	0.4244
Geno E FlowerPosn 9	53	0.8508	0.8504	0.6017	0.6014
Geno E FlowerPosn 10	54	0.8505	0.8500	0.6012	0.6009
Geno E FlowerPosn 15	55	0.8508	0.8504	0.6017	0.6014
Geno F FlowerPosn 1	56	0.9517	0.9513	0.7375	0.7373
Geno F FlowerPosn 2	57	0.9516	0.9513	0.7374	0.7372
Geno F FlowerPosn 3	58	0.8854	0.8850	0.6498	0.6495
Geno F FlowerPosn 4	59	0.7372	0.7367	0.4263	0.4259
Geno F FlowerPosn 5	60	0.8150	0.8145	0.5499	0.5496
Geno F FlowerPosn 6	61	0.7381	0.7376	0.4277	0.4273
Geno F FlowerPosn 7	62	0.8508	0.8504	0.6017	0.6014
Geno F FlowerPosn 8	63	0.6946	0.6940	0.3473	0.3468
Geno F FlowerPosn 9	64	0.8860	0.8856	0.6505	0.6502
Geno F FlowerPosn 10	65	0.9825	0.9821	0.7768	0.7766
Geno F FlowerPosn 15	66	0.9182	0.9178	0.6937	0.6935
Geno G FlowerPosn 1	67	0.7372	0.7367	0.4263	0.4258
Geno G FlowerPosn 2	68	0.9508	0.9505	0.7364	0.7362
Geno G FlowerPosn 3	69	0.7776	0.7771	0.4927	0.4924
Geno G FlowerPosn 4	70	0.8141	0.8137	0.5486	0.5483
Geno G FlowerPosn 5	71	0.6503	0.6498	0.2472	0.2465
Geno G FlowerPosn 6	72	0.7760	0.7755	0.4902	0.4899
Geno G FlowerPosn 7	73	0.6946	0.6940	0.3473	0.3468
Geno G FlowerPosn 8	74	0.7769	0.7765	0.4918	0.4914
Geno G FlowerPosn 9	75	0.7769	0.7765	0.4918	0.4914
Geno G FlowerPosn 10	76	0.8511	0.8507	0.6021	0.6018
Geno G FlowerPosn 15	77	0.9823	0.9820	0.7766	0.7764
Geno H FlowerPosn 1	78	0.8505	0.8500	0.6012	0.6009
Geno H FlowerPosn 2	79	0.8508	0.8504	0.6017	0.6014
Geno H FlowerPosn 3	80	0.6503	0.6498	0.2472	0.2465
Geno H FlowerPosn 4	81	0.6503	0.6498	0.2472	0.2465
Geno H FlowerPosn 5	82	0.7769	0.7765	0.4918	0.4914
Geno H FlowerPosn 6	83	0.6954	0.6949	0.3490	0.3486
Geno H FlowerPosn 7	84	0.9190	0.9186	0.6948	0.6945
Geno H FlowerPosn 8	85	0.6954	0.6949	0.3490	0.3486
Geno H FlowerPosn 9	86	0.8512	0.8508	0.6022	0.6019

Geno H FlowerPosn 10	87	0.9187	0.9183	0.6944	0.6942
Geno H FlowerPosn 15	88	0.8501	0.8497	0.6008	0.6005
		45	46	47	48

Geno E FlowerPosn 5	49	*			
Geno E FlowerPosn 6	50	0.6948	*		
Geno E FlowerPosn 7	51	0.6023	0.4914	*	
Geno E FlowerPosn 8	52	0.6491	0.5478	0.4244	*
Geno E FlowerPosn 9	53	0.7765	0.6940	0.6014	0.6483
Geno E FlowerPosn 10	54	0.7761	0.6936	0.6009	0.6479
Geno E FlowerPosn 15	55	0.7765	0.6940	0.6014	0.6483
Geno F FlowerPosn 1	56	0.8859	0.8146	0.7373	0.7760
Geno F FlowerPosn 2	57	0.8858	0.8145	0.7372	0.7759
Geno F FlowerPosn 3	58	0.8143	0.7361	0.6495	0.6932
Geno F FlowerPosn 4	59	0.6501	0.5490	0.4259	0.4899
Geno F FlowerPosn 5	60	0.7371	0.6497	0.5496	0.6006
Geno F FlowerPosn 6	61	0.6510	0.5501	0.4273	0.4911
Geno F FlowerPosn 7	62	0.7765	0.6941	0.6014	0.6483
Geno F FlowerPosn 8	63	0.6013	0.4902	0.3468	0.4230
Geno F FlowerPosn 9	64	0.8149	0.7368	0.6502	0.6939
Geno F FlowerPosn 10	65	0.9189	0.8503	0.7766	0.8135
Geno F FlowerPosn 15	66	0.8498	0.7752	0.6935	0.7345
Geno G FlowerPosn 1	67	0.6501	0.5489	0.4258	0.4899
Geno G FlowerPosn 2	68	0.8850	0.8136	0.7362	0.7750
Geno G FlowerPosn 3	69	0.6955	0.6020	0.4924	0.5487
Geno G FlowerPosn 4	70	0.7361	0.6486	0.5483	0.5994
Geno G FlowerPosn 5	71	0.5496	0.4251	0.2465	0.3455
Geno G FlowerPosn 6	72	0.6937	0.6000	0.4899	0.5465
Geno G FlowerPosn 7	73	0.6013	0.4902	0.3468	0.4230
Geno G FlowerPosn 8	74	0.6948	0.6012	0.4914	0.5478
Geno G FlowerPosn 9	75	0.6948	0.6012	0.4914	0.5478
Geno G FlowerPosn 10	76	0.7768	0.6944	0.6018	0.6487
Geno G FlowerPosn 15	77	0.9187	0.8502	0.7764	0.8133
Geno H FlowerPosn 1	78	0.7761	0.6936	0.6009	0.6479
Geno H FlowerPosn 2	79	0.7765	0.6941	0.6014	0.6484
Geno H FlowerPosn 3	80	0.5496	0.4251	0.2465	0.3455
Geno H FlowerPosn 4	81	0.5496	0.4251	0.2465	0.3455
Geno H FlowerPosn 5	82	0.6948	0.6012	0.4914	0.5478
Geno H FlowerPosn 6	83	0.6023	0.4914	0.3486	0.4244
Geno H FlowerPosn 7	84	0.8507	0.7761	0.6945	0.7355
Geno H FlowerPosn 8	85	0.6023	0.4914	0.3486	0.4244
Geno H FlowerPosn 9	86	0.7769	0.6945	0.6019	0.6488
Geno H FlowerPosn 10	87	0.8504	0.7758	0.6942	0.7352
Geno H FlowerPosn 15	88	0.7758	0.6932	0.6005	0.6475
		49	50	51	52

Geno E FlowerPosn 9	53	*			
Geno E FlowerPosn 10	54	0.7754	*		
Geno E FlowerPosn 15	55	0.7758	0.7754	*	
Geno F FlowerPosn 1	56	0.8853	0.8850	0.8853	*
Geno F FlowerPosn 2	57	0.8852	0.8849	0.8852	0.9826
Geno F FlowerPosn 3	58	0.8136	0.8133	0.8136	0.9187
Geno F FlowerPosn 4	59	0.6493	0.6488	0.6493	0.7768
Geno F FlowerPosn 5	60	0.7364	0.7360	0.7364	0.8510
Geno F FlowerPosn 6	61	0.6502	0.6498	0.6502	0.7776
Geno F FlowerPosn 7	62	0.7758	0.7755	0.7758	0.8853
Geno F FlowerPosn 8	63	0.6004	0.5999	0.6004	0.7364
Geno F FlowerPosn 9	64	0.8142	0.8139	0.8142	0.9192
Geno F FlowerPosn 10	65	0.9183	0.9180	0.9183	1.0125
Geno F FlowerPosn 15	66	0.8491	0.8488	0.8491	0.9502
Geno G FlowerPosn 1	67	0.6492	0.6488	0.6492	0.7768
Geno G FlowerPosn 2	68	0.8844	0.8841	0.8844	0.9818
Geno G FlowerPosn 3	69	0.6947	0.6943	0.6947	0.8152
Geno G FlowerPosn 4	70	0.7354	0.7350	0.7354	0.8501
Geno G FlowerPosn 5	71	0.5486	0.5481	0.5486	0.6949
Geno G FlowerPosn 6	72	0.6929	0.6925	0.6929	0.8137
Geno G FlowerPosn 7	73	0.6004	0.5999	0.6004	0.7364
Geno G FlowerPosn 8	74	0.6940	0.6936	0.6940	0.8146

Geno G FlowerPosn 9	75	0.6940	0.6936	0.6940	0.8146
Geno G FlowerPosn 10	76	0.7761	0.7758	0.7761	0.8856
Geno G FlowerPosn 15	77	0.9182	0.9179	0.9182	1.0124
Geno H FlowerPosn 1	78	0.7754	0.7751	0.7754	0.8850
Geno H FlowerPosn 2	79	0.7758	0.7755	0.7758	0.8853
Geno H FlowerPosn 3	80	0.5486	0.5481	0.5486	0.6949
Geno H FlowerPosn 4	81	0.5486	0.5481	0.5486	0.6949
Geno H FlowerPosn 5	82	0.6940	0.6936	0.6940	0.8146
Geno H FlowerPosn 6	83	0.6014	0.6009	0.6014	0.7373
Geno H FlowerPosn 7	84	0.8500	0.8497	0.8500	0.9510
Geno H FlowerPosn 8	85	0.6014	0.6009	0.6014	0.7373
Geno H FlowerPosn 9	86	0.7762	0.7759	0.7762	0.8857
Geno H FlowerPosn 10	87	0.8497	0.8494	0.8497	0.9508
Geno H FlowerPosn 15	88	0.7751	0.7747	0.7751	0.8847
		53	54	55	56

Geno F FlowerPosn 2	57	*			
Geno F FlowerPosn 3	58	0.9186	*		
Geno F FlowerPosn 4	59	0.7767	0.6940	*	
Geno F FlowerPosn 5	60	0.8509	0.7761	0.6016	*
Geno F FlowerPosn 6	61	0.7775	0.6949	0.4924	0.6026
Geno F FlowerPosn 7	62	0.8853	0.8137	0.6493	0.7364
Geno F FlowerPosn 8	63	0.7364	0.6485	0.4244	0.5485
Geno F FlowerPosn 9	64	0.9191	0.8504	0.6948	0.7768
Geno F FlowerPosn 10	65	1.0124	0.9505	0.8142	0.8852
Geno F FlowerPosn 15	66	0.9502	0.8839	0.7353	0.8133
Geno G FlowerPosn 1	67	0.7767	0.6940	0.4911	0.6016
Geno G FlowerPosn 2	68	0.9818	0.9178	0.7757	0.8500
Geno G FlowerPosn 3	69	0.8151	0.7367	0.5498	0.6504
Geno G FlowerPosn 4	70	0.8500	0.7752	0.6004	0.6937
Geno G FlowerPosn 5	71	0.6948	0.6009	0.3473	0.4912
Geno G FlowerPosn 6	72	0.8136	0.7351	0.5476	0.6485
Geno G FlowerPosn 7	73	0.7364	0.6485	0.4244	0.5485
Geno G FlowerPosn 8	74	0.8145	0.7361	0.5490	0.6497
Geno G FlowerPosn 9	75	0.8145	0.7361	0.5490	0.6497
Geno G FlowerPosn 10	76	0.8855	0.8140	0.6497	0.7367
Geno G FlowerPosn 15	77	1.0123	0.9504	0.8141	0.8851
Geno H FlowerPosn 1	78	0.8849	0.8133	0.6488	0.7360
Geno H FlowerPosn 2	79	0.8853	0.8137	0.6493	0.7364
Geno H FlowerPosn 3	80	0.6948	0.6009	0.3473	0.4912
Geno H FlowerPosn 4	81	0.6948	0.6009	0.3473	0.4912
Geno H FlowerPosn 5	82	0.8145	0.7361	0.5490	0.6497
Geno H FlowerPosn 6	83	0.7372	0.6495	0.4259	0.5496
Geno H FlowerPosn 7	84	0.9509	0.8847	0.7363	0.8142
Geno H FlowerPosn 8	85	0.7372	0.6495	0.4259	0.5496
Geno H FlowerPosn 9	86	0.8856	0.8141	0.6498	0.7368
Geno H FlowerPosn 10	87	0.9507	0.8844	0.7360	0.8139
Geno H FlowerPosn 15	88	0.8846	0.8130	0.6484	0.7356
		57	58	59	60

Geno F FlowerPosn 6	61	*			
Geno F FlowerPosn 7	62	0.6502	*		
Geno F FlowerPosn 8	63	0.4259	0.6004	*	
Geno F FlowerPosn 9	64	0.6956	0.8143	0.6493	*
Geno F FlowerPosn 10	65	0.8150	0.9183	0.7758	0.9510
Geno F FlowerPosn 15	66	0.7362	0.8492	0.6926	0.8844
Geno G FlowerPosn 1	67	0.4924	0.6493	0.4244	0.6947
Geno G FlowerPosn 2	68	0.7765	0.8844	0.7353	0.9183
Geno G FlowerPosn 3	69	0.5509	0.6947	0.4911	0.7374
Geno G FlowerPosn 4	70	0.6014	0.7354	0.5472	0.7759
Geno G FlowerPosn 5	71	0.3490	0.5486	0.2440	0.6017
Geno G FlowerPosn 6	72	0.5487	0.6930	0.4886	0.7357
Geno G FlowerPosn 7	73	0.4259	0.6004	0.3450	0.6493
Geno G FlowerPosn 8	74	0.5501	0.6941	0.4902	0.7368
Geno G FlowerPosn 9	75	0.5501	0.6941	0.4902	0.7368
Geno G FlowerPosn 10	76	0.6506	0.7762	0.6008	0.8146
Geno G FlowerPosn 15	77	0.8148	0.9182	0.7756	0.9509
Geno H FlowerPosn 1	78	0.6498	0.7755	0.5999	0.8139

Geno H FlowerPosn 2	79	0.6502	0.7759	0.6004	0.8143
Geno H FlowerPosn 3	80	0.3490	0.5486	0.2440	0.6017
Geno H FlowerPosn 4	81	0.3490	0.5486	0.2440	0.6017
Geno H FlowerPosn 5	82	0.5501	0.6941	0.4902	0.7368
Geno H FlowerPosn 6	83	0.4273	0.6014	0.3468	0.6502
Geno H FlowerPosn 7	84	0.7372	0.8500	0.6936	0.8853
Geno H FlowerPosn 8	85	0.4273	0.6014	0.3468	0.6502
Geno H FlowerPosn 9	86	0.6507	0.7763	0.6009	0.8147
Geno H FlowerPosn 10	87	0.7369	0.8498	0.6933	0.8850
Geno H FlowerPosn 15	88	0.6493	0.7751	0.5995	0.8136
		61	62	63	64

Geno F FlowerPosn 10	65	*			
Geno F FlowerPosn 15	66	0.9811	*		
Geno G FlowerPosn 1	67	0.8142	0.7353	*	
Geno G FlowerPosn 2	68	1.0117	0.9494	0.7757	*
Geno G FlowerPosn 3	69	0.8509	0.7758	0.5498	0.8142
Geno G FlowerPosn 4	70	0.8844	0.8124	0.6004	0.8492
Geno G FlowerPosn 5	71	0.7364	0.6482	0.3473	0.6937
Geno G FlowerPosn 6	72	0.8495	0.7742	0.5476	0.8127
Geno G FlowerPosn 7	73	0.7758	0.6926	0.4244	0.7353
Geno G FlowerPosn 8	74	0.8503	0.7752	0.5489	0.8136
Geno G FlowerPosn 9	75	0.8503	0.7752	0.5489	0.8136
Geno G FlowerPosn 10	76	0.9186	0.8495	0.6497	0.8847
Geno G FlowerPosn 15	77	1.0414	0.9809	0.8141	1.0116
Geno H FlowerPosn 1	78	0.9180	0.8488	0.6488	0.8841
Geno H FlowerPosn 2	79	0.9183	0.8492	0.6493	0.8844
Geno H FlowerPosn 3	80	0.7364	0.6482	0.3473	0.6937
Geno H FlowerPosn 4	81	0.7364	0.6482	0.3473	0.6937
Geno H FlowerPosn 5	82	0.8503	0.7752	0.5489	0.8136
Geno H FlowerPosn 6	83	0.7766	0.6935	0.4258	0.7362
Geno H FlowerPosn 7	84	0.9818	0.9175	0.7363	0.9501
Geno H FlowerPosn 8	85	0.7766	0.6935	0.4258	0.7362
Geno H FlowerPosn 9	86	0.9187	0.8495	0.6498	0.8847
Geno H FlowerPosn 10	87	0.9816	0.9172	0.7360	0.9499
Geno H FlowerPosn 15	88	0.9177	0.8485	0.6484	0.8837
		65	66	67	68

Geno G FlowerPosn 3	69	*			
Geno G FlowerPosn 4	70	0.6493	*		
Geno G FlowerPosn 5	71	0.4263	0.4898	*	
Geno G FlowerPosn 6	72	0.6008	0.6474	0.4234	*
Geno G FlowerPosn 7	73	0.4911	0.5472	0.2440	0.4886
Geno G FlowerPosn 8	74	0.6020	0.6486	0.4251	0.6000
Geno G FlowerPosn 9	75	0.6020	0.6486	0.4251	0.6000
Geno G FlowerPosn 10	76	0.6951	0.7358	0.5491	0.6933
Geno G FlowerPosn 15	77	0.8507	0.8843	0.7363	0.8493
Geno H FlowerPosn 1	78	0.6943	0.7350	0.5481	0.6925
Geno H FlowerPosn 2	79	0.6947	0.7354	0.5486	0.6930
Geno H FlowerPosn 3	80	0.4263	0.4898	0.0027	0.4234
Geno H FlowerPosn 4	81	0.4263	0.4898	0.0027	0.4234
Geno H FlowerPosn 5	82	0.6020	0.6486	0.4251	0.6000
Geno H FlowerPosn 6	83	0.4924	0.5483	0.2465	0.4899
Geno H FlowerPosn 7	84	0.7767	0.8133	0.6493	0.7751
Geno H FlowerPosn 8	85	0.4924	0.5483	0.2465	0.4899
Geno H FlowerPosn 9	86	0.6952	0.7359	0.5492	0.6934
Geno H FlowerPosn 10	87	0.7764	0.8130	0.6489	0.7748
Geno H FlowerPosn 15	88	0.6939	0.7347	0.5476	0.6922
		69	70	71	72

Geno G FlowerPosn 7	73	*			
Geno G FlowerPosn 8	74	0.4902	*		
Geno G FlowerPosn 9	75	0.4902	0.6012	*	
Geno G FlowerPosn 10	76	0.6008	0.6944	0.6944	*
Geno G FlowerPosn 15	77	0.7756	0.8502	0.8502	0.9184
Geno H FlowerPosn 1	78	0.5999	0.6936	0.6936	0.7758
Geno H FlowerPosn 2	79	0.6004	0.6941	0.6941	0.7762
Geno H FlowerPosn 3	80	0.2440	0.4251	0.4251	0.5491

Geno H FlowerPosn 4	81	0.2440	0.4251	0.4251	0.5491
Geno H FlowerPosn 5	82	0.4902	0.6012	0.6012	0.6944
Geno H FlowerPosn 6	83	0.3468	0.4914	0.4914	0.6018
Geno H FlowerPosn 7	84	0.6936	0.7761	0.7761	0.8503
Geno H FlowerPosn 8	85	0.3468	0.4914	0.4914	0.6018
Geno H FlowerPosn 9	86	0.6009	0.6945	0.6945	0.7766
Geno H FlowerPosn 10	87	0.6933	0.7758	0.7758	0.8501
Geno H FlowerPosn 15	88	0.5995	0.6932	0.6932	0.7754
		73	74	75	76

Geno G FlowerPosn 15	77	*			
Geno H FlowerPosn 1	78	0.9179	*		
Geno H FlowerPosn 2	79	0.9182	0.7755	*	
Geno H FlowerPosn 3	80	0.7363	0.5481	0.5486	*
Geno H FlowerPosn 4	81	0.7363	0.5481	0.5486	0.0027
Geno H FlowerPosn 5	82	0.8502	0.6936	0.6941	0.4251
Geno H FlowerPosn 6	83	0.7764	0.6009	0.6014	0.2465
Geno H FlowerPosn 7	84	0.9817	0.8497	0.8500	0.6493
Geno H FlowerPosn 8	85	0.7764	0.6009	0.6014	0.2465
Geno H FlowerPosn 9	86	0.9185	0.7759	0.7763	0.5492
Geno H FlowerPosn 10	87	0.9814	0.8494	0.8498	0.6489
Geno H FlowerPosn 15	88	0.9176	0.7747	0.7751	0.5476
		77	78	79	80

Geno H FlowerPosn 4	81	*			
Geno H FlowerPosn 5	82	0.4251	*		
Geno H FlowerPosn 6	83	0.2465	0.4914	*	
Geno H FlowerPosn 7	84	0.6493	0.7761	0.6945	*
Geno H FlowerPosn 8	85	0.2465	0.4914	0.3486	0.6945
Geno H FlowerPosn 9	86	0.5492	0.6945	0.6019	0.8504
Geno H FlowerPosn 10	87	0.6489	0.7758	0.6942	0.9180
Geno H FlowerPosn 15	88	0.5476	0.6932	0.6005	0.8494
		81	82	83	84

Geno H FlowerPosn 8	85	*			
Geno H FlowerPosn 9	86	0.6019	*		
Geno H FlowerPosn 10	87	0.6942	0.8501	*	
Geno H FlowerPosn 15	88	0.6005	0.7755	0.8491	*
		85	86	87	88

Genotypes are: A (Wild Type Col-0), B (*ga20ox1*), C (*ga20ox2*), D (*ga20ox3*), E (*ga20ox1 ga20ox2*), F (*ga20ox1 ga20ox3*), G (*ga20ox2 ga20ox3*), H (*ga20ox1 ga20ox2 ga20ox3*).

3c. Predicted mean frequencies of floral abnormalities (averaged across all genotypes), S.E. and LSD (5%) values for comparison, arising from the significant interaction between flower position and GA treatments ($p < 0.001$, Fig. 4c).

Flower	1		2	
	Prediction	s.e.	Prediction	s.e.
GA				
GA-	0.7504	0.1532	0.5625	0.1326
GA+	0.7217	0.1502	0.7503	0.1532

Flower	3		4	
	Prediction	s.e.	Prediction	s.e.
GA				
GA-	0.2820	0.0939	0.0626	0.0443
GA+	0.2816	0.0939	0.3445	0.1037

Flower	5		6	
	Prediction	s.e.	Prediction	s.e.
GA				
GA-	0.0931	0.0540	0.0626	0.0443
GA+	0.5332	0.1292	0.4378	0.1170

Flower	7		8	
	Prediction	s.e.	Prediction	s.e.
GA				
GA-	0.0626	0.0443	0.0310	0.0311
GA+	0.5943	0.1364	0.5301	0.1287

Flower	9		10	
	Prediction	s.e.	Prediction	s.e.
GA				
GA-	0.0936	0.0541	0.1247	0.0625
GA+	0.9077	0.1685	1.2187	0.1951

Flower	15	
	Prediction	s.e.
GA		
GA-	0.2183	0.0827
GA+	1.3424	0.2109

Least significant differences of predictions (5% level) (526 df)

GA	GA-	Flower	1	1	*			
GA	GA-	Flower	2	2	0.3981	*		
GA	GA-	Flower	3	3	0.3531	0.3192	*	
GA	GA-	Flower	4	4	0.3133	0.2746	0.2040	*
GA	GA-	Flower	5	5	0.3192	0.2812	0.2128	0.1371
GA	GA-	Flower	6	6	0.3133	0.2746	0.2040	0.1230
GA	GA-	Flower	7	7	0.3133	0.2746	0.2040	0.1230
GA	GA-	Flower	8	8	0.3072	0.2676	0.1944	0.1063
GA	GA-	Flower	9	9	0.3193	0.2813	0.2130	0.1373
GA	GA-	Flower	10	10	0.3251	0.2879	0.2216	0.1504
GA	GA-	Flower	15	11	0.3420	0.3069	0.2458	0.1842
GA	GA+	Flower	1	12	0.4216	0.3937	0.3481	0.3077
GA	GA+	Flower	2	13	0.4256	0.3980	0.3529	0.3132
GA	GA+	Flower	3	14	0.3530	0.3192	0.2609	0.2039
GA	GA+	Flower	4	15	0.3635	0.3307	0.2749	0.2215
GA	GA+	Flower	5	16	0.3937	0.3636	0.3137	0.2682
GA	GA+	Flower	6	17	0.3788	0.3474	0.2948	0.2458
GA	GA+	Flower	7	18	0.4030	0.3737	0.3253	0.2817
GA	GA+	Flower	8	19	0.3931	0.3630	0.3130	0.2673
GA	GA+	Flower	9	20	0.4474	0.4212	0.3790	0.3423
GA	GA+	Flower	10	21	0.4874	0.4634	0.4254	0.3930
GA	GA+	Flower	15	22	0.5121	0.4894	0.4536	0.4233
					1	2	3	4

GA GA- Flower	5	5	*			
GA GA- Flower	6	6	0.1371	*		
GA GA- Flower	7	7	0.1371	0.1230	*	
GA GA- Flower	8	8	0.1224	0.1063	0.1063	*
GA GA- Flower	9	9	0.1502	0.1373	0.1373	0.1226
GA GA- Flower	10	10	0.1622	0.1504	0.1504	0.1371
GA GA- Flower	15	11	0.1939	0.1842	0.1842	0.1735
GA GA+ Flower	1	12	0.3136	0.3077	0.3077	0.3014
GA GA+ Flower	2	13	0.3190	0.3132	0.3132	0.3070
GA GA+ Flower	3	14	0.2127	0.2039	0.2039	0.1943
GA GA+ Flower	4	15	0.2297	0.2215	0.2215	0.2127
GA GA+ Flower	5	16	0.2750	0.2682	0.2682	0.2610
GA GA+ Flower	6	17	0.2532	0.2458	0.2458	0.2379
GA GA+ Flower	7	18	0.2881	0.2817	0.2817	0.2748
GA GA+ Flower	8	19	0.2741	0.2673	0.2673	0.2601
GA GA+ Flower	9	20	0.3476	0.3423	0.3423	0.3366
GA GA+ Flower	10	21	0.3977	0.3930	0.3930	0.3882
GA GA+ Flower	15	22	0.4277	0.4233	0.4233	0.4188
			5	6	7	8

GA GA- Flower	9	9	*			
GA GA- Flower	10	10	0.1624	*		
GA GA- Flower	15	11	0.1941	0.2035	*	
GA GA+ Flower	1	12	0.3137	0.3197	0.3369	*
GA GA+ Flower	2	13	0.3191	0.3249	0.3419	0.4215
GA GA+ Flower	3	14	0.2129	0.2215	0.2457	0.3480
GA GA+ Flower	4	15	0.2298	0.2378	0.2605	0.3587
GA GA+ Flower	5	16	0.2751	0.2819	0.3013	0.3892
GA GA+ Flower	6	17	0.2533	0.2606	0.2815	0.3741
GA GA+ Flower	7	18	0.2882	0.2947	0.3133	0.3986
GA GA+ Flower	8	19	0.2742	0.2810	0.3004	0.3886
GA GA+ Flower	9	20	0.3477	0.3530	0.3687	0.4435
GA GA+ Flower	10	21	0.3978	0.4025	0.4163	0.4838
GA GA+ Flower	15	22	0.4277	0.4321	0.4450	0.5087
			9	10	11	12

GA GA+ Flower	2	13	*			
GA GA+ Flower	3	14	0.3529	*		
GA GA+ Flower	4	15	0.3634	0.2748	*	
GA GA+ Flower	5	16	0.3936	0.3137	0.3254	*
GA GA+ Flower	6	17	0.3787	0.2947	0.3072	0.3424
GA GA+ Flower	7	18	0.4029	0.3252	0.3366	0.3690
GA GA+ Flower	8	19	0.3930	0.3129	0.3247	0.3582
GA GA+ Flower	9	20	0.4473	0.3789	0.3887	0.4171
GA GA+ Flower	10	21	0.4873	0.4254	0.4341	0.4597
GA GA+ Flower	15	22	0.5120	0.4535	0.4617	0.4859
			13	14	15	16

GA GA+ Flower	6	17	*			
GA GA+ Flower	7	18	0.3530	*		
GA GA+ Flower	8	19	0.3417	0.3683	*	
GA GA+ Flower	9	20	0.4030	0.4259	0.4165	*
GA GA+ Flower	10	21	0.4470	0.4676	0.4591	0.5065
GA GA+ Flower	15	22	0.4738	0.4934	0.4853	0.5303
			17	18	19	20

GA GA+ Flower	10	21	*	
GA GA+ Flower	15	22	0.5644	*
			21	22

Predicted means for flower position by GA interaction for floral abnormalities (see Fig. 4c), SEs, and LSD (5%) values for comparisons. Genotypes are: A (Wild Type Col-0), B (*ga20ox1*), C (*ga20ox2*), D (*ga20ox3*), E (*ga20ox1 ga20ox2*), F (*ga20ox1 ga20ox3*), G (*ga20ox2 ga20ox3*), H (*ga20ox1 ga20ox2 ga20ox3*).
