# Supplementary material for: The early inflorescence of Arabidopsis thaliana demonstrates positional effects in floral organ growth and meristem patterning
Source: Plant Reprod. 2017 Dec 20;31(2):171–91. doi: 10.1007/s00497-017-0320-3 (PMC5940708; doi:10.1007/s00497-017-0320-3)
Supplement: Supplementary file 4 — Supplementary material 4 (PDF 968 kb) [file 497_2017_320_MOESM4_ESM.pdf]

ONLINE RESOURCE 4: The effect of exogenous GA treatment on floral organ lengths during early flowering.

Article Title: The early inflorescence of *Arabidopsis thaliana* demonstrates positional effects in floral organ growth and meristem patterning

Journal: Plant Reproduction

Authors: ARG Plackett, SJ Powers, AL Phillips, ZA Wilson, P Hedden, SG Thomas

Corresponding author: ARG Plackett
Address: University of Cambridge, Department of Plant Sciences, Downing Street, Cambridge, CB2 3EA, UK
E-mail: arp74@cam.ac.uk

4A. Primary inflorescence phenotypes of *ga20ox* mutants between flower positions 1 and 10 under control growth conditions and exogenous GA treatment.

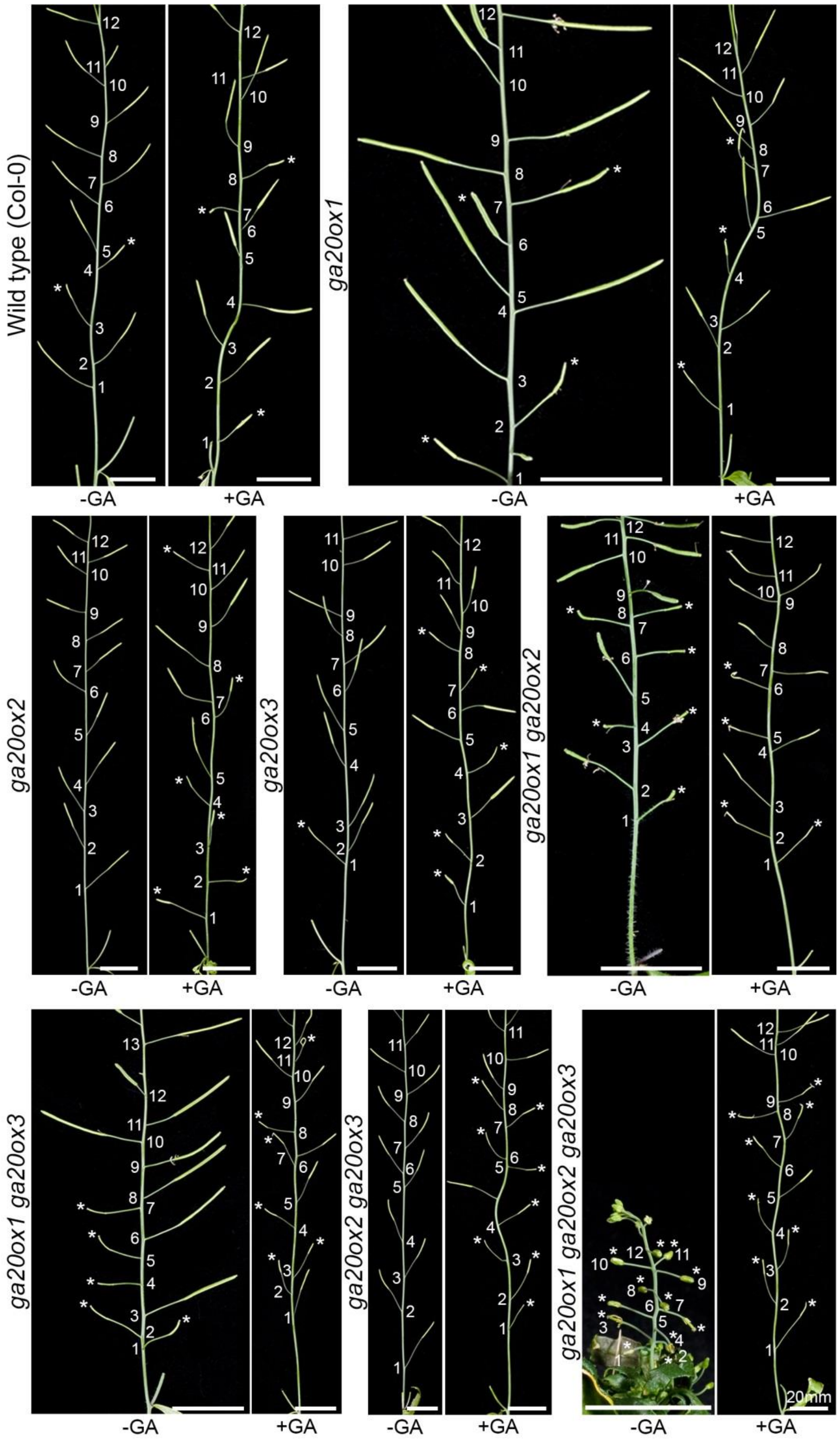

Silique-set was scored once siliques approached maturity. Flower positions are numbered from the base of the primary inflorescence. Asterisks indicate unpollinated flower positions

4B. Comparison of floral phenotypes at flower positions 1 and 10 under exogenous GA treatment.

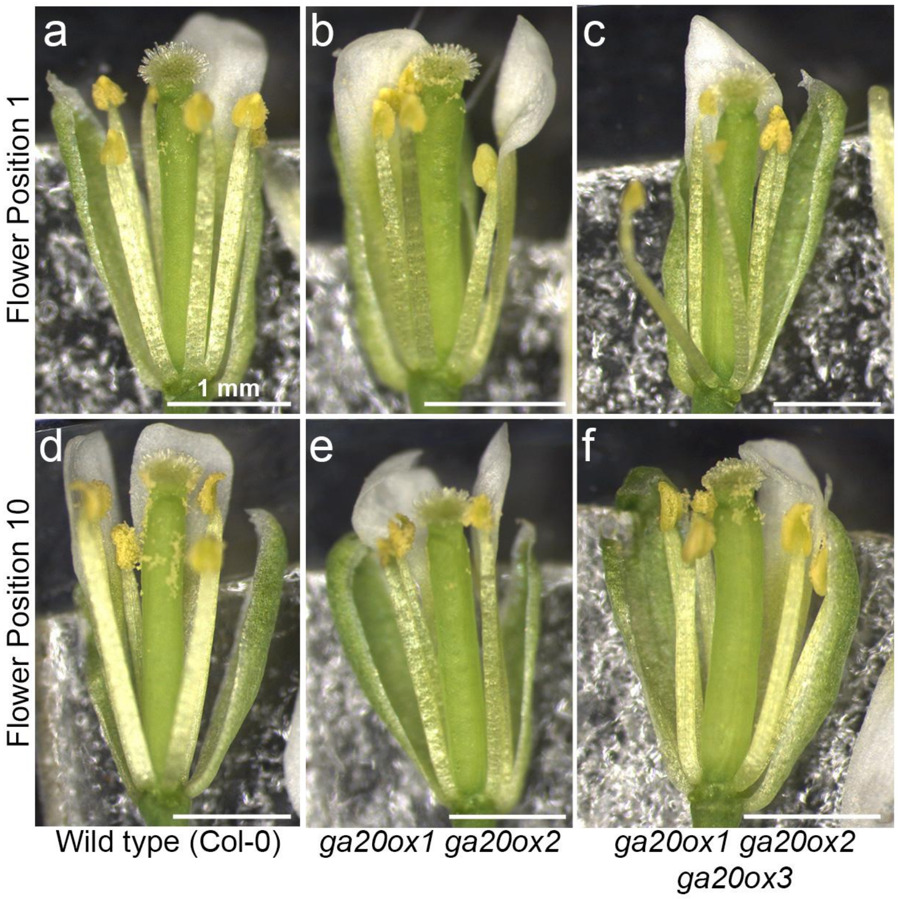

Genotypes compared are wild type (Col-0) (**a**, **d**), *ga20ox1 ga20ox2* (**b**, **e**) and *ga20ox1 ga20ox2 ga20ox3* (**c**, **f**). GA treatment is sufficient to restore relative floral organ growth to that seen in wild type (compare to Figure 2).

4C. Floral organ lengths across early flowering under exogenous GA treatment.

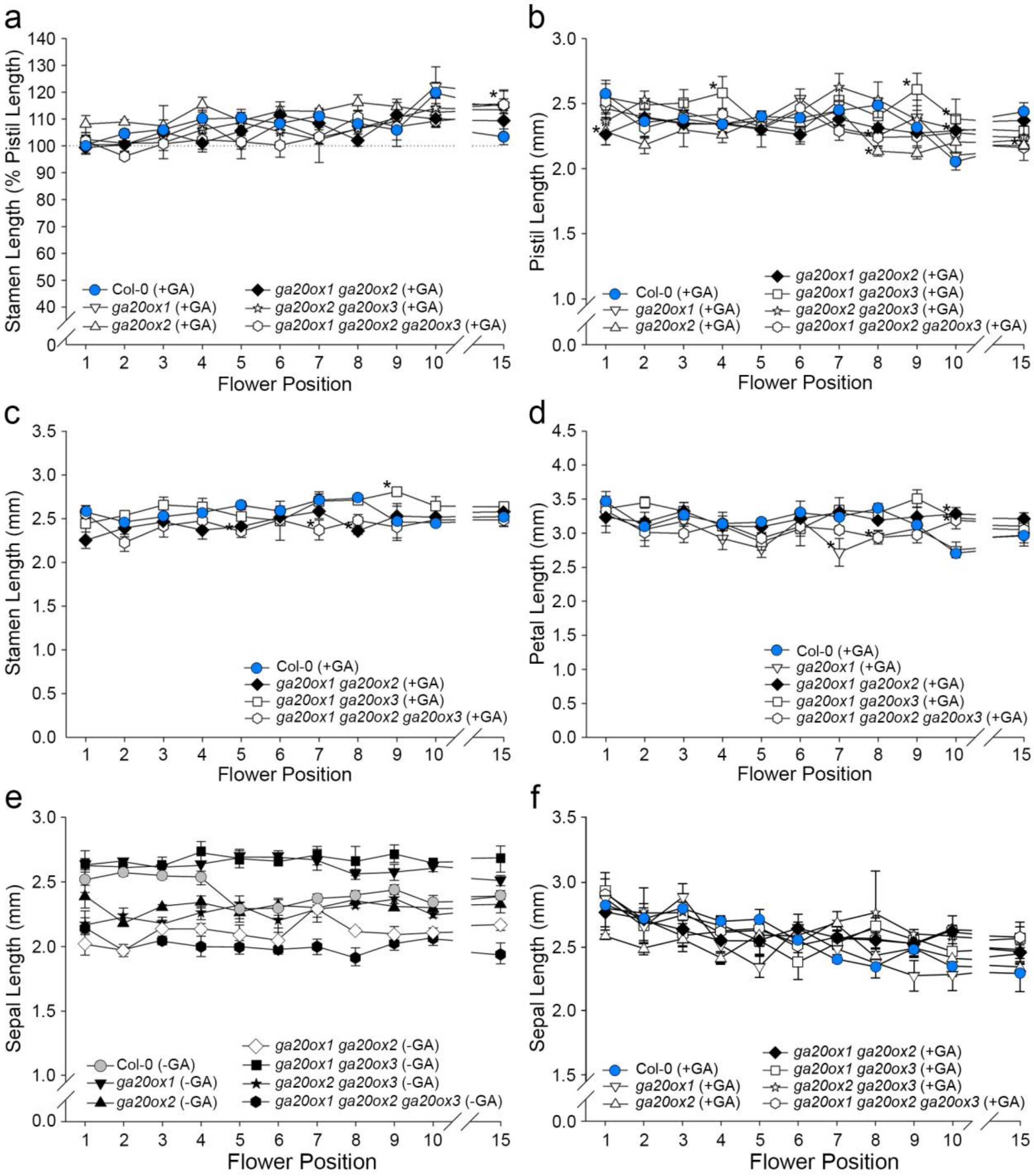

a-d. Mean floral organ lengths ±S.E. across the early inflorescence under exogenous GA treatment, floral organ type as indicated. Genotypes shown are those in which significant differences from wild type ($p < 0.05$) occur, denoted by asterisks. For ease of reference, wild type (Col-0) and *ga20ox1 ga20ox2* are highlighted in blue and black, respectively. Comparisons were restricted to within each flower position: LSD(5%) = 11.199 (**a**), 0.7157 (**b**), 0.09344 (**c**), 0.3975 (**d**). Statistical analysis on pistil and stamen absolute lengths were

performed on square-root transformed data to meet statistical assumptions (see Online Resource 2).

e-f. Plotted mean sepal length ±S.E. across the early inflorescence of individual genotypes (as indicated) under control growth conditions (**e**) and exogenous GA treatment (**f**). Pairwise comparisons are not statistically valid in the absence of a significant three-way interaction ($p = 0.275$).

4D. The effect of exogenous GA treatment on wild type (Col-0) floral organ lengths during early flowering.

Flower Position	Relative Stamen Length (%)		Pistil Length (mm)		Stamen Length (mm)		Petal Length (mm)		Sepal Length (mm)	
	-GA	+GA	-GA	+GA	-GA	+GA	-GA	+GA	-GA	+GA
1	100.96	100.43	2.333 [1.5273]	2.571 [1.6030]*	2.355 [1.5335]	2.578 [1.6051]	3.029	3.458*	2.51	2.82
2	106.36	104.58	2.408 [1.5510]	2.358 [1.5354]	2.557 [1.5985]	2.465 [1.5699]	3.268	3.089	2.57	2.71
3	107.20	106.07	2.442 [1.5624]	2.383 [1.5434]	2.619 [1.6178]	2.525 [1.5885]	3.327	3.271	2.54	2.79
4	109.48	110.05	2.387 [1.5446]	2.341 [1.5300]	2.614 [1.6163]	2.573 [1.6040]	3.505	3.141	2.53	2.68
5	108.02	110.50	2.246 [1.4986]	2.401 [1.5495]	2.425 [1.5567]	2.652 [1.6284]	3.055	3.161	2.29	2.71
6	112.10	108.39	2.246 [1.4975]	2.389 [1.5452]	2.508 [1.5832]	2.586 [1.6079]	3.104	3.298	2.3	2.55
7	112.62	111.16	2.334 [1.5277]	2.446 [1.5633]	2.631 [1.6204]	2.714 [1.6471]	3.209	3.243	2.37	2.41
8	113.46	108.39	2.227 [1.4917]	2.488 [1.5770]	2.531 [1.5839]	2.735 [1.6538]	3.076	3.348	2.39	2.34
9	111.90	105.96	2.395 [1.5475]	2.313 [1.5199]	2.680 [1.6368]	2.462 [1.5644]	3.487	3.121	2.44	2.48
10	124.95	119.73	2.352 [1.5330]	2.054 [1.4327]*	2.939 [1.7136]	2.453 [1.5660]*	3.596	2.707*	2.33	2.34
15	121.29	103.36*	2.418 [1.5544]	2.438 [1.5610]	2.926 [1.7106]	2.522 [1.5868]*	3.596	2.970*	2.39	2.29
LSD (5%)	*11.22*		*[0.07137]*		*[0.09281]*		*0.3931*		*n/a*	

Lengths shown are means from four newly-opened flowers (floral stage 13; Smyth et al. 1990). Where analysis was performed on transformed data to fit statistical assumptions (see materials and methods) these values are given in square brackets. Asterisks denote a significant difference ($p < 0.05$) between control growth conditions (–GA) and exogenous GA treatment (+GA) for that flower position. Comparisons were made using LSD values at 5% significance, as shown. Statistical comparisons of this nature were not valid (LSD *n/a*) for sepal length data as there was no statistically-significant three-way interaction between genotype, GA treatment and flower position ($p = 0.275$) (see Fig. 2).
